# Dynamics of sex chromosome evolution in a rapid radiation of cichlid fishes

**DOI:** 10.1126/sciadv.abe8215

**Published:** 2021-09-01

**Authors:** Athimed El Taher, Fabrizia Ronco, Michael Matschiner, Walter Salzburger, Astrid Böhne

**Affiliations:** 1 Zoological Institute, Department of Environmental Sciences, University of Basel, Basel, Switzerland.; 2 Department of Paleontology and Museum, University of Zurich, Zurich, Switzerland.; 3 Centre for Ecological and Evolutionary Synthesis (CEES), Department of Biosciences, University of Oslo, Oslo, Norway.; 4 Center for Molecular Biodiversity Research, Zoological Research Museum Alexander Koenig, Bonn, Germany.

## Abstract

Sex is a fundamental trait determined by environmental and/or genetic factors, including sex chromosomes. Sex chromosomes are studied in species scattered across the tree of life, yet little is known about tempo and mode of sex chromosome evolution among closely related species. Here, we examine sex chromosome evolution in the adaptive radiation of cichlid fishes in Lake Tanganyika. Through the analysis of male and female genomes from 244 cichlid taxa (189 described species with 5 represented with two local variants/populations; 50 undescribed species) and of 396 multitissue transcriptomes from 66 taxa, we identify signatures of sex chromosomes in 79 taxa, involving 12 linkage groups. We find that Tanganyikan cichlids have the highest rates of sex chromosome turnover and heterogamety transitions known to date. We show that sex chromosome recruitment is not at random. Moreover convergently emerged sex chromosomes in cichlids support the “limited options” hypothesis of sex chromosome evolution.

## Teaser

We show that sex chromosome changes are very frequent in Lake Tanganyika cichlids and repeatedly involve the same chromosomes.

## INTRODUCTION

Sex chromosomes—referred to as Z and W in female- and X and Y in male-heterogametic sex determination (SD) systems—define, through their properties and combinations, the sex of an individual (*1*). The evolutionary trajectories of sex chromosomes differ from those of autosomes: Because of the restriction of one of the two sex chromosomes to one sex (W to females in ZW female-heterogametic, Y to males in XY male-heterogametic SD systems), their sex-specific inheritance (e.g., XY fathers pass on their X exclusively to daughters and their Y to sons), and their reduced levels of recombination, sex chromosomes accumulate mutations more rapidly than autosomes, potentially leading to accelerated functional evolution (*2*).

The functioning of a chromosome as sex chromosome is often short-lived on evolutionary time scales, with prominent exceptions such as the conserved and strongly differentiated sex chromosomes of most mammals and birds (*3*). This relative evolutionary instability of sex chromosomes is due to turnovers, i.e., changes of the actual chromosome pair in use as sex chromosomes, caused by a new sex-determining mutation in a previously autosomal locus or the translocation of the ancestral SD gene to another chromosome. Sex chromosome turnovers may be accompanied by a transition in heterogamety (*4*). Heterogamety can also change without a transition in the chromosome pair that acts as sex chromosomes, which, in this case, likely involves a turnover of, or a mutation within, the actual SD locus.

The presumed major driving forces underlying turnovers of sex chromosomes are deleterious mutational load (*5*), sexually antagonistic loci linked to a newly invading SD gene (*6*, *7*), selection on restoring sex ratios (*8*), and genetic drift (*9*). These drivers are predicted to differ in their respective outcome: Turnovers induced by mutational load tend to preserve heterogamety (*5*), while sexually antagonistic selection–driven turnovers more readily induce a change of heterogamety (*6*). Last, the gene repertoire on previously existing sex chromosomes can also be extended by chromosomal fusion with an autosome, which then becomes sex-linked itself, leading to the formation of a neo-sex chromosome.

The frequency of occurrence of these different paths of sex chromosome evolution varies substantially across animal clades (*10*). For example, in some vertebrates (mammals and birds), the same sex chromosomes are shared across the entire class [but see (*11*)]. Models (*9*) and empirical observations (*12*) suggest that sex chromosomes such as those of mammals and (most) birds have differentiated to a degree that makes turnovers unlikely; these heteromorphic (that is, cytogenetically distinguishable) sex chromosomes are in an “evolutionary trap” (*13*). This is because a sex chromosome turnover requires the fixation of one of the previous sex chromosomes as an autosome, which becomes more deleterious and, hence, less likely the more specialized and/or degenerated the sex chromosomes are. In amphibians, reptiles, and fish, frequent turnover events and continued recombination led to many different and mostly nondegenerated, so-called homomorphic sex chromosomes (*14*, *15*). In contrast to the heteromorphic, strongly differentiated sex chromosomes of most mammals and birds, homomorphic sex chromosomes are not distinguishable by classical cytogenetics. Species with SD systems relying on homomorphic sex chromosomes nevertheless produce different types of gametes (differing by as little as a single allele) and thus fit into the common classification of female (ZW)– versus male (XY)–heterogametic SD systems.

To date, empirical studies on the dynamics of sex chromosome evolution are limited and scattered across different taxa. In an amphibian system with a rapid rate of sex chromosome turnover, the true frogs Ranidae, mutational load seems to be the major driving force of sex chromosome turnover (*15*). In geckos, a high rate of sex chromosome changes with heterogametic transitions potentially supports sexual antagonism as a key mechanism of these changes (*16*). However, an in-depth analysis of sex chromosome turnovers over short evolutionary time scales and with a broad taxon sampling is currently lacking (*10*).

Here, we examined sex chromosome evolution in a prime example of rapid organismal diversification, the adaptive radiation of cichlid fishes in African Lake Tanganyika (LT) (*17*). Teleost fishes are generally known for their species richness, but cichlids stand out in this clade on the basis of the “explosive” character of several of their adaptive radiations, giving rise to a total estimated number of over 3000 species (*18*). Rapid speciation in adaptive radiations is usually attributed to ecological specialization and thus diversification in ecomorphological traits (*17*). We were interested in whether the evolution of SD is keeping pace with other traits in cichlids by determining the diversity of SD systems and by investigating the dynamics of sex chromosome turnover across the entire LT cichlid radiation. The previously available data from about 30 African cichlid species [reviewed in (*19*, *20*)] suggest that sex chromosomes are not conserved in this group with both, simple, and polygenic SD systems as well as male and female heterogametic SD being known from the different species investigated. Cichlid sex chromosomes are largely presumed homomorphic, as no sex differences in karyotypes have been observed so far across a variety of species (*21*).

An emerging picture is that certain chromosomes have recurrently been recruited as sex chromosomes in cichlids. This observation could lend further support to the “limited options” hypothesis (*22*) that, based on a comparison of identified master SD genes and the patterns of recruitments of autosomes as sex chromosomes across different vertebrate lineages, suggests some genes or chromosomes to be intrinsically better suited at becoming sex determiners.

Within cichlids, support for the convergent recruitment of sex chromosomes is so far based on the examination of a few species scattered across the phylogeny, and the observed patterns have rarely been assessed in a phylogenetic framework, making it difficult to infer rates of sex chromosome evolution and to distinguish between convergence versus common ancestry [but see (*19*)]. As of yet, no inclusive analysis of sex chromosome evolution exists for a cichlid adaptive radiation nor for radiations in other fish families.

**Fig. 1.**
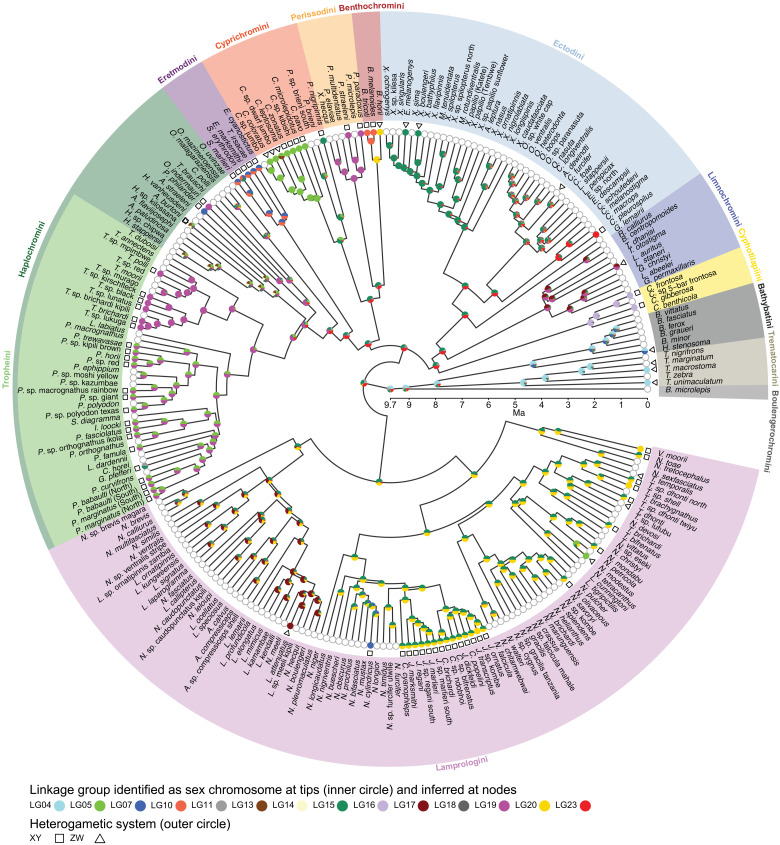
Sex chromosome evolution in the adaptive radiation of cichlid fishes in LT. Sex chromosome state and ancestral state reconstruction in LT cichlids (permissive dataset) are placed on a time-calibrated species tree (*17*), with background color shading indicating the 13 cichlid tribes of the LT radiation. The inner circle at tips shows identified sex-linked LGs. Colors refer to the LGs of the reference genome [23 LGs in the used reference genome represent the 22 chromosomes of *O. niloticus*, the most common chromosome number for African cichlids (*21*)]; two- or multi-colored symbols at tips indicate sex chromosomal signals that were detected on two or more reference LGs, suggesting chromosomal rearrangements between *O. niloticus* and LT cichlids. Symbols at the outer circle indicate the heterogametic SD system identified in each species (square, XY; triangle, ZW; no symbol, no system has been identified). Empty circles at tips indicate that within the dataset used here, no sex chromosome could be identified, nor has, to the best of our knowledge, genetic SD been investigated in these species elsewhere. Pie charts at nodes represent the probability for an LG being a sex chromosome at this time derived from ancestral state reconstructions.

In this study, we inspected genomic (*17*) and transcriptomic (*23*) information from 229 LT cichlid taxa and 19 cichlid species belonging to the Haplochromini and Lamprologini lineages phylogenetically nested within the LT radiation (*17*, *24*) for signatures of sex chromosomes. On the basis of this nearly complete taxon sampling of the LT radiation and an available time-calibrated phylogenetic hypothesis based on genome-wide data (*17*), we estimated the amount and direction of sex chromosome turnovers in this young species flock. This allowed us to test for a possible contribution of sexual antagonism in the evolution of sex chromosomes in LT cichlids. Sexual antagonism has been suggested as a driving force of sex chromosome turnovers in sexually dimorphic cichlids of the Lake Malawi radiation (*25*). However, unlike the cichlid adaptive radiation in Lake Malawi, which is composed solely of cichlids of the Haplochromini lineage, the LT cichlid assemblage consists of 16 cichlid lineages [corresponding to the taxonomic assignment into tribes, a taxonomic rank above the genus level (*26*)], some of which are sexually dimorphic while others are not. The taxa analyzed here belong to 13 of these monophyletic tribes (Fig. 1), as these form the actual adaptive radiation of cichlids in LT, while the sole representatives of the remaining tribes (Coptodonini, Oreochromini, and Tylochromini) colonized the lake secondarily (*17*).

To assess the dynamics of sex chromosome turnover in fishes on a larger scale, we expanded our comparative analyses to other fish systems as well. In particular, we investigated sex chromosome turnovers in ricefishes (genus *Oryzias*), a model system for the evolution of sex chromosomes (*27*).

Sex differences in the recombination rate (i.e., heterochiasmy) could contribute to the differentiation of sex chromosomes (*28*) and thus affect sex chromosome turnover rates. Unlike in the extremely heterochiasmic frogs of the family Ranidae (*15*) and some fish model organisms (*29*), recombination rates do not systematically nor drastically differ between the sexes in cichlids (*30*). In ricefishes, reduced rates of recombination have been linked to maleness in some species (*31*), yet this does not seem to be a general pattern in ricefishes (*32*). We thus hypothesize that ricefishes and cichlids have differing, probably lower, rates of sex chromosome degeneration than the heterochiasmic frogs. This, in turn, could affect sex chromosome and heterogamety turnover rates. In Ranidae, turnovers are driven by mutational load resulting from sex chromosome degeneration caused by immediate suppressed recombination. In general, we expect fewer, if any, cichlid species to be in the evolutionary trap of degenerated sex chromosomes and, in comparison to Ranidae, more sex chromosome turnovers caused by sexual antagonism than by mutational load (*10*). Last, with the identification of sex chromosomes in genetically very closely related species, we pave the way for the subsequent characterization of sex-determining genes and/or the causal mutations leading to sex chromosome turnover.

## RESULTS

### Sex chromosomes in LT cichlids

To identify sex chromosomes in LT cichlids, we screened male and female genomes of 244 taxa (typically one genome per sex per taxon; see Materials and Methods and table S1 for details) (*17*) as well as six multitissue transcriptomes from 66 taxa [three females and three males per taxon and combining three single-tissue transcriptomes per specimen into one (*23*)] for signatures of sex-linked regions, applying three complementary approaches: genome-wide association study (GWAS; on the genomic data, approach 1, see Materials and Methods), identification of sex-specific single-nucleotide polymorphisms (SNPs) in the genomic data (approach 2), and tests of allele frequency differences on the transcriptome data (approach 3). We applied approaches 1 and 2 on the tribe level, that is, we combined all genomes available for the representatives of a tribe into a single analysis, while approach 3 was performed on the species level.

The genomic locations of inferred sex-linked regions refer to the 23 linkage groups (LGs) of the version of the reference genome used in all analyses, the one of the phylogenetically equidistant outgroup to the cichlid species of the LT radiation, the Nile tilapia (*Oreochromis niloticus*). This species features the most frequent diploid number of 44 chromosomes of African cichlids (*21*). To estimate sex chromosome turnover rates, we used two different datasets; a “permissive dataset” including all sex chromosomes identified with approaches 1 to 3 and a “stringent dataset” excluding sex chromosomes that had support only in approach 2, i.e., without transcriptome data or lacking support for small sex-linked and potentially nonexpressed regions in the transcriptome data and/or occurring in tribes too small to be investigated with approach 1.

**Fig. 2.**
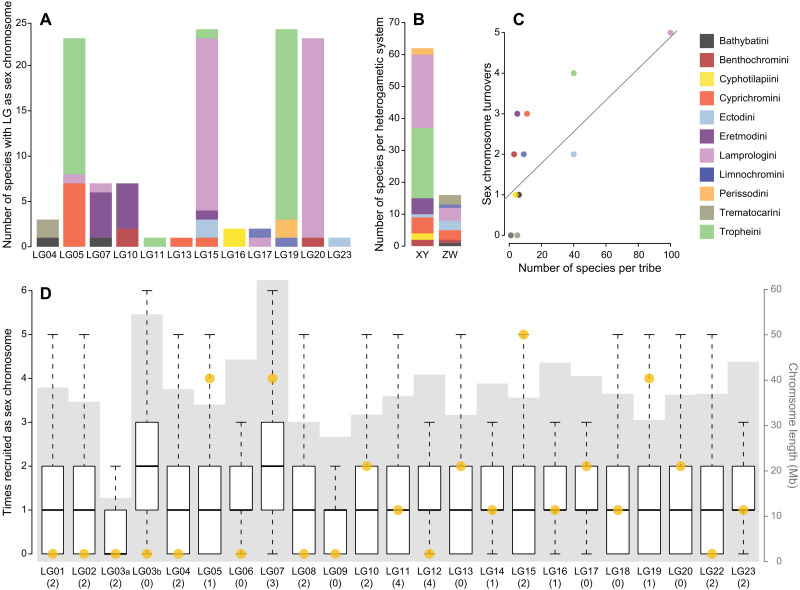
Nonrandom sex chromosome distribution in LT cichlids. (**A**) Use of different LGs as sex chromosomes. Bars represent the number of times a reference genome LG has been detected as sex-linked at the species level with colors referring to tribe (permissive dataset). (**B**) The occurrence of SD systems. Bars represent how often an XY or ZW heterogametic SD system was identified at the species level (permissive dataset) and with colors referring to tribe. (**C**) Association between species richness and sex chromosome turnover. The number of sex chromosome turnovers leading to the tips of each tribe (permissive dataset) is associated with the number of species investigated in each tribe (pGLS, *P* = 0.0043, coefficient = 0.039). Dots are colored according to tribes; the line represents the linear model fitted to the data. (**D**) Boxplots showing the expected number of sex chromosome recruitments if recruitment was at random (10,000 permutations). Boxplot centerlines represent the median, box limits the upper and lower quartiles, and whiskers the 1.5× interquartile range. Outliers are not shown. Ten reference LGs were never implicated in a turnover event in LT cichlids. Under random recruitment in the simulations, this pattern occurred only in 2.01% of all simulations. Yellow dots indicate the number of observed sex chromosome recruitments per reference genome LG derived from ancestral state reconstructions (permissive dataset), gray background shading represents chromosome length in megabases derived from the reference genome, and numbers below each boxplot indicate the number of previously described sex-determining genes on these reference genome LGs.

By combing the results of approaches 1 to 3, we detected signatures supportive of sex chromosomes in 78 endemic LT cichlid taxa and in the riverine haplochromine *Orthochromis indermauri* (Figs. 1 and 2, tables S1 to S3, and figs. S1 to S6). In the remaining 169 taxa, our approaches could not detect any sex linkage, suggesting the absence of sex chromosomes in these species, polygenic or polyfactorial SD systems, or a lack of resolution of the chosen approaches given the available data.

Approach 1 (GWAS), which we applied to the larger (that is, more species-rich) cichlid tribes from LT only, aimed at testing for the presence of sex chromosomes shared among several species of their respective tribes followed by the inspection of all individual genotypes in regions that showed sex association in the GWAS at the tribe level. We thus identified an XY heterogametic SD system on LG19 in Haplochromini/Tropheini [17 species; thereby confirming an XY system previously known from one species in this clade, *Tropheus* sp. “black” (*33*)]. We also uncovered an XY (four species) and a ZW (three species) heterogametic SD system on LG05 in different species of Cyprichromini, which correspond to two phylogenetic subgroups in this tribe, suggesting a single heterogamety transition [thereby confirming a ZW heterogametic SD system previously described in *Cyprichromis leptosoma* (*33*)]. In Lamprologini, we found an XY heterogametic system in a narrow region on LG15 and LG20 (23 species), suggestive of chromosomal rearrangements with respect to the reference genome (see below). We did not detect a (shared) sex chromosome with this approach within the second-most species-rich cichlid tribe of LT, Ectodini.

Approach 2, the inspection of the genomes within tribes for an accumulation of sex-specific SNPs (i.e., XY or ZW SNPs) and outlier regions thereof, disentangled two different XY heterogametic systems on LG19 within Haplochromini/Tropheini, one covering the first ~22 Mb of LG19 (in the genus *Tropheus* and in *O. indermauri*) and a second one located at the end of LG19 co-occurring with XY SNPs at the beginning of LG05 (in the second Tropheini clade grouping all genera but *Tropheus*, that is, 15 species belonging to the genera *Pseudosimochromis*, *Petrochromis*, and *Interochromis*; Fig. 1 and fig. S4). We also recovered the narrow sex-linked region on LG20 detected with GWAS in Lamprologini, corroborating the effectiveness of this approach. As in approach 1, we did not detect a sex-differentiated region shared across species in Ectodini.

When applied to the smaller tribes, approach 2 revealed rather narrow but clear outlier regions that were shared between subsets of species within Benthochromini (XY, LG10, two species), Trematocarini (ZW, LG04, two species), and Cyphotilapiini (XY, LG16, two species), as well as in all members of the Eretmodini (XY, LG07, and LG10). We also detected a less pronounced and smaller ZW outlier region on LG09 in the same two Cyphotilapiini species, a pattern potentially explained by variation in X-linked markers across the different species while simultaneously lacking homologous sites on the Y (hemizygosity in males). Because of this uncertainty and the stronger signal on LG16, we excluded the ZW signal on LG09 of Cyphotilapiini in the subsequent analysis (in the permissive and stringent datasets). Within Bathybatini and Perissodini, we identified a chromosome-wide increase of ZW SNPs on LG07 and of XY SNPs on LG19, respectively, which, however, failed our thresholds for the permissive dataset (see Materials and Methods). Upon inspection of XY-ZW differences per species within these tribes (fig. S5), this pattern turned out to be caused by only one species in each tribe (*Hemibates stenosoma* and *Plecodus paradoxus*, respectively), which both showed signs of a differentiated sex chromosome across the entire length of the respective LG. Note that a ZW heterogametic system on LG07 has previously been described for *H. stenosoma* (*33*), in line with the signal that we detected. We could also confirm, with approach 3 (see below), the XY heterogametic system spanning the full length of LG19 in *P. paradoxus* and in another Perissodini species, *Plecodus straeleni*, for which we did not have whole-genome data of both sexes. We hence included the sex chromosomes of these three species in all downstream analyses.

Approach 3, the species-specific investigations of sex-specific SNPs based on replicate transcriptome data, further confirmed all sex-differentiated regions shared among several species that spanned larger chromosomal regions, that is, the two XY SD systems on LG19 and LG05/LG19 in Haplochromini/Tropheini, the XY and ZW heterogametic SD systems on LG05 in Cyprichromini, and an XY heterogametic SD system in Eretmodini. The smaller sex-linked regions, such as the one on LG15/LG20 in Lamprologini, could not be confirmed with approach 3 (see Materials and Methods and table S2 for details).

With approach 3, however, we detected a ZW heterogametic SD system on LG15 in two Ectodini species (*Xenotilapia boulengeri* and *Enantiopus melanogenys*), not revealed on the tribe level by approaches 1 and 2. Approach 3 further permitted us to identify sex-linked LGs unique to eight additional species and not shared with their respective sister species (i.e., not detected with tribe-wise approaches). For example, we detected an XY heterogametic SD system on LG23 in the Ectodini species *Callochromis pleurospilus* and a ZW heterogametic SD system on LG20 in the Benthochromini species *Benthochromis horii* (Fig. 1 and table S2). In another four species, the RNA data showed an overrepresentation of either XY- or ZW-SNP windows, which, however, could not be attributed unambiguously on reference LGs (Fig. 1 and table S2).

Overall, in 9 of the 13 investigated tribes of the cichlid radiation in LT, several species shared the same SD system (chromosomal region and heterogametic type); however, we did not find a shared sex chromosome across members of different tribes.

We detected sex linkage on 12 of the 23 reference LGs (Fig. 2). Eight of these reference LGs were sex-linked in species belonging to different tribes (Fig. 2A). Two reference LGs (LG14 and LG18) that we did not identify as sex chromosomes within any of the endemic LT cichlid radiation species have respectively been identified as sex chromosomes in laboratory strains and a cross of the haplochromine cichlid *Astatotilapia burtoni* (occurring in LT and affluent rivers) (*34*, *35*). In addition to the published data for *A. burtoni*, we also included the previously published XY heterogametic LG07 sex chromosome of *Pseudocrenilabrus philander* (Lake Chila) (*19*) in our subsequent analyses; both haplochromine species, *A. burtoni* and *P. philander*, were included in the phylogenetic reconstruction used here (Fig. 1), but *P. philander* was represented by only a single individual in the genomic dataset and hence not accessible to our three approaches. *A. burtoni* was present in the whole-genome sequencing (WGS) dataset, albeit with individuals from different populations than those previously investigated for sex chromosomes (*34*, *35*). Note that these previously examined laboratory populations of *A. burtoni* differed with respect to their sex chromosome constellation and showed sex linkage on either LG18 or LG05/LG14 and/or LG13 (*34*, *35*). The *A. burtoni* individuals investigated here did not show signs of any of the two XY systems shared across the majority of Tropheini species. In 62 of the LT cichlids (79.5% of the LT species with a sex chromosomal signal), sex linkage was compatible with an XY heterogametic SD system, while the remaining 16 species had a ZW heterogametic SD system (Fig. 2B).

### Sex chromosome evolution in LT cichlids

Next, to determine when particular sex chromosomes evolved, and to trace heterogamety transitions over the course of the cichlid adaptive radiation in LT, we performed ancestral state reconstructions along a time-calibrated species tree (*17*). We performed these analyses on the permissive and on the stringent datasets.

We reconstructed 30 sex chromosome turnovers in the radiation and LG04 as the likely sex chromosome at its root (permissive dataset; 27 turnover events with the stringent dataset), translating into an estimated rate of 0.186 turnovers per million years (Fig. 1 and fig. S7, permissive dataset; turnover rate with the stringent dataset was 0.187 turnovers per million years). On average, we therefore expect one sex chromosome turnover event between two species that diverged ~2.7 million years ago. This rate estimate was 10 times higher than the one that we calculated for ricefishes (Adrianichthyidae; 0.02 transitions per million years; fig. S8 and table S4; 19 species investigated, see Materials and Methods).

The distribution of sex chromosomes in LT cichlids differed from random expectations (Fig. 2D). There was no association between the size of a reference LG, the number of genes on a reference LG, or the number of known SD candidate genes on a reference LG and the frequency at which these LGs became a sex chromosome in LT cichlids (Fig. 2D). Our findings thus corroborate that SD is a rapidly and nonrandomly evolving trait in cichlids. We further found that the number of turnovers in a tribe is associated with its species richness [phylogenetic generalized linear model (pGLS), *P* = 0.0043, coefficient = 0.039; Fig. 2C].

Our heterogamety reconstructions further suggested that XY male-heterogametic SD is the most likely ancestral state in the cichlid adaptive radiation in LT (fig. S9). Subsequently, 11 transitions occurred from XY to ZW heterogametic SD (permissive dataset; 11 toward ZW and 1 toward XY with the stringent dataset). Models suggest that transitions changing heterogamety involve new dominant mutations (*9*). This would predict that in cichlids from LT, just as in cichlids from Lake Malawi (*25*), new W chromosomes are dominant over ancestral Ys.

When integrating the reconstructed transitions in heterogamety and sex chromosomes, we found heterogamety changes that were uncoupled from turnovers in LGs and that were hence not captured in our rate estimate of sex chromosome turnover: We detected a transition from XY to ZW heterogametic SD on LG05 in Cyprichromini and on LG04 in Trematocarini and Bathybatini (*H. stenosoma*) (Fig. 1 and figs. S7 and S9).

The overlap of heterogametic and sex chromosome turnovers also showed that most (23 versus 7) of the observed sex chromosome turnovers in LT cichlids preserved the heterogametic system, suggesting that mutational load, predicted to maintain the heterogametic state (*15*), might be a major driver of sex chromosome turnover in cichlids as well. The transitions with a change in heterogamety offer the possibility to investigate the actual potential of sexual antagonistic selection between very young species (the divergence time between, e.g., *Paracyprichromis* and *Cyprichromis*, between which a turnover has occurred, is ~3.8 million years). The heterogametic status of the four species for which we could not identify the sex-linked LG (see above) led to additional heterogamety transitions not reflected in the estimated sex chromosome turnover rate.

Overall, the heterogamety transition rate in LT cichlids (0.028 transitions per million years with the permissive dataset; 0.031 per million years with the stringent dataset) was about four times higher than in ricefishes (0.007 transitions per million years; ancestral state ZW). To explore heterogamety changes on a greater taxonomic scale, we also calculated heterogamety transition rates for all ray-finned fishes included in both the Tree of Sex database (http://treeofsex.org/) and a recent comprehensive fish phylogeny (*36*) (543 species analyzed in total). Our analysis estimated a rate of 0.009 transitions per million years for ray-finned fishes as a whole and identified XY as the ancestral state (table S5 and fig. S8). Across the ray-finned fish phylogeny, transitions from XY to ZW were significantly younger than those from ZW to XY (*P* = 0.01428; fig. S8B).

### Chromosome fusions and novel sex chromosomes

Novel sex chromosomes can arise by chromosome fusions (*37*), which can contribute to reproductive isolation and eventually drive speciation over mis-segregation at meiosis, changes in recombination rates, novel physical combinations of loci, and/or changes in gene expression (*38*–*40*). The here identified signatures of sex linkage distributed on two (or more) reference LGs suggest chromosomal rearrangements in the species investigated compared to the used reference genome that has the most common African cichlid karyotype (2*n* = 44) (*21*). This could suggest that several sex-chromosome/autosome fusions have occurred over the course of the cichlid radiation in LT or that autosome/autosome fusions occurred before the recruitment of the then fused autosomes as sex chromosome (Fig. 1). The distribution of sex-differentiated genomic regions on different reference LGs suggests a fusion or large chromosomal translocations between LG05 and LG19 in Haplochromini/Tropheini and between LG15 and LG20 in Lamprologini (Fig. 1 and fig. S1). There was also some support for the previously described genome rearrangements in the tribe Eretmodini (*21*), which showed an increase of XY SNPs on several LGs (Fig. 1 and fig. S3). Additional sex-differentiated regions could support species-specific fusion events (e.g., LG11 and LG15 in *Gnathochromis pfefferi*). Our analyses also confirmed the reported sex linkage of LG04 and of LG07 in *H. stenosoma* (fig. S5) (*33*). Chromosome fusions have previously been implicated with the evolution of novel sex chromosomes in other taxa, also including the haplochromine cichlid *A. burtoni* (*34*, *35*).

The so far only karyotypically investigated member of the tribe Tropheini, *Ctenochromis horei*, has a reduced number of chromosomes in a male and an unsexed individual (2*n* = 40) compared to other Haplochromini, which usually feature 2*n* = 42 (*21*). We did not detect the LG05/LG19 XY heterogametic SD system found in many other Tropheini in *C. horei*. Hence, while the karyotype of this species supports chromosomal fusions in the Haplochromini/Tropheini, these data cannot help to resolve when and how structural genome changes occurred. The data at hand are sparse, but it might be that several large chromosomal rearrangements occurred after the split from *O. niloticus* and before the novel chromosomes were recruited as sex chromosomes, asking for further investigations of the driving forces of these potential rearrangements.

### Convergent evolution of sex chromosomes

**Fig. 3.**
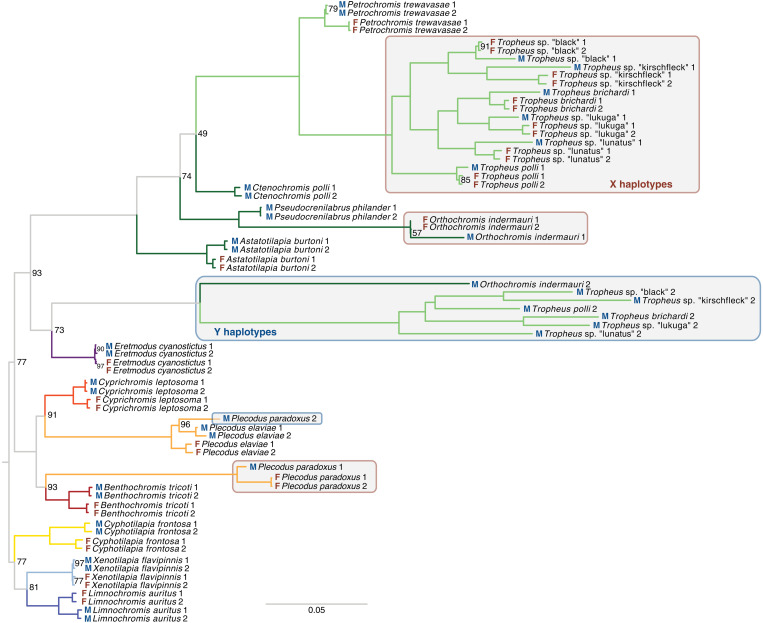
Convergent evolution of LG19 as XY sex chromosome in two Tanganyikan cichlid tribes. The phylogenetic tree of X and Y haplotype sequences does not group the *P. paradoxus* Y haplotype with the *Tropheus* Y haplotypes but supports the species tree, suggesting convergent evolution. The Y haplotype of the non-LT riverine haplochromine *O. indermauri* groups with the Y haplotypes of the *Tropheus* species, supporting monophyly of this sex chromosomal system. The scale bar indicates the number of substitutions per site; values at nodes represent bootstrap support (% of 1000 bootstraps, if no value is shown the node support was 100%).

On some LGs, the regions that showed sex linkage were largely the same between members of different tribes (fig. S10), which can be explained by either common ancestry or by the independent (convergent) recruitment of those LGs as sex chromosome. In particular on LG19, several closely related species including six *Tropheus* species (Haplochromini/Tropheini), the riverine haplochromine *O. indermauri*, and the Perissodini species *P. paradoxus* and *P. straeleni* feature an XY SD system in the same chromosomal region (fig. S10). Our ancestral state reconstruction suggested the independent origin of the LG19 male heterogametic SD system in Perissodini and *Tropheus*, in each case early in their tribe’s evolutionary history, and another independent origin on the terminal branch leading to *O. indermauri* (fig. S7). Phylogenetic inference from Y and X haplotypes further supported the independent evolution of LG19 as XY sex chromosome in Perissodini (Fig. 3), while grouping together the Y haplotypes of the *Tropheus* species and *O. indermauri*. This suggests common ancestry of the XY heterogametic SD system in the two haplochromine clades, with an origin either early on in haplochromines (implying several losses later in the evolution of this tribe; likely because of this, such a scenario was not supported by ancestral state reconstruction) or a later origin and inheritance of the sex chromosomal system in *Tropheus* and *O. indermauri* from an extinct or unsampled taxon.

The XY sex chromosome system on LG05/LG19 found in the second clade of Haplochromini/Tropheini (grouping all genera except *Tropheus*) must be derived from another independent evolutionary event, because the regions on LG19 that show XY alleles in the two Haplochromini/Tropheini clades are not overlapping (fig. S10) and also do not group together in the phylogenetic tree of LG19 haplotypes (Fig. 3). Other convergent cases of sex chromosome recruitment supported by our ancestral state reconstruction involved LG05 [in Cyprichromini and the haplochromine *A. burtoni* (*34*, *35*)] and LG07. LG07 has independently been recruited as a sex chromosome in *H. stenosoma* (Bathybatini) (*33*), in Eretmodini, in the lamprologine *Neolamprologus cylindricus* (Fig. 1 and fig. S7), in several Lake Malawi cichlids (Haplochromini) (*25*, *41*), and in *P. philander* (Haplochromini) (*19*), making it the most widespread sex-linked LG known in cichlids to date.

### Sex chromosome differentiation

**Fig. 4.**
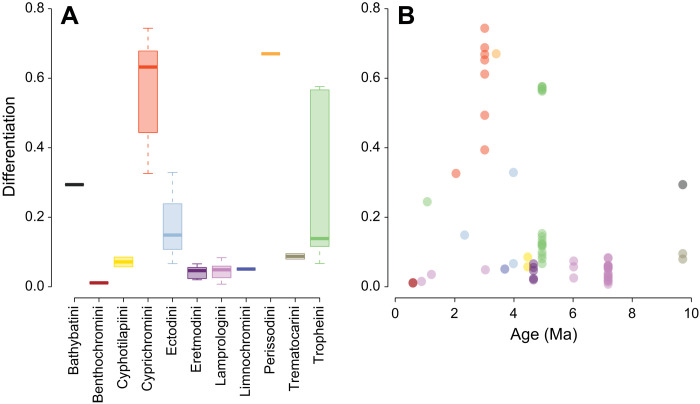
Sex chromosome differentiation in LT cichlids. (**A**) Size distribution of sex-differentiated regions. The size of these regions corresponds to the proportion of the reference genome LG with windows that have more sex-specific SNPs than two times the mean across all windows. (**B**) Per-species proportion of the chromosome(s) showing sex differentiation and corresponding estimated ages of the sex chromosomal system based on ancestral state reconstructions on a time-calibrated species tree. The degree of differentiation is not associated with the estimated age of origin (pGLS, *P* = 0.9049, coefficient = 0.0011).

A comparison of the proportion of sex-specific sites on the different sex-linked LGs revealed a continuum of sex chromosome differentiation in the cichlid adaptive radiation in LT (Fig. 4 and fig. S10), ranging from a few kilobases (LG20 in Lamprologini) to almost full chromosomal length (LG05 in Cyprichromini and LG19 in *Tropheus* and Perissodini). We even detected varying lengths of sex-differentiated regions within the same LG when being used as sex chromosome by species of different tribes (e.g., the sex-differentiated region on LG05 spans only 8 Mb in Tropheini but the entire LG in Cyprichromini).

The canonical model of sex chromosome evolution predicts progressing differentiation of sex chromosomes with time (*2*). Contrastingly, we found no association between the estimated age of origin of a sex chromosome and its degree of differentiation (pGLS, *P* = 0.9049, coefficient = 0.0011; Fig. 4). Some very young sex chromosomes showed signs of differentiation, i.e., sex-specific sites, along almost the full length of an LG, suggesting widespread suppression of recombination along these sex chromosomes.

### Candidate genes of SD in LT cichlids

Our inspection of known genes implicated in SD revealed that such genes were located on all LGs, including those that did not show any sex linkage, with no particular overrepresentation on certain LGs (fig. S11). The regions with the strongest signal for being sex-differentiated did not contain any of these genes (table S2). However, through the inspections of the regions with the strongest signs of sex linkage, we identified promising candidate genes for SD in these regions, such as *tox2* in Lamprologini, an HMG (high mobility group)-box transcription factor involved in the hypothalamic-pituitary-gonadal system. *Tox2*, just like the mammalian master SD gene *Sry*, codes for an HMG-box protein and is involved as a transcriptional activator in the hypothalamo-pituitary-gonadal system.

In cichlids from Lake Malawi and Lake Victoria (*25*, *42*), sexually antagonistic color genes underlying a characteristic orange-blotched color pattern are linked to SD genes, creating the potential for speciation by sexual selection. In LT cichlids, which in general do not feature the orange-blotched phenotypes, we did not find any obvious pattern in the localization of color genes on sex-linked LGs (fig. S11).

## DISCUSSION

Here, we report the identification of genomic signatures supportive of sex chromosomes in 79 taxa of cichlid fishes, most of which belonging to the cichlid adaptive radiation of LT, based on the analysis of whole-genome data from virtually all cichlid species of the radiation (*17*) and transcriptome data from a representative set of 66 taxa (*23*). Models (*9*) and empirical observations (*13*) suggest that beyond a certain degree of differentiation, sex chromosome turnover becomes unlikely. On the other hand, frequent turnovers, sex reversal, and continued recombination can contribute to counteract sex chromosome differentiation (*43*). Our analyses revealed that in the cichlid adaptive radiation of LT, sex chromosome turnovers seem to have occurred very frequently (Fig. 1), indicating that the cichlids’ sex chromosomes have not (yet) reached a threshold preventing turnover, but that their sex chromosomes remain dynamic instead.

**Fig. 5.**
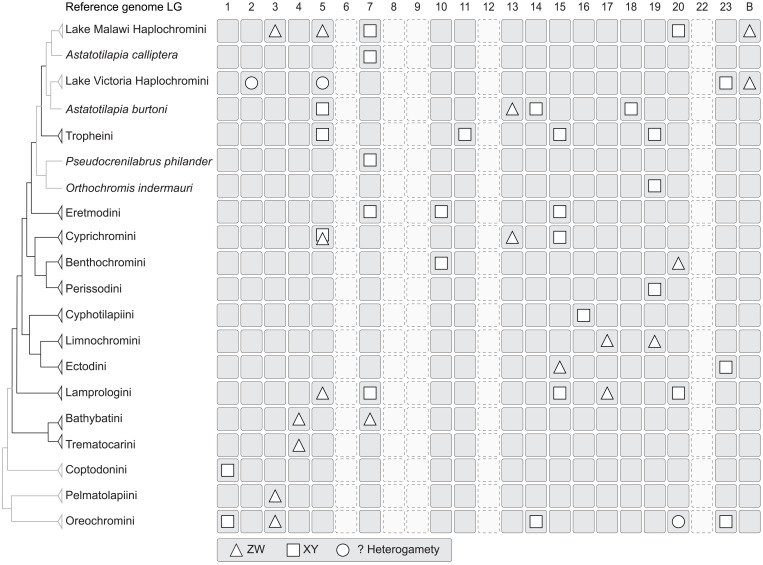
Sex chromosome evolution in African cichlids. Phylogenetic relationships in African cichlids are based on previous studies (*17*, *19*). Sex chromosome occurrences are denoted with reference to the 22 chromosomes of the genome of the Nile tilapia (*O. niloticus*, tribe Oreochromini). Note that the naming of the chromosomes relates to previous naming of LGs and is missing “21” because LG21 became part of LG16 in the course of establishing chromosome length genome assemblies. “B” refers to B chromosomes, i.e., supernumerary chromosomes found in some cichlid species. Nile tilapia strains exist with an XY heterogametic system on LG1 and an XY heterogametic system with a Y-specific *amh* SD gene on LG23, respectively (*52*)). Cichlid lineages of LT are indicated with black branches, cichlids from other lakes or rivers with gray branches. Sex chromosome information is derived from this study and previously published summaries (*19*, *20*) that are also based on the 22 *O. niloticus* chromosomes as the most common African cichlid karyotype (*21*).

As far as we could identify it, sex chromosome recruitment in LT cichlids is nonrandom with respect to the recruited chromosome (Fig. 2). This pattern becomes even more apparent when the LT cichlids are compared to other African cichlid species (Fig. 5), revealing that some LGs (in particular LG05, LG07, and LG19) emerged multiple times as sex chromosomes, whereas other LGs never appeared as such. This corroborates the “limited options” hypothesis in the sense that particular chromosomes are preferentially (*22*) or even cyclically (*43*) recruited as sex chromosomes, probably because of genes that are readily suited as master sex determiners on the basis of their previous implication in the SD gene network. As of now, we lack information on the genes that could drive this pattern in the cichlid radiation of LT and in cichlids in general. With *wnt4*, LG05 contains a prime candidate for a master SD gene in cichlids (*34*, *35*), yet a major sex-determining role of this gene lacks functional evidence. Further potential candidates with a function or a gene ontology related to SD/sex differentiation, located on the three LGs in question, are *rbbp5*, *prkag1*, *cdk2*, and *fkbp11* on LG05; *fkbp4*, *ash2l*, *lman1*, and *hsd17b12* on LG07; and *slirp*, *esr2*, and *clic4* on LG19.

Within LT cichlids, sex chromosome turnovers have likely been driven by a combination of mutational load and sexual antagonism. However, we detected a prevailing persistence of male-heterogametic SD in LT cichlids, which is a common pattern in fishes (*44*), suggesting a smaller role for sexual antagonism than previously thought. Furthermore, the observed prevalence of XY systems is compatible with models of speciation driven by sexual selection and sex ratio distortion in cichlids that predict higher probabilities for the maintenance of male-heterogametic SD systems (*45*).

The evolution of a novel sex determiner driven by linkage to a sexually antagonistic color locus has previously been documented in haplochromine cichlids from Lake Malawi (*25*), which are characterized by pronounced levels of sexual dimorphism. In our set of mostly riverine Haplochromini and Tropheini species (the LT endemic representatives of this clade), we found that a single sex chromosome system prevails, XY heterogametic on LG05/LG19, which was probably established after a turnover from the rather strongly differentiated XY heterogametic LG19 SD system present in the genus *Tropheus*. It thus appears that in the Tropheini, in which sexual dimorphism is much less pronounced (and even absent in some species) compared to the radiations of Haplochromini in lakes Malawi and Victoria, sexual antagonism does not play a prominent role as a driving force for sex chromosome turnover. Still, several Tropheini species seemingly have lost the XY LG05/LG19 SD system. We were, however, mostly unable to detect a new system that replaced it based on the available transcriptome data, probably because the sex-linked chromosomal regions are rather small. These species will be particularly interesting to investigate further for the presence and the drivers of very young, novel sex chromosomes with a potential role for sexual antagonism affecting sex chromosome turnover (*46*). In addition, the observed cases of young homologous sex chromosome turnovers between closely related species (e.g., in the genus *Cyprichromis* on LG05 or in Trematocarini on LG04), which are indeed compatible with a role for sexual antagonism as a driving force in cichlid sex chromosome evolution (*6*), open the route for further analysis of the causal mutations driving sex chromosome turnovers. In particular, the presence of several ZW and XY species in Cyprichromini, reconstructed to be caused by a single XY-to-ZW heterogamety transition event on the same chromosome, will allow tracing which alleles have been affected by a heterogamety change in the future. These analyses may eventually reveal the causal mutation(s) (supposedly within the SD gene) of the heterogamety turnover and the dominance relationships between XY and ZW systems.

While the data at hand support the presence of sex chromosomes in 79 taxa, we failed to detect signatures of sex linkage in the remaining species of the LT radiation, leaving them with no sex chromosome assignment as of yet. This can, to some extent, be explained by our limited sample size per species, the lack of sex chromosomes shared between several species in some tribes/genera reducing the power of approaches 1 and 2, the lack of strongly differentiated sex chromosomes, and/or the limited power to detect small sex-specific regions, especially when using transcriptome data, as well as in complex polygenic SD systems. Thus, it remains to be determined whether sex chromosomes, or more generally, genetic SD, exist in these taxa. We also acknowledge that the limited sample size per species in the data currently available to us may have left species-specific SD regions undetected.

We nevertheless found it intriguing that we could only identify sex-linked regions in three species across the second most species-rich tribe, Ectodini [note that the available transcriptome data are representative in terms of species-richness per tribe (*23*)]. Some species of this tribe display an impressive level of sexual dimorphism, suggesting similar or even more pronounced sexual antagonistic selection compared to tribes such as the Haplochromini/Tropheini, which show relatively strongly differentiated sex chromosomes. It will thus be interesting to examine whether Ectodini, but also members of other LT cichlid tribes, have species-specific and/or very small, if any, sex-linked genome regions that our approaches on the tribe level and using transcriptomes failed to detect. This would further reveal whether selective forces on SD differ within the radiation and whether our assessment of the sex chromosome turnover rate is underestimating the true dynamics of sex chromosome change in LT cichlids.

The presence of sex chromosomes in many (LT) cichlid species remains to be assessed, with our analysis being a first step toward this direction. Still, our ancestral state reconstructions based on a comprehensive sampling of the entire LT radiation estimated a sex chromosome turnover rate in LT cichlids that is 10 times higher than the one in ricefishes, another group of fishes with an astonishing diversity of sex chromosomes. The turnover rate estimated for LT cichlids is also higher than the one published for true frogs, which was previously considered the fastest sex chromosome turnover rate known in vertebrates (*15*). Note that extremely high numbers of SD system turnovers have also been described in geckos (*16*), but these have so far not been used to calculate a comparable rate estimate.

Chromosome fusions could drive speciation through incompatibilities in genome structure (*38*, *39*), and cytogenetic analyses have indeed provided evidence for chromosome fusion and fissions in some cichlid species (*21*). However, their impact on cichlid diversification has not yet been assessed. Sex chromosome/autosome fusions generating an odd number of chromosomes in one sex and leading to the formation of neo-sex chromosomes can be driven by altering expression of genes on the translocated chromosome (*47*), sexually antagonistic selection resolving conflict by restricting an antagonistic allele to a sex chromosome (*48*), or meiotic drive (*49*). Until now, differences in chromosome number between male and female cichlids have not been reported, with the notable exception of copy number variations in female-determining B chromosomes in Lake Victoria and Lake Malawi cichlids (*50*, *51*). For the limited number of cytogenetically investigated LT cichlid species, males and females have the same number of regular chromosomes, and across African cichlids in general, chromosome numbers differ very little (*21*). Overall, our analyses revealing sex linkage on two (or more) reference LGs within LT species provide support for several large chromosomal rearrangements between the identified sex-linked LGs and the reference genome, suggesting that structural changes in the genome and the emergence of sex chromosomes are coupled in cichlids. The timing, and thus, the causality of this relationship remain to be investigated, just as the impact of genome rearrangements on reproductive isolation and eventually diversification in cichlids. The available data on genome rearrangements are sparse, but it might be that several large chromosomal rearrangements occurred in (LT) cichlids before these novel chromosomes were recruited as sex chromosomes, making inferences of the driving forces of these fusions worth investigating in more detail.

A next, necessary step will be the identification of sex-determining genes and mutations causing sex chromosome turnover. This is facilitated by the close relatedness of LT cichlids allowing the generation of interspecies hybrids and also through the opportunity to study multiple sex chromosome turnover events and directions, including the repetitive occurrence of heterogamety transitions without sex chromosome change. While the repeated recruitment of the same LG as sex chromosome indicates a particularly well-suited core set of SD genes on the one hand, several transitions to otherwise not recruited LGs on the other hand question their supremacy. Although this could represent recycling of sex chromosomes to some extent, we lack the molecular and, more importantly, functional evidence for any master SD gene in cichlids of LT or any other cichlid radiation. As of yet, only a single master SD gene, *amh*, has been characterized in cichlids in the Nile tilapia *O. niloticus* (*52*). While *amh* is indeed a usual suspect among vertebrate SD genes and has repeatedly been recruited as master SD gene in different species, its SD function is not conserved across different *O. niloticus* strains (*53*). Thus, the identity of SD genes, and whether they largely derive from a small set of genes known to be implicated in SD in other lineages, remains to be characterized in (LT) cichlids.

In conclusion, the estimated rapidity of sex chromosome turnover within (LT) cichlids supports the hypothesis that SD mechanisms, albeit sharing the same function of SD, can be extremely labile. It remains to be tested whether sex chromosome turnovers are so frequent as a side effect of a generally rapid evolution of cichlid fishes (the number of turnover events is associated with species richness within tribes) or whether they even drive this rapidity, potentially contributing to speciation.

## MATERIALS AND METHODS

### Experimental design

In this study, we investigated available genomic (*17*) and transcriptomic (*23*) datasets (i) to identify and characterize sex chromosomes in species covering the entire LT cichlid radiation, (ii) to trace the evolutionary history of sex chromosomes within the radiation to shed light on the dynamics of sex chromosome turnover in a rapidly diversifying lineage, and (iii) to embed our results in a broader context by comparing estimates of turnover rates and potential drivers of sex chromosome evolution to other taxa.

### Sequencing data

We used WGS data in the form of mapped reads in BAM files and in the variant call format from Ronco *et al.* (*17*) and raw transcriptome data from El Taher *et al.* (*23*) (see table S1 for details on species included and per-species sample sizes). On the basis of a recent compilation of LT cichlid species (*26*), the WGS data included 225 taxa (174 described species, with 4 of those represented with two local variants/populations each, and 47 undescribed species). The data further included 16 non-LT radiation haplochromine cichlid taxa (13 described species, 1 of which represented with two local variants, and 2 undescribed species) and 3 riverine non-LT Lamprologini taxa (2 described and 1 undescribed species) summing to a total of 244 taxa and 469 individual genomes, typically in the form of one female and one male genome per taxon (table S1). The transcriptome data were composed of 66 taxa of LT cichlids (4 undescribed species and 61 described species, 1 of which represented with two regional variants), with typically three males and three females per species (7 of the 66 species had differing replicate numbers; details are provided in table S1) and three individually sequenced tissues per specimen (brain, gonads and gills; details on read numbers provided in table S3) that we pooled into one transcriptome per specimen, resulting in typically six transcriptomes per species. In total, the dataset comprised 248 cichlid taxa.

### Variant calling for WGS data

We derived mapped reads in coordinate-sorted BAM format from Ronco *et al.* (*17*) [for mapping coverage statistics, see supplementary table 1 in the study by Ronco *et al.* (*17*)], which are based on mapping against the Nile tilapia (*O. niloticus*) genome [National Center for Biotechnology Information (NCBI) RefSeq GCF_001858045.1_ASM185804v2]. This reference genome was sequenced from a homozygous clonal XX female with LG1 being the sex chromosome and was chosen in the original study (*17*) and here because it represented, at the time of mapping, the most complete and contiguous cichlid genome assembled to the chromosome level. Furthermore, the Nile tilapia is a phylogenetically equidistant outgroup to all LT cichlids, which minimizes mapping bias. The Nile tilapia also shows the most common African cichlid karyotype with a diploid chromosome number of 44 (*21*). We concatenated unplaced scaffolds of the reference genome lexicographically into an “UNPLACED” super chromosome.

In addition to the variant file containing all species derived from Ronco *et al.* (*17*), we called variants for each tribe separately with GATK’s (v.3.7) HaplotypeCaller (per individual and per chromosome) and GenotypeGVCFs (per 1-Mb window), and merged them with GATK’s CatVariants. We further filtered variants with BCFtools (v.1.6, http://samtools.github.io/bcftools/), applying the settings ReadPosRankSum < −0.5, MQRankSum < −0.5, FS < 20.0, QD > 2.0, MQ > 20.0, and placing tribe-specific thresholds on minimum and maximum read depths to account for varying sample sizes (Bathybatini, 50 to 300; Benthochromini, 25 to 100; Cyphotilapiini, 50 to 200; Cyprichromini, 100 to 400; Ectodini, 250 to 1500; Eretmodini, 50 to 200; Tropheini/Haplochromini, 375 to 1375; Lamprologini, 700 to 3000; Limnochromini, 50 to 300; Trematocarini, 50 to 300). For the tribes Lamprologini, Tropheini/Haplochromini, Ectodini, and Limnochromini, we further applied InbreedingCoeff > −0.8.

We normalized indels with BCFtools’s norm function, excluded monomorphic sites, and masked SNPs around indels depending on the size of the indel: For indels with a size of 1 base pair (bp), 2 bp were masked on both sides, and 3, 5, and 10 bp were masked for indels with sizes of 3 bp, 4 to 5 bp, and >5 bp, respectively. We then masked individual genotypes with VCFtools (v.0.1.14) (*54*) if they had low quality (--minGQ 20) or depth (--minDP 4). Filtered variants were phased, and missing genotypes were imputed with Beagle (v.4.1) (*55*). We then retained only biallelic sites that had no more than 50% missing data before phasing. For sites that were polymorphic but no individual had the reference genome allele, we set the first alternative allele as reference allele.

### Approach 1 tribe-wise association tests for sex on WGS data using GWAS

In total, we used three approaches to identify signatures supportive of sex-linked genomic regions (approach 1 to 3). We performed approaches 1 and 2 at the tribe level, the taxonomic rank above genus but below family, which, in the case of the LT radiation, groups monophyletic clades that are between 6.2 and 9.7 million years old. Note that LT cichlid genera in contrast to tribes are not always monophyletic. The tribes comprise between 1 (Boulengerochromini) and ~100 (Lamprologini) species. Approaches 1 and 2 have been designed to detect signatures of sex chromosomes shared across species within tribes, which we presumed to likely exist because of the close relatedness of the species.

For approach 1 (figs. S1 and S2), the phased sets of variants for tribes with at least 10 species (Lamprologini, sample size of 196 individuals representing 100 species; Ectodini, sample size of 81 individuals representing 40 species; Haplochromini including the LT-endemic Tropheini, sample size of 99 individuals of 55 species; and Cyprichromini, sample size of 21 individuals of 11 species) were each transformed into bim and bed format with PLINK (v.1.90b) (*56*). Next, we ran association tests (GWAS) for sex on these tribe-specific variant files using the univariate linear mixed model integrated in GEMMA (v.0.97) (*57*), accounting for population stratification. After visual inspection of GWAS results for potentially sex-associated regions on the tribe level (i.e., peaks or shifts of increased significance), genotypes of the 100 most significantly sex-associated SNPs for Haplochromini and Cyprichromini (broad signal for sex association along the entire length of LG19 and LG05, respectively) and of outlier SNPs [narrow peak regions on LG15, LG20, and unplaced contigs comprising 51 SNPs with a −log10(*P* value) > 3; extraction of the top 100 most significantly sex-associated SNPs revealed same clustering and no further sex-associated region because those SNPs were scattered across the genome] of all individuals analyzed by GWAS were clustered and visualized with the R package Pheatmap (v.1.0.12, https://cran.r-project.org/web/packages/pheatmap/index.html) in R (v.3.5.2). We inspected these genotype clusterings further for grouping by sex to (i) infer which species drove the global pattern observed in the GWAS and (ii) with which heterogametic SD system.

### Approach 2 tribe-wise tests for an accumulation of sex-specific SNPs

We applied approach 2 to tribes that contain more than a single species (table S1 for all sample sizes), again under the assumption that closely related species of the same lineage might share a sex chromosome: We here tested for an accumulation of sites with sex-specific alleles, referred to as XY and ZW sites depending on the heterogametic sex (figs. S3 and S4), under the assumption that a sex chromosomal region will show an accumulation of sex-specific alleles due to linkage caused by suppressed recombination. To this end, we subset the unphased, filtered sets of variants per tribe and included only species for which we had individuals of both sexes (table S1). We then removed indels and sites with more than 20% missing data and more than two alleles with VCFtools (v.0.1.14). We loaded the resulting files into R (v.3.5.0) with VCFR (v.1.8.0.9) (*58*) and classified sex-specific sites as follows: Each variant site was recoded per species as a “nosex” site if the male and the female individual had the same genotype, as “noinfo” if one or both individuals had no genotype call, as “XY” if the male was heterozygous and the female homozygous, and as “ZW” if the female was heterozygous and the male homozygous. Next, we calculated, within each tribe, the sum of nosex, ZW, and XY sites in windows of 10 kb with a slide of 2 kb as well as the difference between XY and ZW sites per window. Next, we calculated the mean genome wide percentage of nosex, ZW, and XY sites over all windows and multiplied these values with the number of called sites per window to obtain expected values for XY, ZW, and nosex under the assumption that most variant sites across the genome show no particular sex difference. The expected values per window were compared to the observed values using a Fisher’s exact test, with the exception of the Lamprologini in which the large counts of sites per window rendered exact calculations with a Fisher’s exact test impossible so that we applied a Pearson’s χ^2^ test. These tests indicate windows that significantly differ from the genome-wide mean. Next, we designated and plotted a window with its corresponding *P* value as (i) XY if the observed XY value was greater than the expected one and the observed ZW value smaller than the expected one and as (ii) ZW if the observed ZW value was greater than the expected one and the observed XY value smaller than the expected one. If both the observed XY and ZW values were larger than the expected value, then a window was declared ambiguous and not further considered. If both observed XY and observed ZW values were equal or smaller than the expected values, then a window was declared nosex and not considered further. Fisher’s exact test and Pearson’s χ^2^ *P* values of XY and ZW windows were plotted jointly (on −log_10_ and log_10_ scales, respectively) and with an overlay of the calculated XY-ZW difference for each window normalized by dividing the obtained value through the number of species analyzed. We inspected the obtained plots for the presence of LG-wide or regional shifts in XY-ZW difference and outliers from the expected XY or ZW sites. We also calculated and visualized the XY-ZW difference in each window at the species level. To assess a false discovery threshold, we permutated the observed data within each tribe 100 times by randomly assigning the SNPs to the observed genomic positions. We recalculated the XY-ZW difference per window and the expected values. We assessed, from each permutation, the highest absolute XY-ZW difference of a window and the smallest *P* value for XY/ZW sites. The largest absolute XY-ZW difference normalized by species number across all permutations within each tribe was then used as minimal threshold to define the sex-linked regions in the observed data. The lowest *P* value of all XY/ZW windows across all permutations was −log_10_(*P* value) = 5.04 (obtained in the tribe Haplochromini/Tropheini), which corresponds to a false discovery rate (FDR) ~ 4 after Bonferroni correction. To minimize the possibility of false positives after a comparison of all observed data across all tribes, we lastly retained only drastic XY/ZW outlier regions that, in addition of exceeding the tribe-wise threshold of XY-ZW difference derived from each tribe’s permutation, also had a −log_10_(*P* value) > 20 (corresponding to a FDR of 2.30 × 10^−26^ after Bonferroni correction).

Upon a first inspection of sequence content of sex-linked regions, we noticed in the empirical RNA and DNA data XY and ZW peaks in different tribes/species within the same region on LG02 of the reference genome. This region (21.36 to 21.93 Mb) is annotated with 26 protocadherin tandem gene copies. We suspect that this array of similar genes affects mapping and hence masked this region from our sex chromosomal call. Furthermore, it has previously been shown that LG03 is a sex chromosome in *Oreochromis* spp. and that the assembly quality of this region is poor because of presence of repetitive elements, leading to difficulties in the identification of sex-linked regions on this LG (*59*). This is also reflected in our data by an excess of missing data on this LG and, hence, less reliable SNP data. We therefore also excluded outlier regions on LG03 as potential sex chromosome.

Because we applied approach 2 on the tribe level, we next needed to identify how many and which species were responsible for the sex chromosome signals detected within a tribe, i.e., identify the sex chromosomes on the species level from this approach. To this aim, we visualized, per window and for all specimens analyzed by this approach, species level XY-ZW differences in the outlier regions and clustered individual genotypes (with the possible values “homozygous reference,” “homozygous alternative,” or “heterozygous”) with divisive hierarchical clustering based on a pairwise dissimilarities matrix of Gower’s distances calculated with the R package FSA (v.0.8.30) (https://github.com/droglenc/FSA). We inspected resulting dendrograms for grouping by sex rather than species and the boxplots of species-specific XY-ZW difference for support by increased absolute XY-ZW difference. Because of the reduced sample size per species and to avoid false-positive signals, we made final calls for sex linkage on LGs and heterogametic status based on the outlier regions only if at least two species within a tribe shared the same signature of sex linkage (table S2).

### Approach 3 species-specific association tests for sex on transcriptome data

For approach 3, the identification of sex-linked regions and heterogametic system on the species level by species-specific association tests (fig. S6), we pooled tissue-specific transcriptomes of brain, gonad, and gills into one transcriptome per individual and quality-filtered and trimmed these with Trimmomatic (v.0.33) (*60*) with a 4-bp window size, a required window quality of 15, and a minimum read length of 30 bp, resulting in typically six multitissue transcriptomes per species (table S3 for read numbers). We then ran the following analysis for each species: we performed reference-free de novo variant calling with KisSplice (v.2.4.0) (*61*) with the settings “-s 1 -t 4 -u” and “--experimental.” We placed the identified SNPs on the Nile tilapia genome assembly with STAR (v.2.5.2a) (*62*) with the settings “--outFilterMultimapxNmax 1,” “--outFilterMatchNminOverLread 0.4,” and “--outFilterScoreMinOverLread 0.4.” The genome index used for mapping was generated with the corresponding STAR parameters “--runMode genomeGenerate,” “--sjdbOverhang 124,” “--sjdbGTFfeatureExon exon,” and the genome annotation file (RefSeq GCF_001858045.1_ASM185804v2). We used Kiss2Reference (*61*) to classify KisSplice variants aligned to the Nile tilapia reference genome and applied kissDE (v.1.4.0) (*61*) to determine variants that differed between the two sexes. We loaded the resulting files into R and filtered the KisSplice events with the following attributes: We kept only SNPs, removed SNPs placed on mitochondrial DNA or on unplaced scaffolds of the reference genome, and retained only SNPs with significant *P* values for an allele difference between the sexes (*P* ≤ 0.05 after adjustment for multiple testing following the Benjamini and Hochberg method). We classified SNPs as (i) XY if they had zero read counts in all females and a minimum of one count in at least two males and as (ii) ZW if they had zero counts in all males and a minimum of one count in at least two females. Next, we assessed the density of these XY and ZW SNPs in 10-kb nonoverlapping windows (first plot, fig. S6).

We then calculated the difference between XY and ZW SNPs per 10-kb window and kept only outlier windows (second and third plots, fig. S6). We defined these outlier windows as windows with a difference of XY-ZW SNPs greater than the 75th percentile value +1.5 times the interquartile range. We then compared the distribution of XY and ZW SNPs in all outlier windows with a paired two-sided Mann-Whitney test (fourth plot, fig. S6). If the two distributions were significantly different from each other (*P* value < 0.05), then we defined the heterogametic system as the distribution (XY or ZW) with the higher total amount of SNPs. As a last step, we quantified XY or ZW SNPs of outlier windows (depending on the previously defined heterogametic system) per reference LG (corrected by chromosome length) and defined as potential sex chromosome the LG(s) with a number of SNPs higher than the 75th percentile value +3 times the interquartile range. To keep only the most extreme outliers and to further avoid false positives, we kept only the LG(s) with a number of SNPs higher than the standard deviation for this final call. In species for which a heterogametic system was identified, we further visualized all SNPs of the outlier windows of that system along the genome for illustrative purposes (fifth plot, fig. S6).

### Final sex chromosome system definition

We inferred sex-linked chromosomes, sex-differentiated regions and heterogametic state (XY/ZW) per species from sex-association in GWAS (approach 1), the sex-specific allele test (approach 2), and species-specific sex-differentiated site accumulations identified via the allele differences test on the basis of transcriptomes (approach 3). For approaches 1 and 2, which at first resulted in tribe-level identification of sex chromosomes (columns B and C, table S2), we eventually made sex chromosome calls on the species level as follows: We required the same sex-linked region to be present in at least two species of a tribe to base a sex chromosomal call on WGS data only. This might underestimate the presence of sex chromosomes in our dataset but further reduces the number of false positives. On the basis of approach 3, which includes more individuals per species and was run on the species level, we could confirm the larger sex-differentiated regions identified by approaches 1 and 2. However, we failed to detect some of the rather small sex-linked regions with approach 3, such as the narrow ~5-kb region in Lamprologini, which we think is due to a combination of the low number of genes present in these regions, limiting signal to nonexpressed regions, and probably low levels of expression of these genes in adults. In these cases, approach 3 either returned no sex chromosomal signal for the species in question or supported the heterogametic system identified with the WGS data but showed no clear overrepresentation of sex-linked SNPs on a particular LG. Only in Eretmodini, transcriptome, and WGS data were conflicting to some extent in the sense that RNA data indeed supported LG10, which was also identified in approach 2, and additionally identified LG15 as sex-linked regions (not identified at the tribe level, species-specific) but failed to detect LG07 (identified by approach 2). The latter was thus excluded from the stringent dataset. For species, in which approaches 1 and/or 2 identified a sex chromosome but approach 3 returned no signal, we relied on the genomic data only (as for species without RNA sequencing data, detailed in table S2). Approach 3 not only allowed us to largely confirm sex chromosomes shared across species identified by approaches 2 and 3 but also characterized species-specific sex chromosomes that we would not call/identify otherwise [note that similar sample sizes and transcriptome approaches have previously been used to identify sex chromosomes, e.g., (*63*)]. These latter species did not show genotypes in the WGS data supportive of their tribe’s predominant system (i.e., they were classified as “no signal” on the basis of the WGS data and thus approaches 1 and 2). The effectiveness of our method is evidenced by our ability to identify the same signatures of sex linkage of all three previously identified sex chromosomes of LT cichlids (table S2) (*33*).

Still, given the reduced sample sizes for the small tribes in approach 2, we further decided to generate two sex chromosome call sets, a permissive dataset retaining all sex chromosomes identified by either approaches 1, 2, and 3 or combinations thereof, and a stringent dataset excluding all sex chromosomes that were exclusively identified in approach 2. We performed all subsequent analyses with both sets and report the results.

### Reconstruction of sex chromosome turnovers in cichlids

To reconstruct sex chromosome evolution across the LT radiation, we coded the final sex chromosome set as a probability matrix that included 14 different LGs identified in at least one species as sex-linked, incorporating the published data for two laboratory strains and a laboratory cross derived from a natural population of *A. burtoni* (*34*, *35*) and a population of *P. philander* (*19*) (permissive dataset; stringent dataset 13 LGs). Note that *P. philander* was present in the current dataset with a single individual only and the *A. burtoni* WGS samples included here derive from two different populations that, as of yet, lack proof of sex chromosomes not allowing the confirmation of previously published data. All further species, for which we could not identify any sex-linked LG and none was published to the best of our knowledge, were still included in the analysis and attributed equal probability for all 14 LGs (permissive dataset; 13 LGs in the stringent dataset). For these species, the presence or absence of genetic SD would need to be determined in the future. We placed the sex chromosome identities onto a time-calibrated phylogeny of LT cichlids (*17*), which we pruned with phytools to include only the species studied here. We followed the approach described by Jeffries *et al.* (*15*) and inferred ancestral sex chromosome states using a stochastic mapping approach implemented in phytools. We compared the likelihood scores (based on the Akaike information criterion) for three different transition rate models—equal rates, symmetrical rates, and all rates different (ARD)—which identified ARD as the best model for transition rates between states. We simulated 1000 stochastic character maps along the phylogeny. In addition, we separately ran stochastic mapping for each chromosome, coding the use of the chromosome as a sex chromosome in a given species as a binary (yes or no) trait to account for the fact that some tips of the phylogeny are in two or more states (i.e., two or more reference LGs showed sex linkage likely because of chromosomal rearrangements/fusions) rather than having the equal probability of being in one of two states. Note that for *A. burtoni*, four different LGs have been proposed as sex chromosomes in different strains (*34*, *35*). We then combined the 14 separate reconstructions (permissive dataset; 13 in the stringent dataset) into one phylogenetic representation. The results obtained with the two approaches were very similar, and we hence continued calculations with the binary reconstructions.

We determined the time points of sex chromosome turnover events as points on branches where the inferred probability of using a given chromosome as a sex chromosome dropped below 0.5 for the first time starting from the tips of the phylogeny with the function densityMap of phytools. On the basis of the study by Jeffries *et al.* (*15*), we did not consider species that had no detectable sex chromosome as having losses but only considered transition events that led to the emergence of a new sex chromosome, i.e., we only retained gains.

Likewise, we ran a second independent analysis with 1000 stochastic mappings to reconstruct ancestral states for the type of heterogamety (XY/ZW). In addition to the reconstructed turnover points, we here added a turnover on the terminal branch leading to *A. burtoni*, because for this species, both XY and ZW sex chromosomes have been described (*35*).

To test whether gene content or chromosome size drives the observed pattern of sex chromosome recruitment in LT cichlids, we randomly picked 30 times (the number of sex chromosome recruitments derived from ancestral state reconstruction) a window of 10 kb of the reference genome and attributed the LG containing this window as sex chromosome to a species. We simulated this operation 10,000 times and counted how many times each LG was recruited in each simulation. We then counted in how many simulations nine or more LGs that were not recruited, as this was the observed pattern. We then tested for an association of the number of sex chromosome turnovers leading to the tips of each tribe with the number of species investigated in each tribe with a pGLS using the R package ape (v.5.2) (*64*).

### Reconstruction of sex chromosome turnovers in other teleosts

We then ran the same two analyses for ricefishes (Adrianichthyidae), which, to the best of our knowledge, are the only fish family with detailed data on sex chromosomes with synteny inference based on a comparison to a common reference genome (*Oryzias latipes*). We derived information on sex chromosomes from Hilgers and Schwarzer (*27*) and placed it on a time-calibrated phylogeny of the family Adrianichthyidae (19 species, table S4), extracted from a recent comprehensive ray-finned fish phylogeny (*36*). We could not include sex chromosome data of three species (*Oryzias sakaizumii*, *Oryzias wolasi*, and *Oryzias woworae*), as these were not included in the phylogeny and no other comprehensive time-calibrated tree comprising these species was available to us.

To compare our data on a macroevolutionary scale, we calculated transition rates for ray-finned fishes of the Tree of Sex database (http://treeofsex.org/). We used the data for all Tree of Sex species that were also included in the recent comprehensive ray-finned fish phylogeny (*36*) (table S5). As several species names were not initially included in the phylogeny (*36*), we inspected species names of Tree of Sex for typos, older versions of species names, and synonyms in FishBase (www.fishbase.org) and Eschmeyer’s Catalog of Fishes Online Database (https://calacademy.org/scientists/projects/eschmeyers-catalog-of-fishes) and corrected the names accordingly. This allowed us to map SD data for 472 species from the Tree of Sex database onto the phylogeny. We further added published data for cichlids [(*19*, *33*–*35*) and this study], resulting in an additional 72 species. We simplified SD data from the Tree of Sex database and coded the data as a probability matrix with three states, namely, XY (including species classified by Tree of Sex as “XY heteromorphic,” “XY homomorphic,” “XO,” or “XY polygenic”), ZW (including species classified by Tree of Sex as “ZW heteromorphic,” “ZW homomorphic,” “ZO,” or “ZW polygenic”), and “NonGSD” (including species classified by Tree of Sex as “apomictic,” “hermaphrodite,” “ESD_other,” “pH,” “size,” “density,” “TSD,” or “other”). The final matrix is provided in table S5. Similar to our strategy described above, we included all other species with no information on SD with an equal probability for all three states.

### Convergent evolution of sex chromosomes

We detected the same region on LG19 as sex-linked in species belonging to the tribes Haplochromini and Perissodini. Within Haplochromini, this sex-linked region was present in six species of the genus *Tropheus* (tribe Tropheini, the endemic LT Haplochromini) and in *O. indermauri* (a distantly related riverine haplochromine from the Lufubu River, which drains into LT). Our ancestral state reconstruction suggested an independent origin of the LG19 SD system in Perissodini, *Tropheus*, and *O. indermauri.* To further investigate the hypothesis of convergence, we extracted all SNPs from LG19 that were XY in at least one of the species with a positionally overlapping XY system from a variant call file containing all species investigated in the present study (*17*) with VCFtools. In addition to a male and a female of these eight species, we included representatives without this XY-LG19 system from the other tribes (a male and a female of each of *Xenotilapia flavipinnis*, *Plecodus elaviae*, *Petrochromis trewavasae*, *Eretmodus cyanostictus*, *Benthochromis tricoti*, *C. leptosoma*, *Limnochromis auritus*, and *Cyphotilapia frontosa*) and other haplochromine species (a male *P. philander*, a male and a female *A. burtoni*, and a male *Ctenochromis polli*). We only kept variants with less than 10% missing data. We next extracted the two haplotype sequences of each individual for all variants in FASTA format. Assuming that the variant phasing with Beagle was not error free across whole chromosomes, we inspected the haplotypes and corrected the phasing for the eight LG19-XY species. We did this so that for sites where an XY male was heterozygous while the corresponding XX female was homozygous, the allele of the male shared with the female was designated as haplotype 1 (the presumed X allele) and the other allele as haplotype 2 (the presumed Y allele). We then inferred a phylogenetic tree by maximum likelihood with IQ-TREE (v.1.7-beta12) (*65*) under the GTR+F+ASC substitution model to account for ascertainment bias and assessing branch support with 1000 ultrafast bootstrap approximations. We rooted the obtained phylogenetic tree in accordance with the species tree (Fig. 1).

### Defining the degree of sex chromosome differentiation, potential sex-determining regions, and candidate genes

On the above-defined sex chromosomes, we characterized species-specific sex-differentiated regions by counting the numbers of XY and ZW SNPs in windows of 10 kb. The density of XY or ZW windows is shown in fig. S10. We defined the size of the sex-differentiated region as the proportion of the LG covered by windows that have a density of sex-specific SNPs that is more than twice as high as the genome-wide mean over all windows such that the sum of all sex-differentiated windows defines the cumulative length of the sex-differentiated regions and the minimum and maximum window coordinates define the range of the sex-differentiated region on the LG. We tested for an association between sex chromosome differentiation and the estimated age of origin of the sex chromosome derived from the turnover point with a pGLS using the R package ape. From the results of approaches 1 to 3, we identified sex-differentiated regions shared between several species and overlaid these with candidate genes involved in SD and pigmentation. We defined pigmentation genes in the reference genome over gene ontology annotations including the term “pigmentation” and its child terms. We also retrieved orthologous sequences of the Nile tilapia to the medaka pigmentation genes defined by Braasch *et al.* (*66*) over Biomart, Ensembl release 96 (www.ensembl.org). Because this Nile tilapia genome is a different genome release from the reference genome used by us, we searched the NCBI database for the obtained Ensembl gene IDs and translated them to the assembly version that we used with the NCBI Genome Remapping Service. Candidate genes for SD included genes previously identified through a literature search (*67*, *68*) and a gene ontology analysis based on a gene ontology annotation matching the word “sex” (list of gene IDs of candidate genes for SD and pigmentation in table S6). We further investigated all annotated genes that were partially or fully included in the window(s) with the maximum number of sex-specific SNPs on the sex chromosome (table S2).

### Statistical analyses

We report statistical parameters and applied tests in the main text, corresponding Materials and Methods sections, and figure legends where appropriate. We performed all statistical analyses in R (v.3.5.0 and v.3.5.2, detailed above, including used R packages).

## Notes

View/request a protocol for this paper from *Bio-protocol*.
