# Supplementary material for: Dynamics of sex chromosome evolution in a rapid radiation of cichlid fishes
Source: Sci Adv. 2021 Sep 1;7(36):eabe8215. doi: 10.1126/sciadv.abe8215 (PMC8442896; doi:10.1126/sciadv.abe8215)
Supplement: Supplementary file 1 — Figs. S1 to S11 [file sciadv.abe8215_sm.pdf]

Science Advances
AAAS

Supplementary Materials for

Dynamics of sex chromosome evolution in a rapid radiation of cichlid fishes

Athimed El Taher, Fabrizia Ronco, Michael Matschiner, Walter Salzburger, Astrid Böhne*

*Corresponding author. Email: a.boehne@leibniz-zfmk.de

Published 1 September 2021, *Sci. Adv.* **7**, eabe8215 (2021)
DOI: 10.1126/sciadv.abe8215

The PDF file includes:

Figs. S1 to S11

Other Supplementary Material for this manuscript includes the following:

Tables S1 to S6

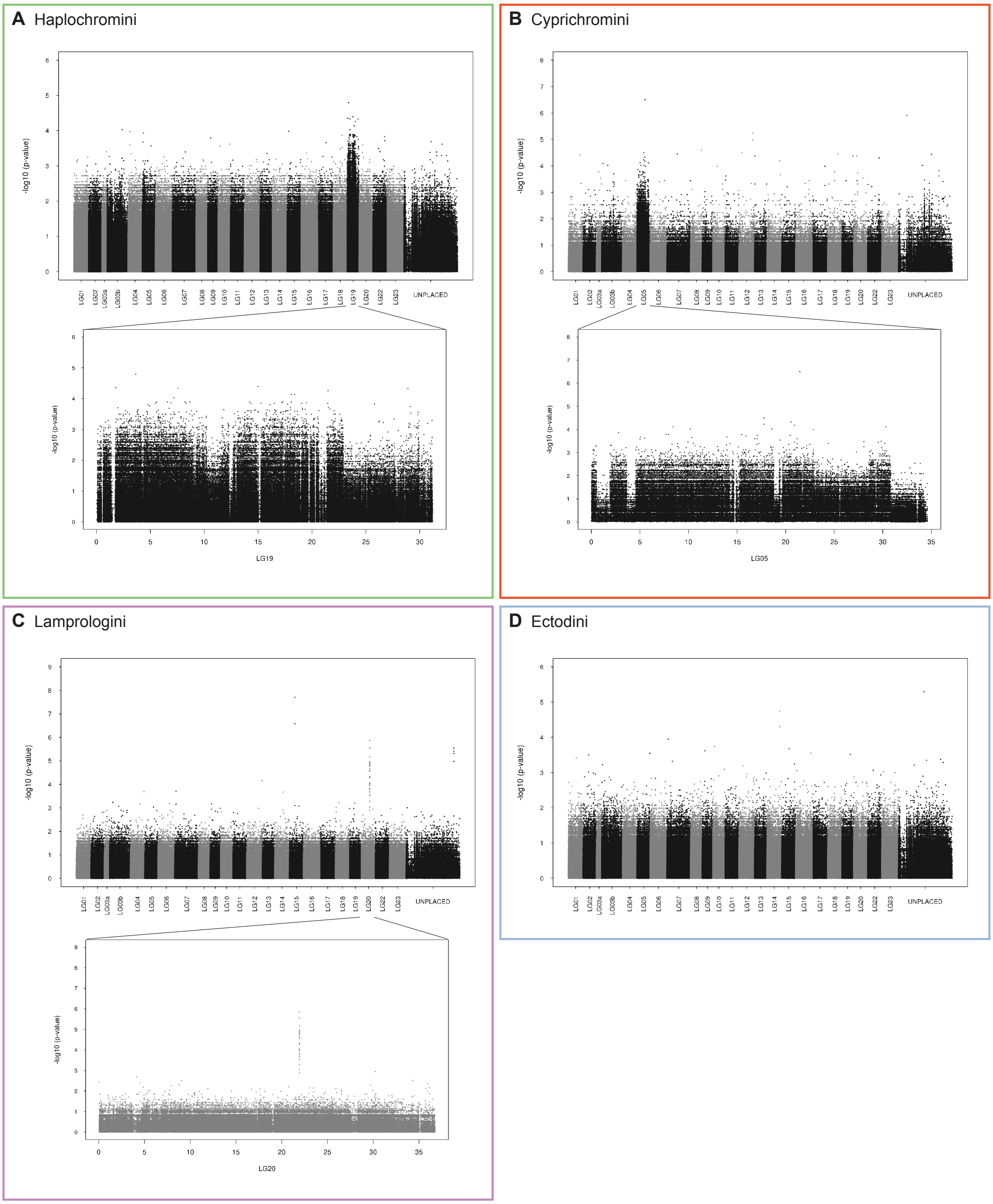

Fig. S1. Results of tribe-wise GWAS for an association with sex (approach 1). Manhattan plots of a GWAS analysis for an association with sex for the tribes Haplochromini (**A**), Cyprichromini (**B**), Lamprologini (**C**), and Ectodini (**D**) using the reference genome of the Nile tilapia (*Oreochromis niloticus*). Interchanging black and grey colors delimit chromosomes. Unplaced scaffolds were concatenated into an "UNPLACED" chromosome for visualisation. Insets show zooms of regions with an accumulation of SNPs that show an association with sex, i.e. sex chromosomes.

Fig. S2

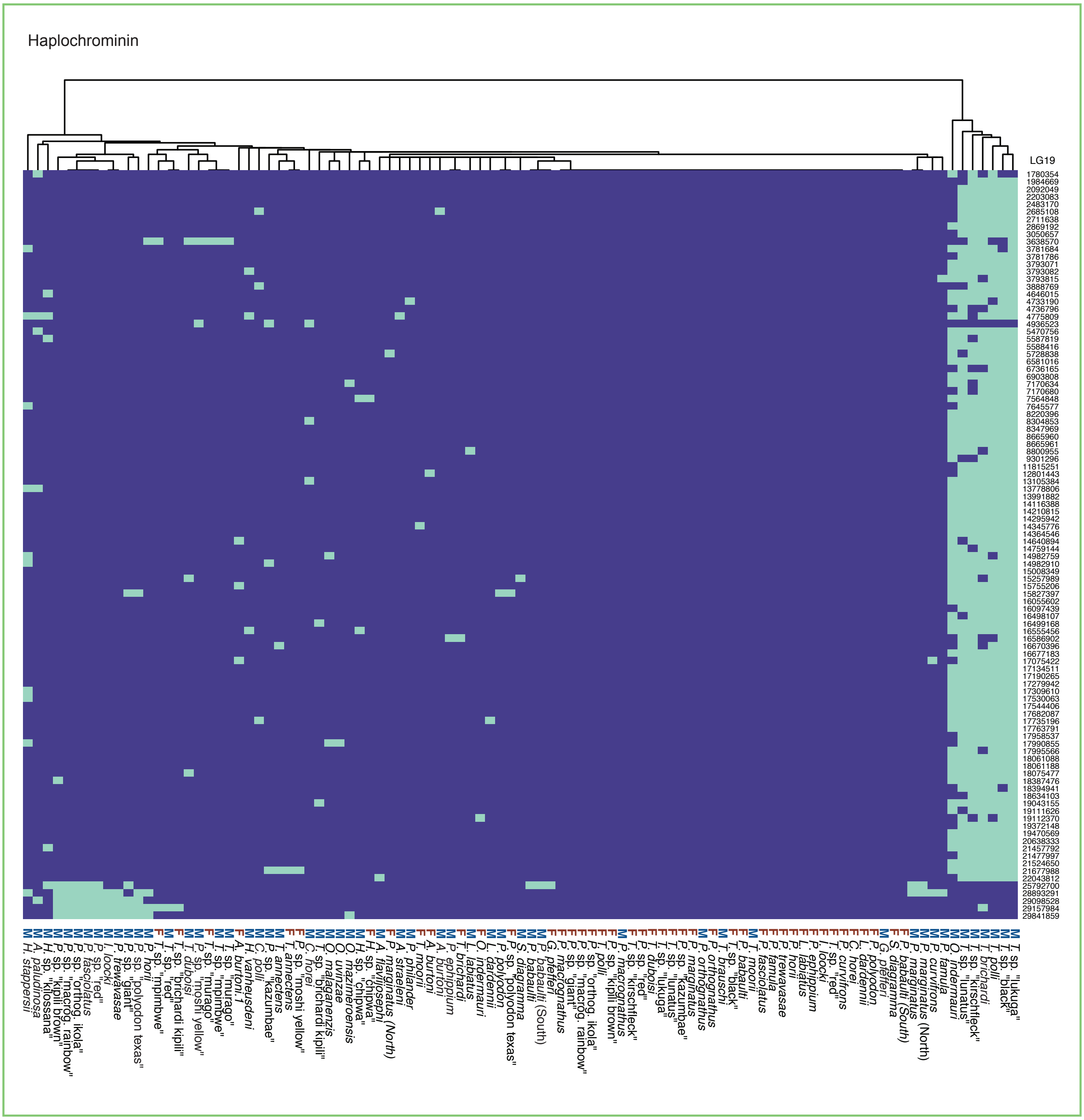

Fig. S2 (continued)

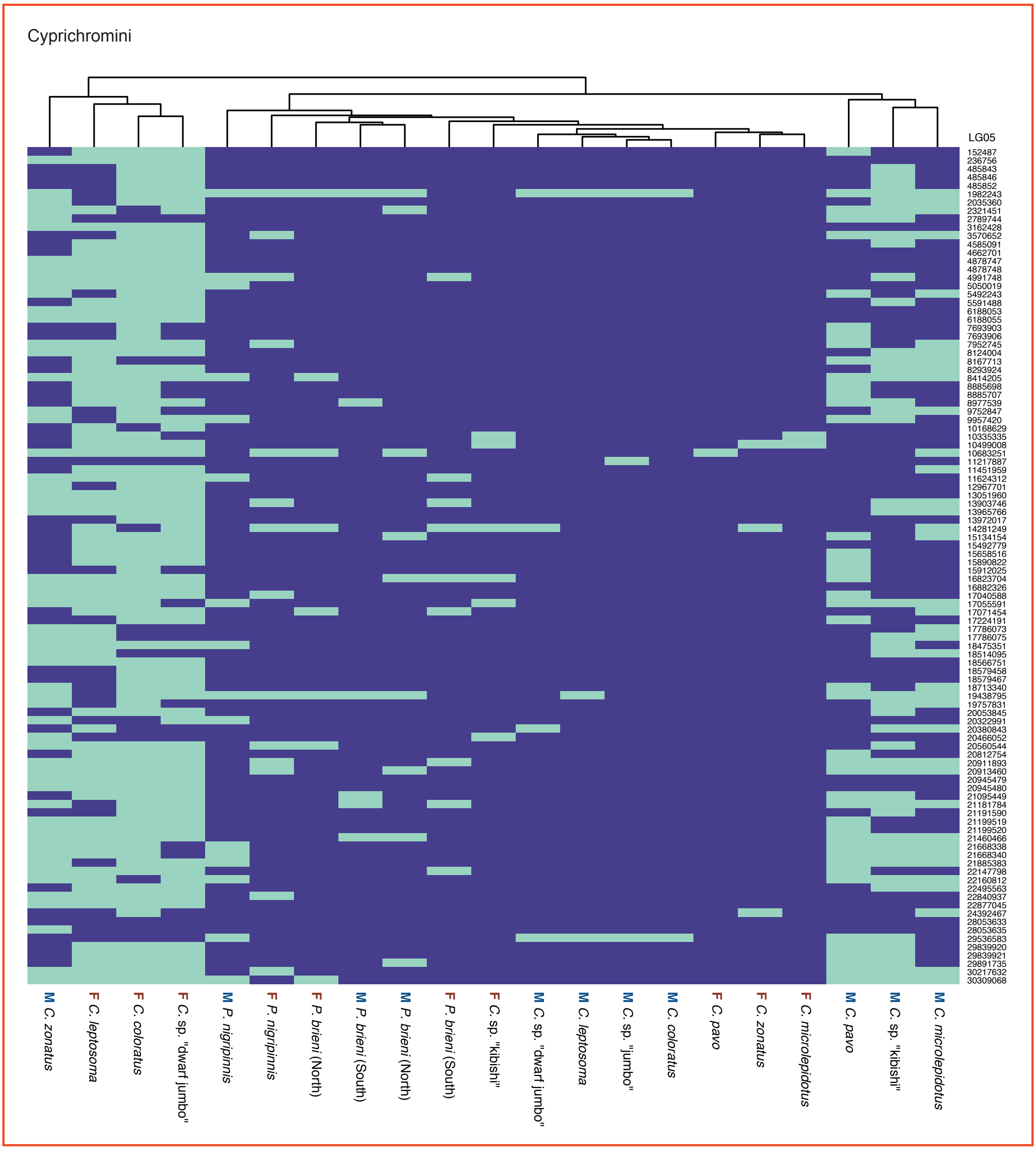

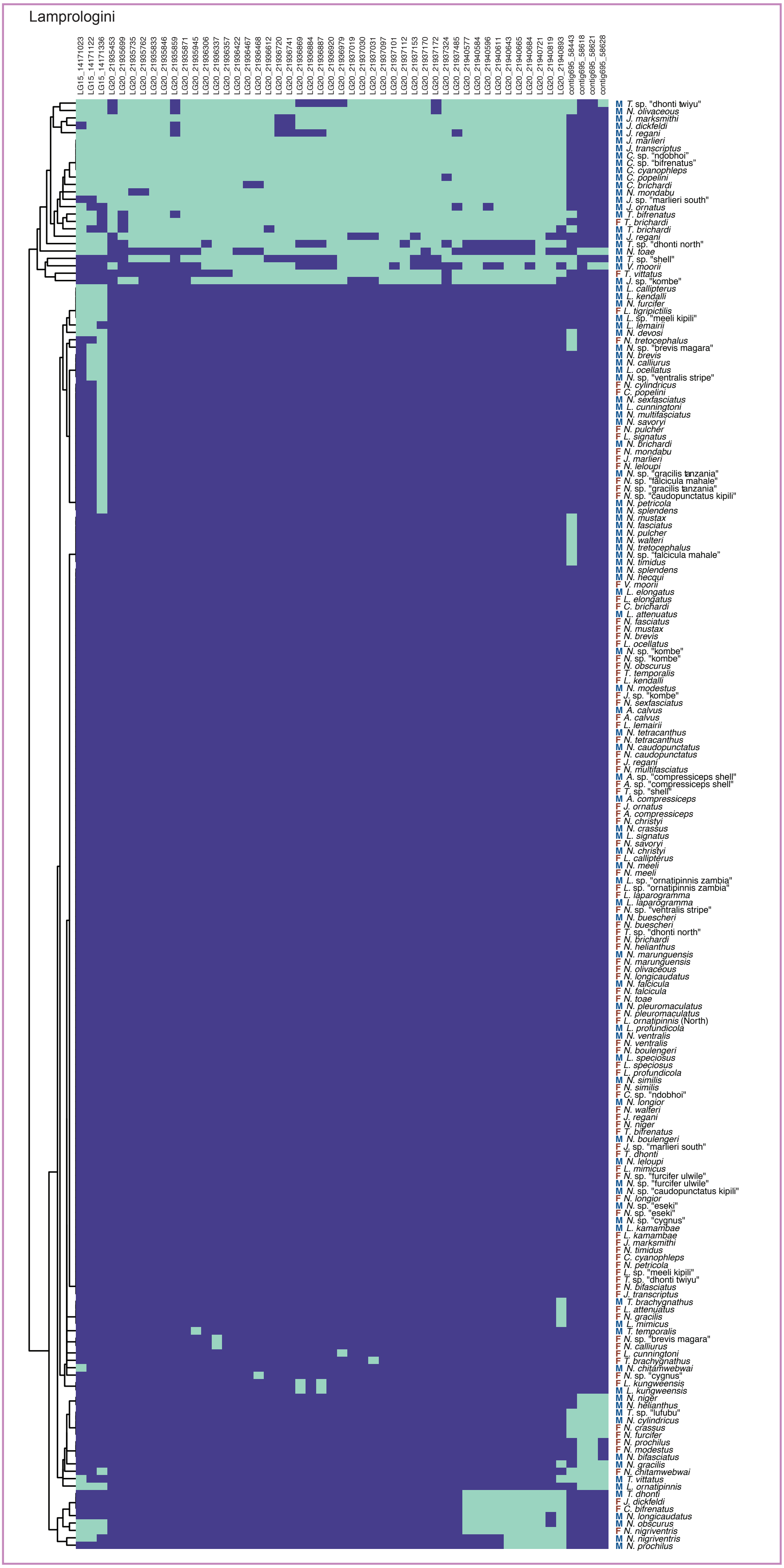

Fig. S2. Genotypes of GWAS outlier regions (approach 1). Heatmaps show individual genotypes for outlier SNPs (F: female individuals, M: male individuals; Haplochromini and Cyprichromini 100 most significant SNPs, Lamprologini three outlier regions on LG15, LG20 and an unplaced contig) of sex-linked regions detected by GWAS shown in fig. S1 for all individuals analysed by GWAS; purple: homozygous; green: heterozygous.

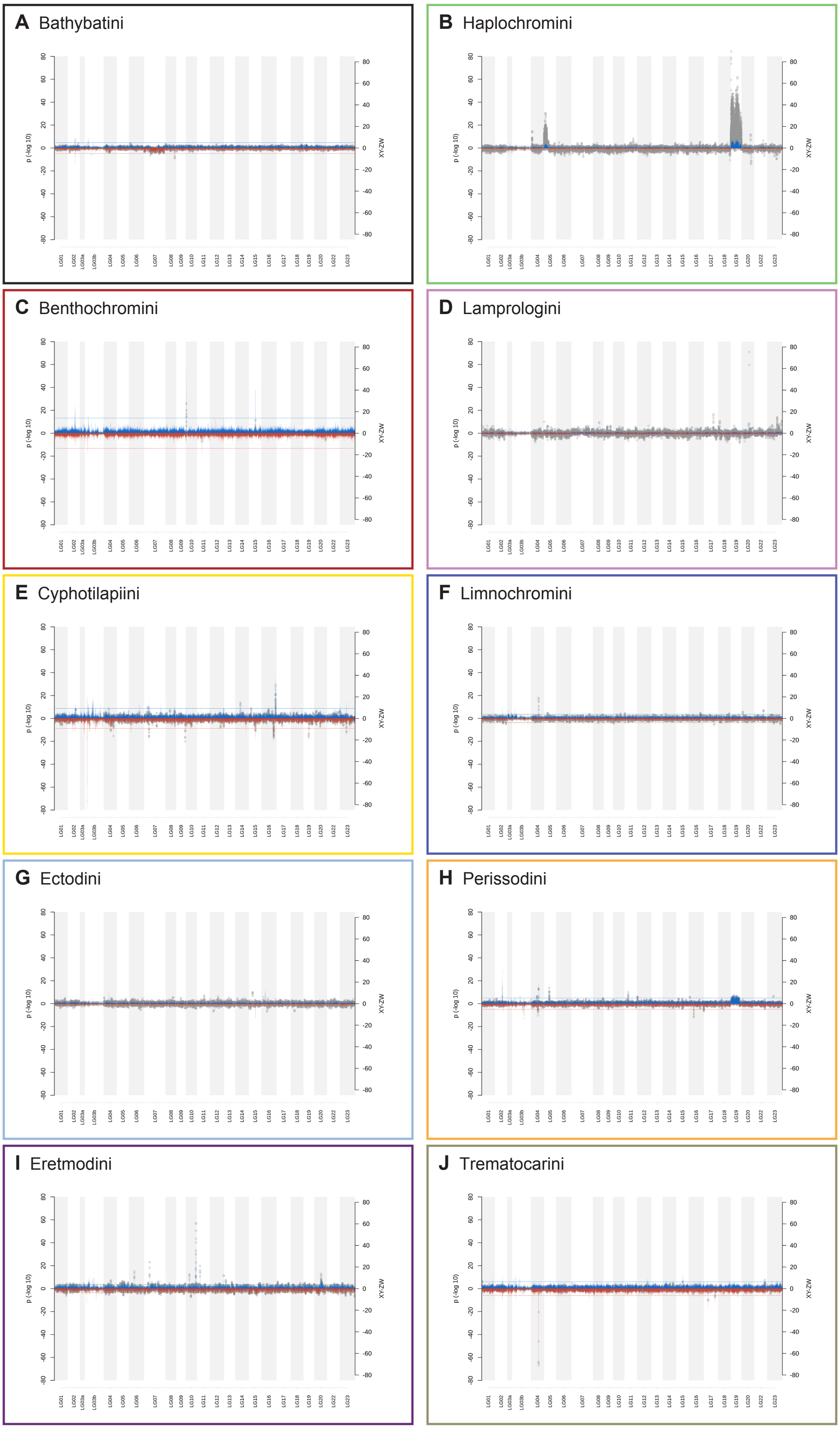

Fig. S3. Sex-specific SNP windows (approach 2) for the tribes Bathybatini (A), Haplochromini (B), Benthochromini (C), Lamprologini (D), Cyphotilapiini (E), Limnochromini (F), Ectodini (G), Perissodini (H), Eretmodini (I), and Trematocarini (J). Gray dots show results for tests of overrepresentation of XY- (left Y-axis, positive scale) and ZW- (left Y-axis, negative scale) SNPs in windows of 10 kb with a slide of 2 kb. Repetitive sequence regions on LGs 02, 03a and 03b were excluded in the final call set of sex chromosomes and are plotted in lighter gray. Background shadings refer to LGs of the reference genome. Vertical lines indicate XY-ZW SNP difference per window normalized by the number of species (right Y-axis), windows with more XY-SNPs are plotted with blue lines, those with more ZW-SNPs with red lines. Dotted horizontal lines indicate the largest absolute XY-ZW difference normalized by species number obtained in each tribe over 100 permutations (right Y-axis).

Fig. S4

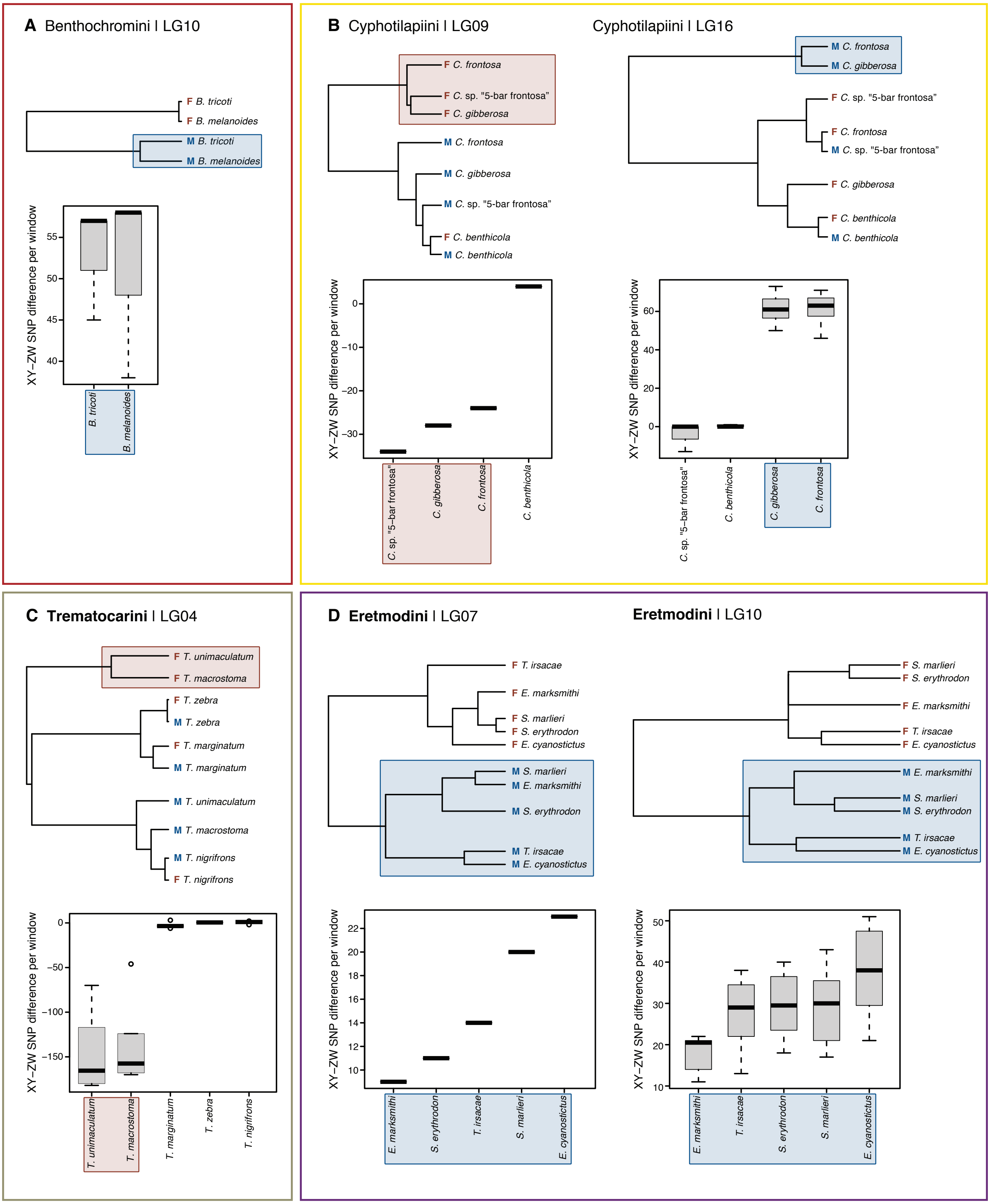

A Benthochromini I LG10
F B. tricoti
F B. melanoides
M B. tricoti
M B. melanoides
XY–ZW SNP difference per window
B. tricoti
B. melanoides
B Cyphotilapiini I LG09
F C. frontosa
F C. sp. "5-bar frontosa"
F C. gibberosa
M C. frontosa
M C. gibberosa
M C. sp. "5-bar frontosa"
F C. benthicola
M C. benthicola
Cyphotilapiini I LG16
C Trematocarini I LG04
F T. unimaculatum
F T. macrostoma
F T. zebra
M T. zebra
F T. marginatum
M T. marginatum
M T. unimaculatum
M T. macrostoma
M T. nigrifrons
F T. nigrifrons
D Eretmodini I LG07
Eretmodini I LG10
F T. irsacae
F E. marksmithi
F S. marlieri
F S. erythrodon
F E. cyanostictus
M S. marlieri
M E. marksmithi
M S. erythrodon
M T. irsacae
M E. cyanostictus

Fig. S4 (continued)

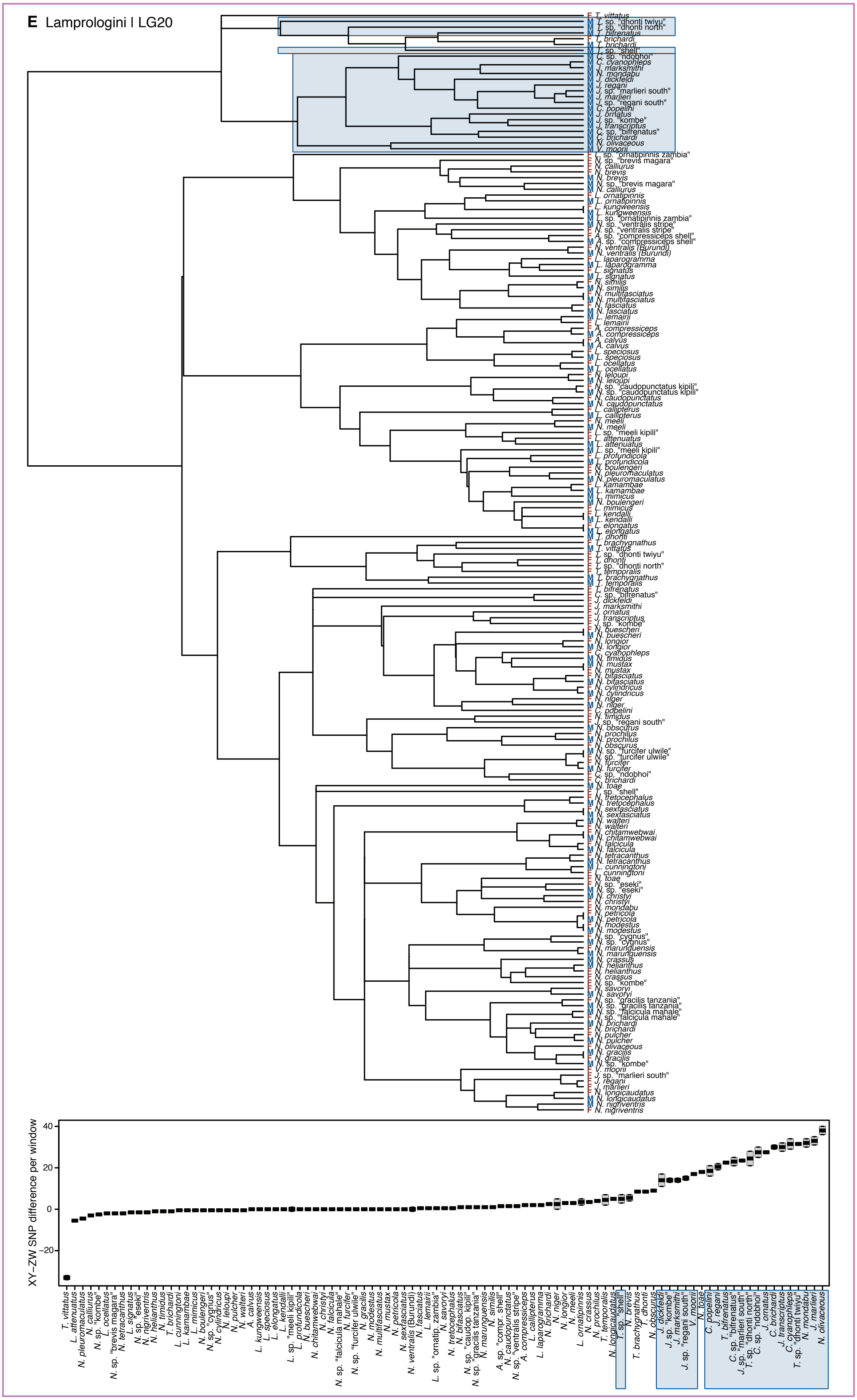

Fig. S4 (continued)

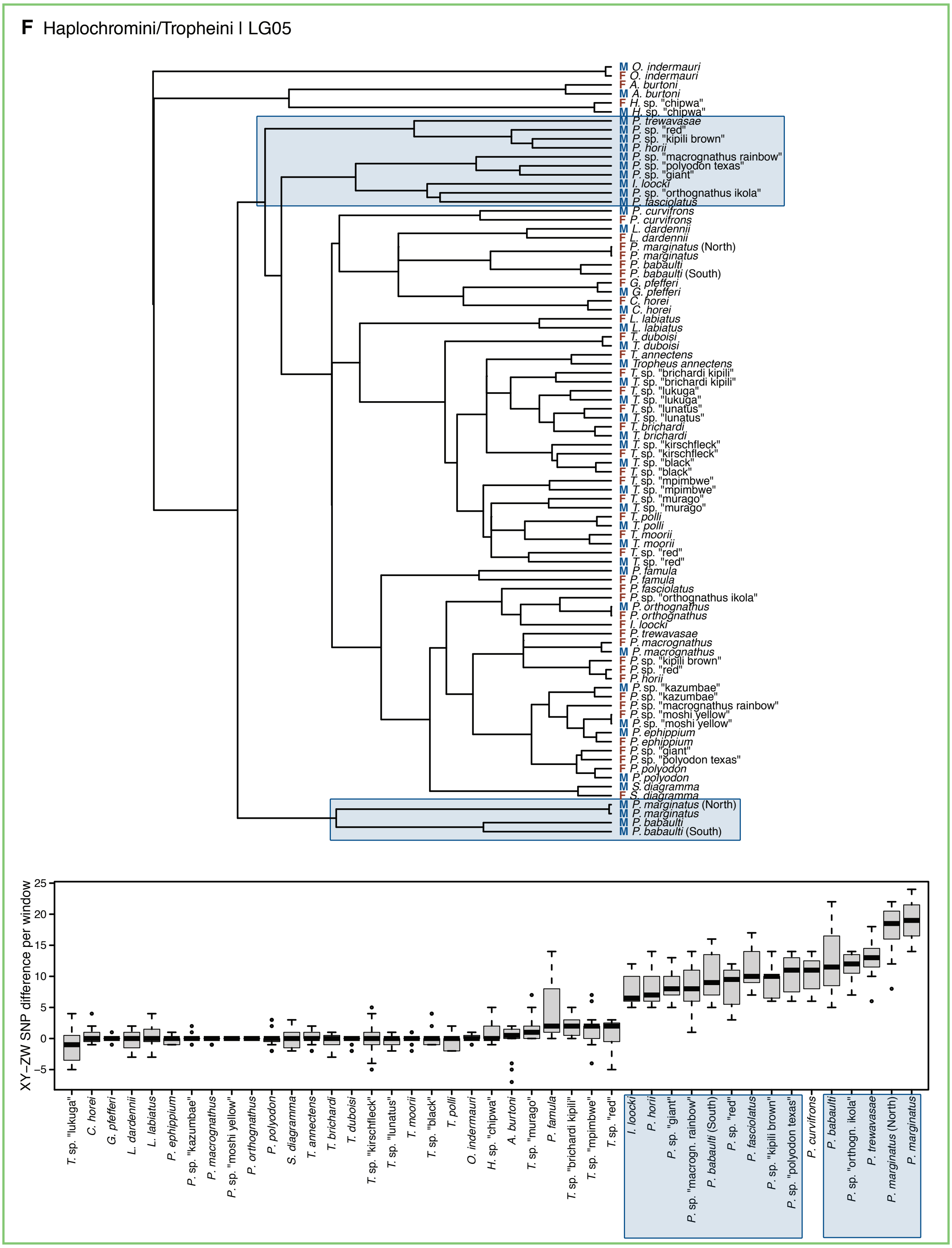

Fig. S4 (continued)

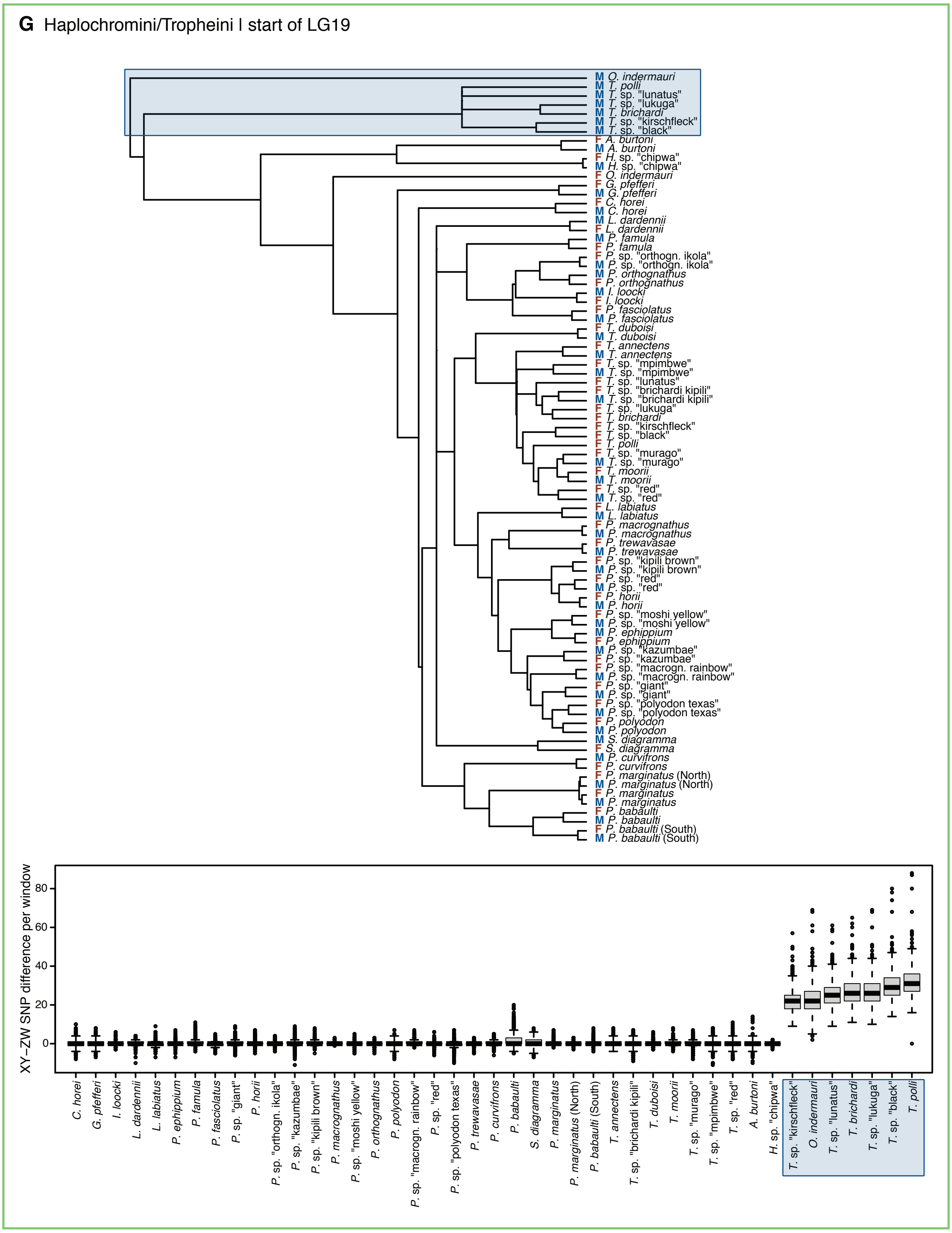

Fig. S4 (continued)

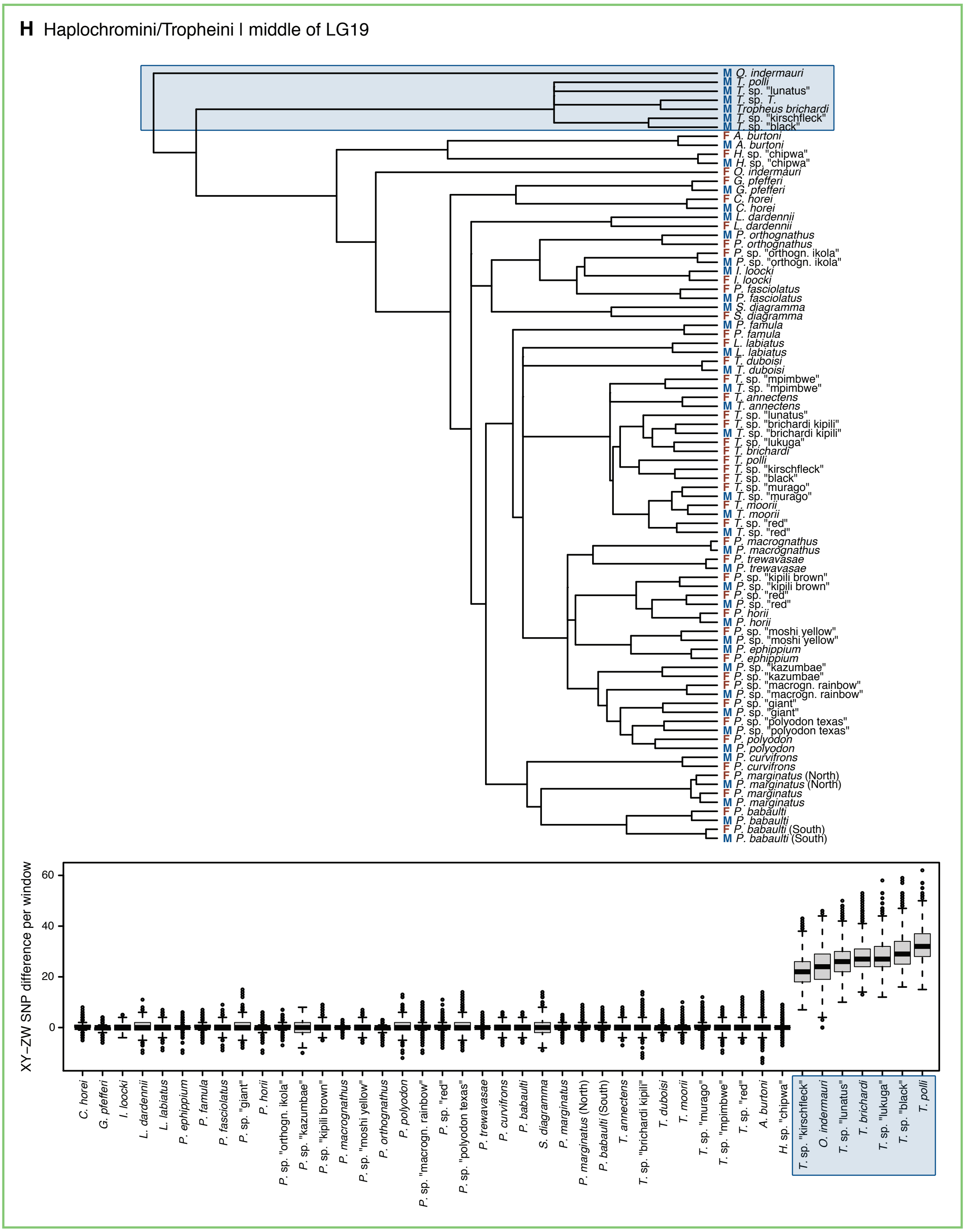

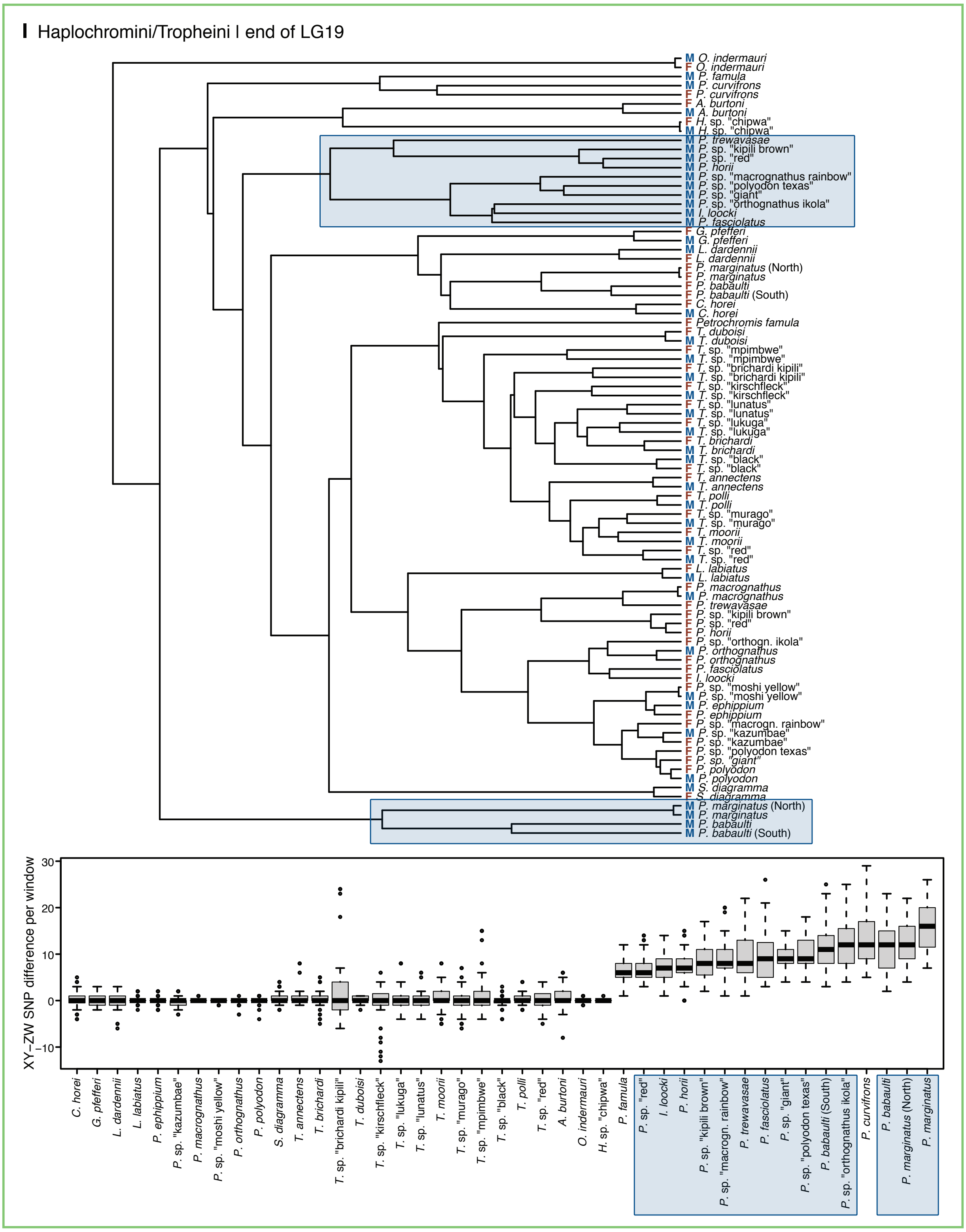

Fig. S4. Species values of sex-specific outlier regions (approach 2) for the tribes Benthochromini (A), Cyphotilapiini (B), Trematocarini (C), Eretmodini (D), Lamprologini (E), and Haplochromini (F-I). Dendograms of samples based on divisive hierarchical clustering of genotype values in outlier windows of approach 2 with individuals clustering by sex colored in blue for XY-systems and in red for ZW-systems and boxplots of per species XY-ZW difference in XY/ZW outlier windows (fig. S3) with color-coding based on the dendograms. Analysis include all individuals analysed by approach 2 (fig. S3). Boxplot center lines represent the median, box limits the upper and lower quartiles, and whiskers the 1.5x interquartile range; points represent outliers. F: female individuals, M: male individuals.

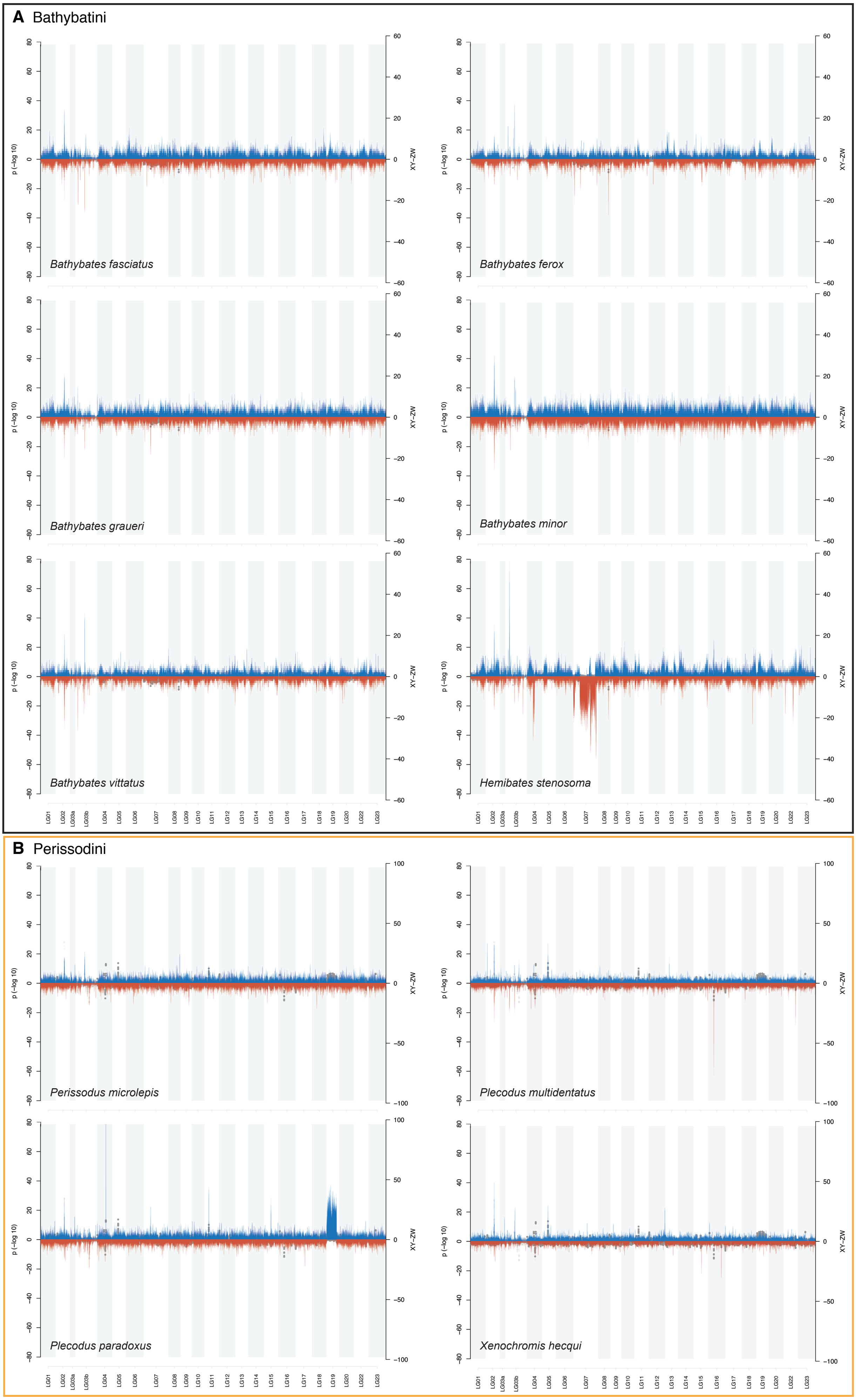

Fig. S5. Chromosome-wide sex-specific alleles in *Hemibates stenosoma* and *Plecodus paradoxus*. Visualization of per species XY-ZW differences in windows of 10 kb with a slide of 2 kb overlaid on the tribewise test results for sex-specific SNPs for Bathybatini (**A**) and Perissodini (**B**) (fig. S3).

Fig. S6

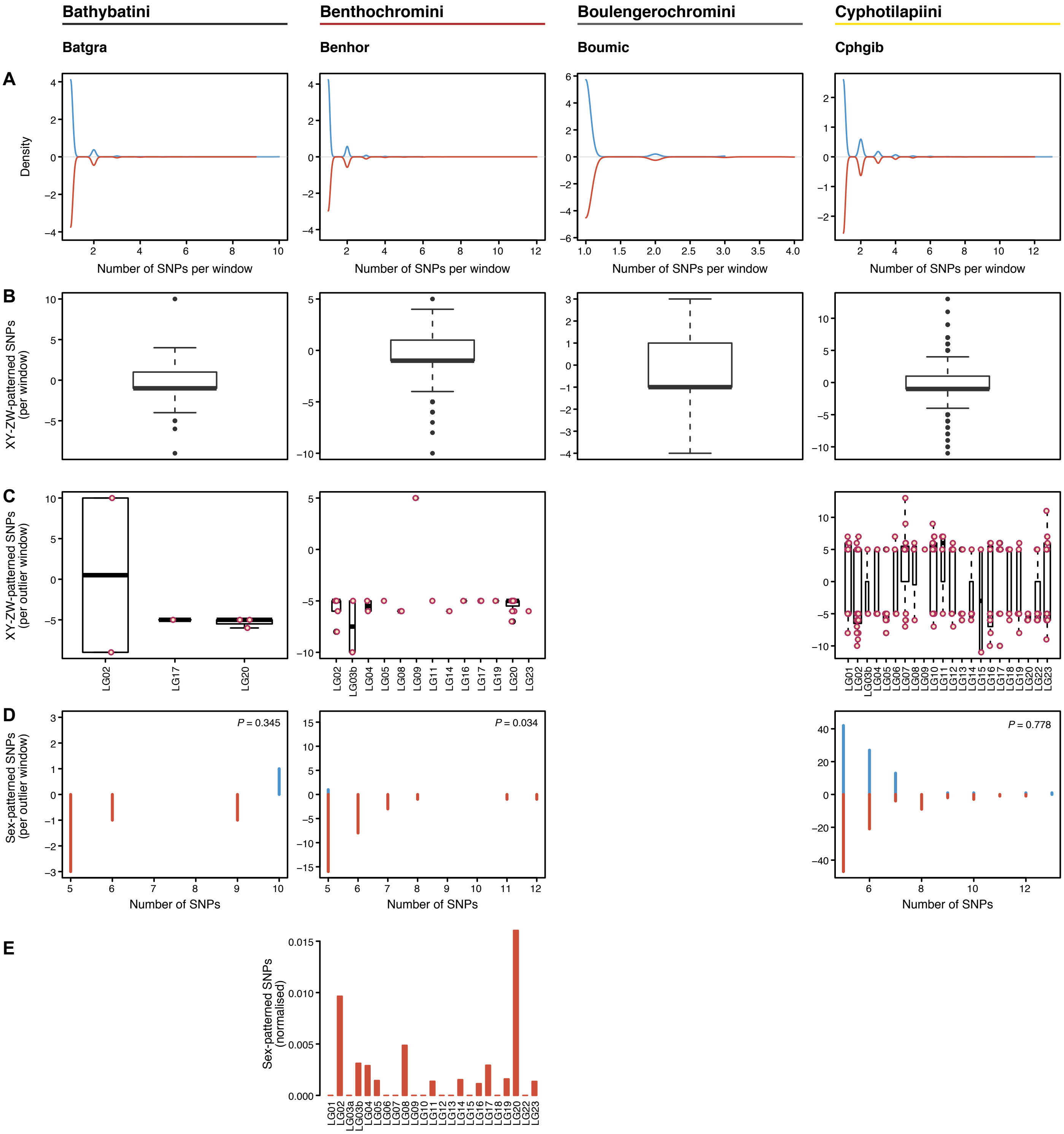

Bathybatini
Batgra
Benthochromini
Benhor
Boulengerochromini
Boumic
Cyphotilapiini
Cphgib
A
B
C
D
E
Density
Number of SNPs per window
XY-ZW-patterned SNPs (per window)
XY-ZW-patterned SNPs (per outlier window)
Sex-patterned SNPs (per outlier window)
Number of SNPs
P = 0.345
P = 0.034
P = 0.778
Sex-patterned SNPs (normalised)

Fig. S6 (continued)

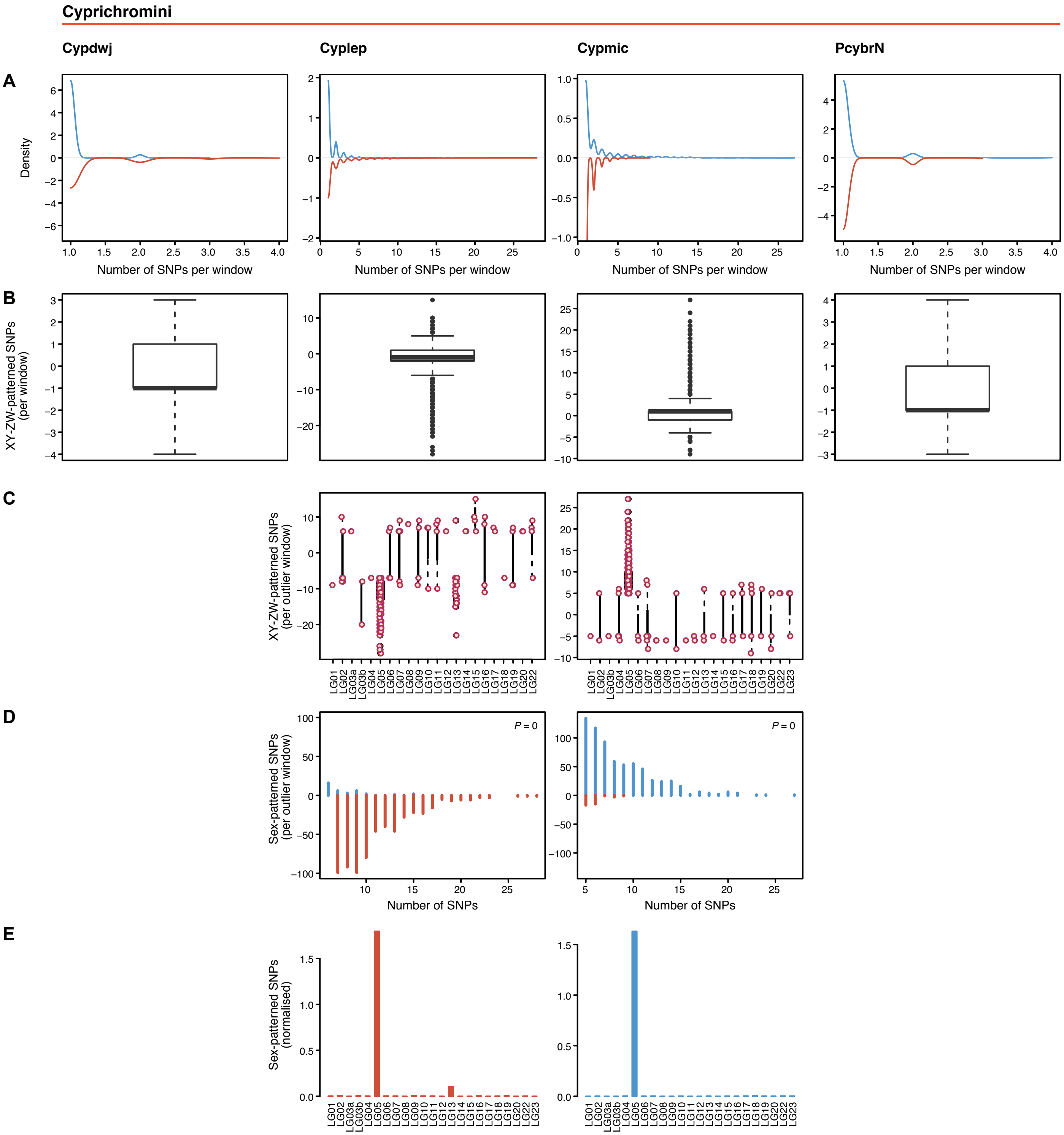

Fig. S6 (continued)

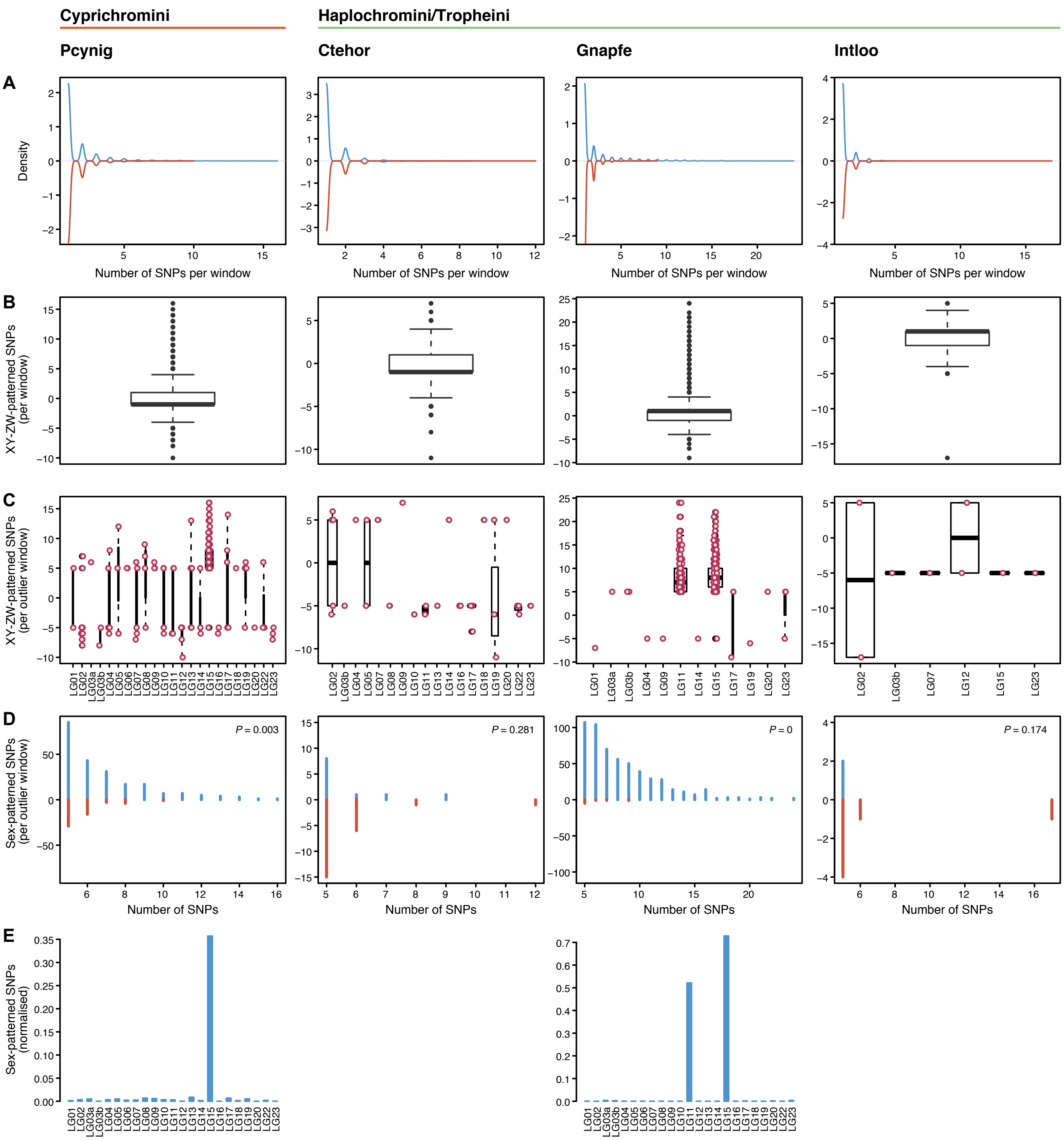

Fig. S6 (continued)

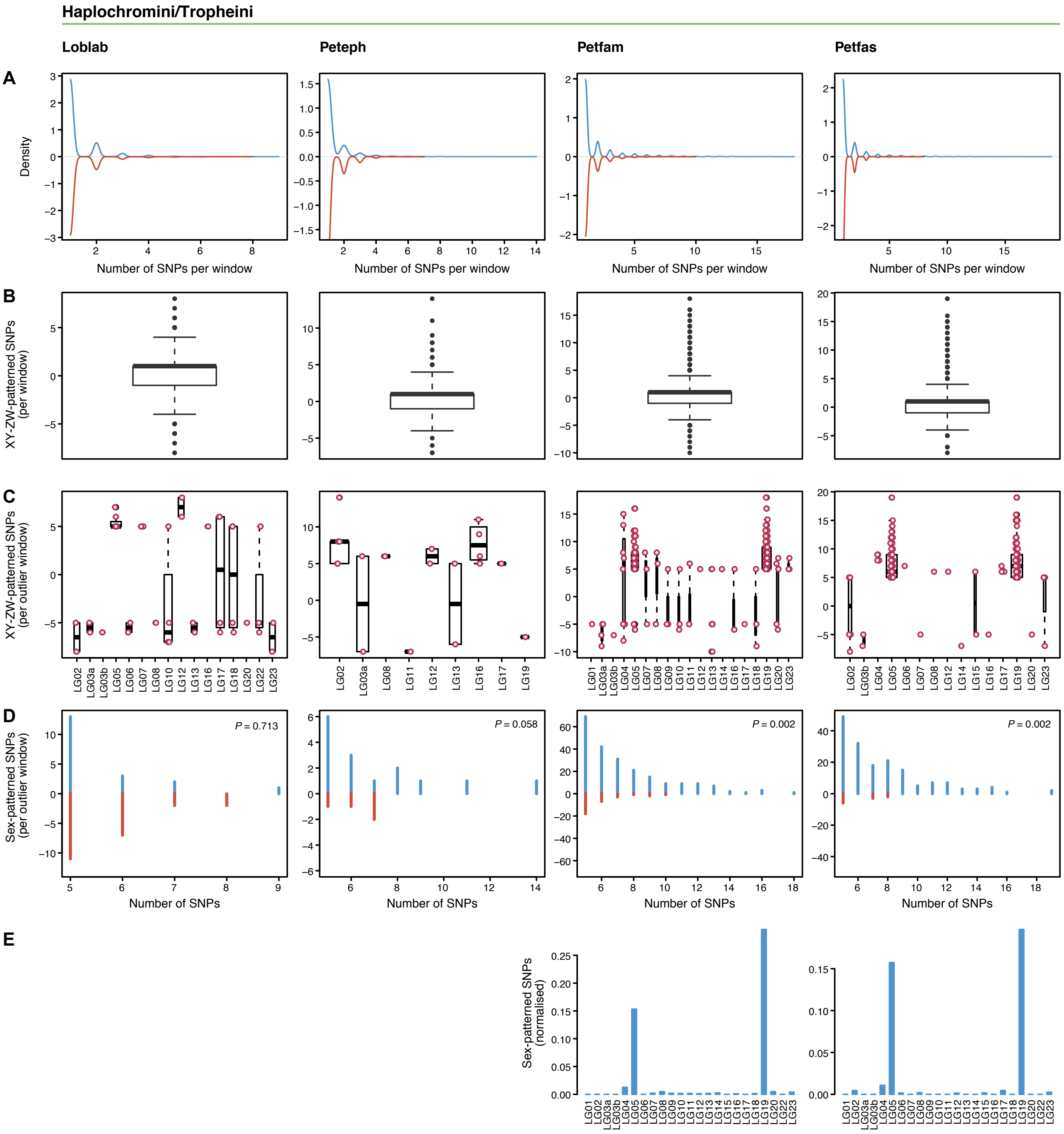

Fig. S6 (continued)

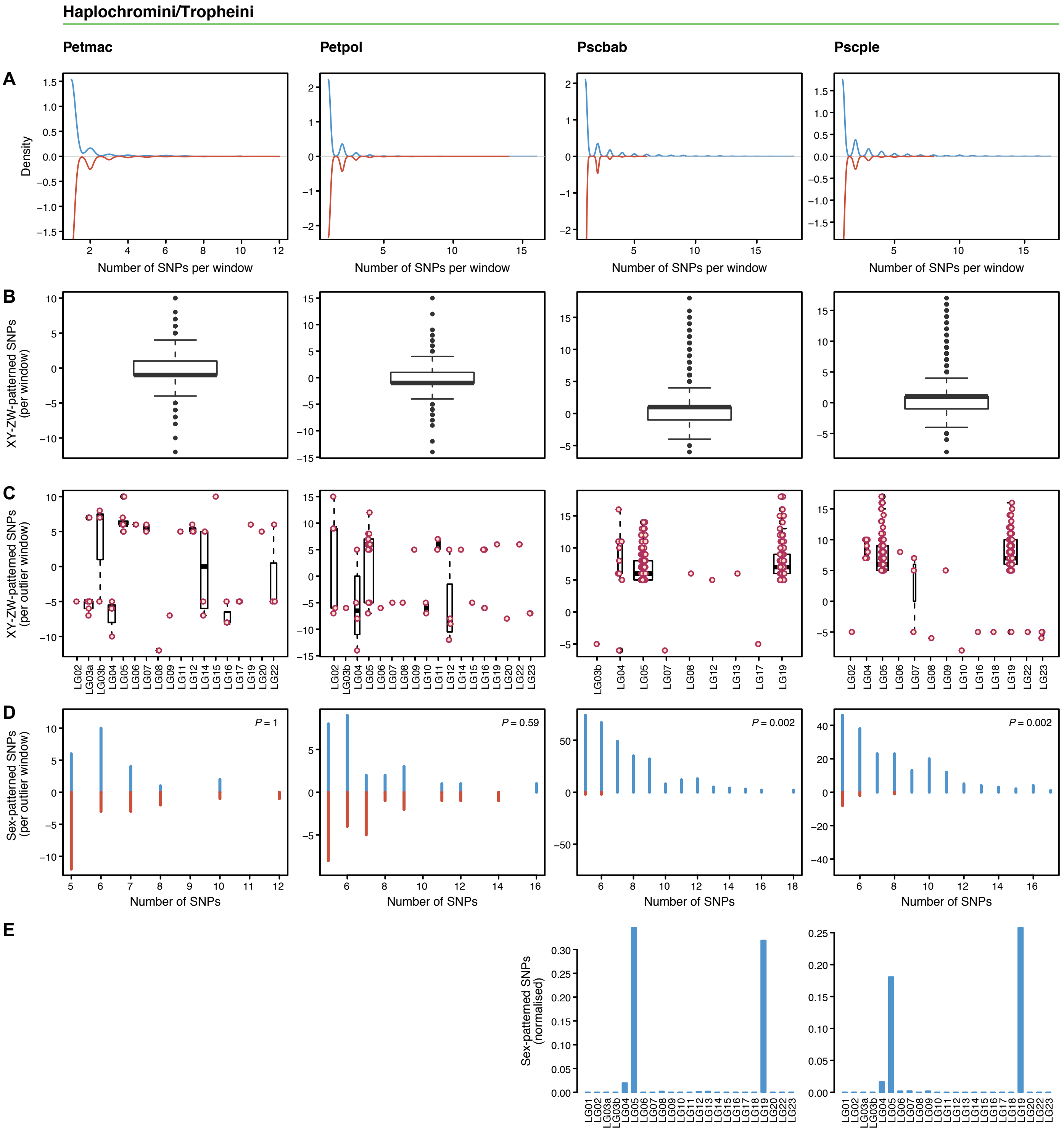

Fig. S6 (continued)

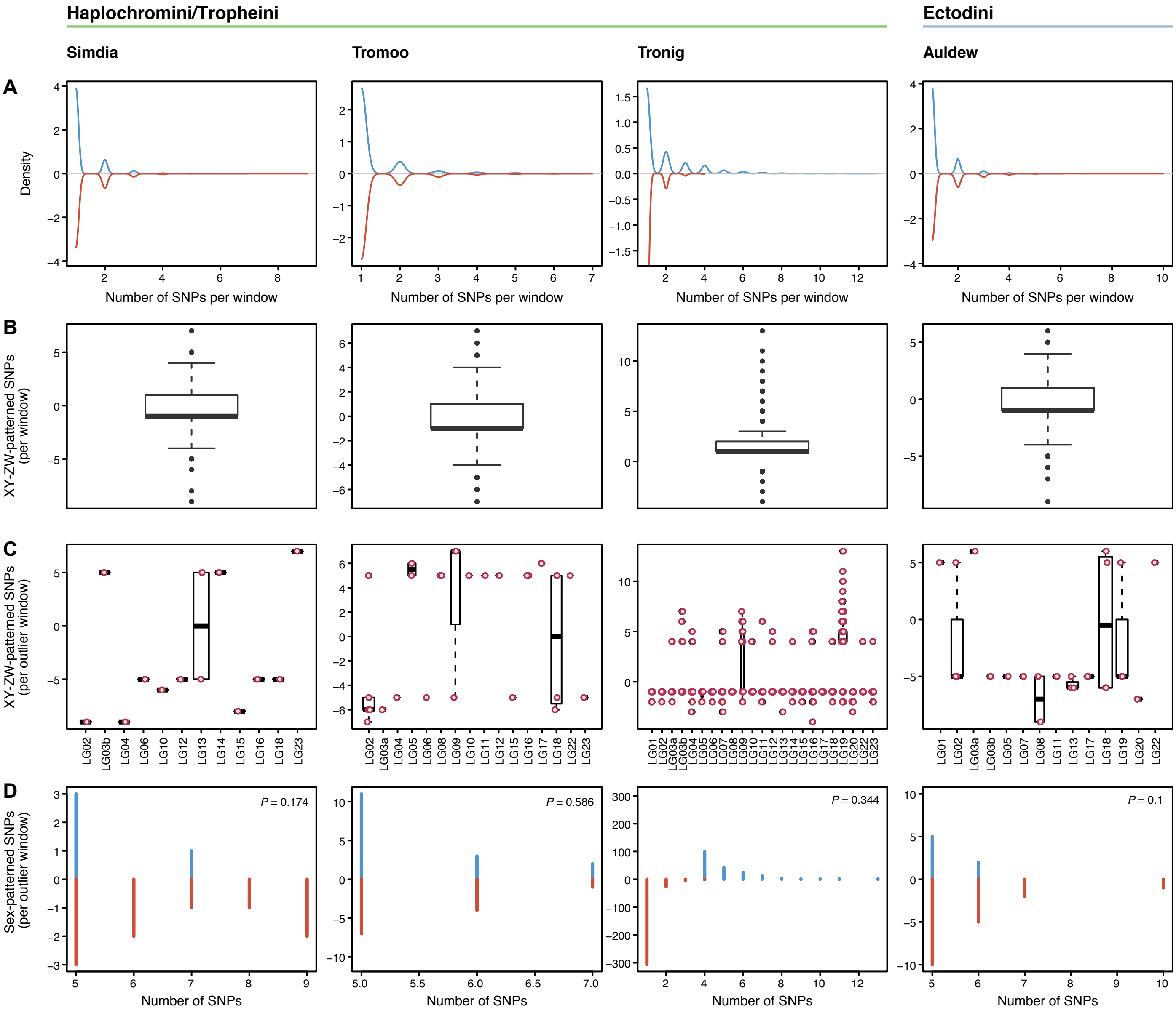

Fig. S6 (continued)

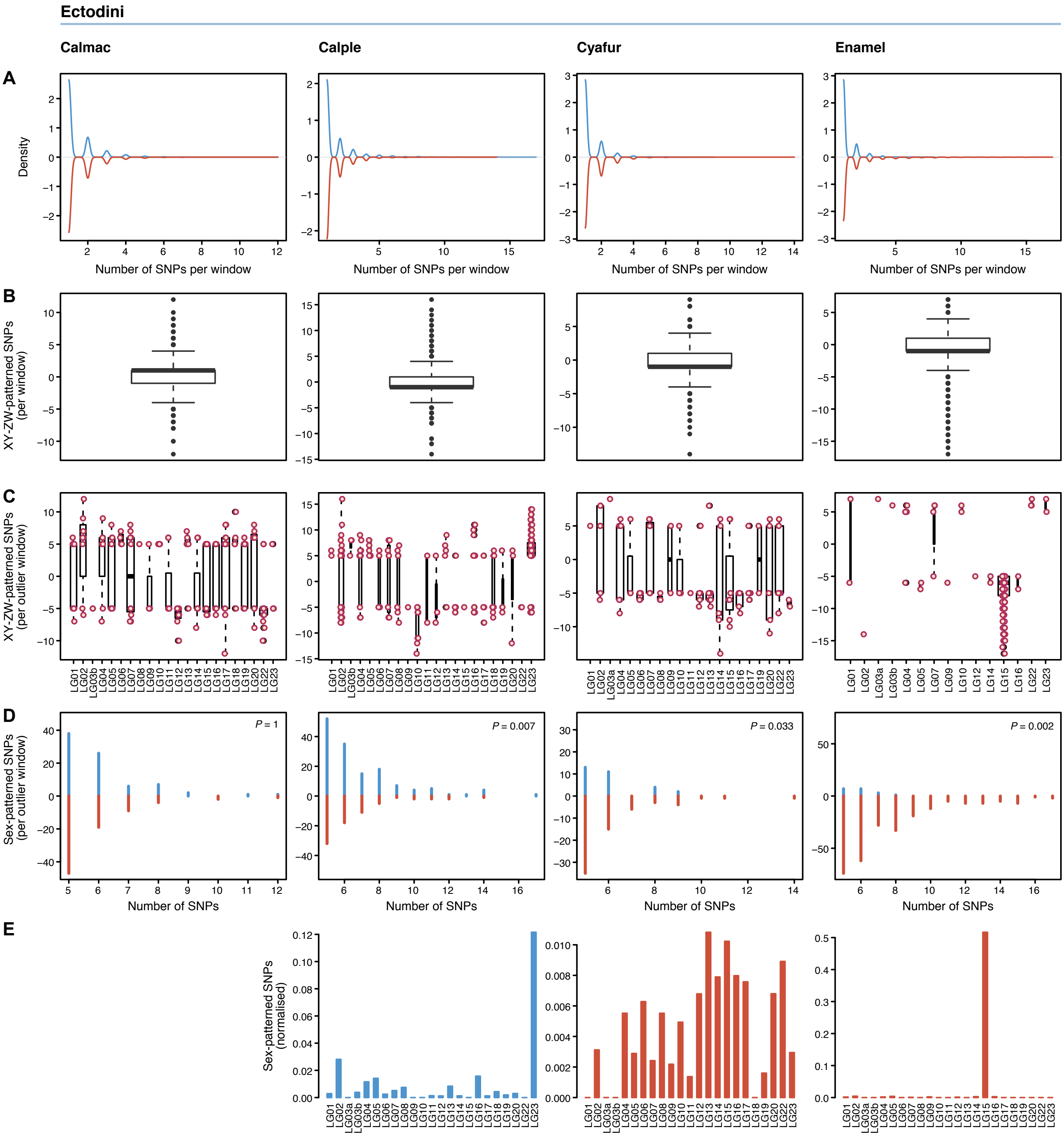

Fig. S6 (continued)

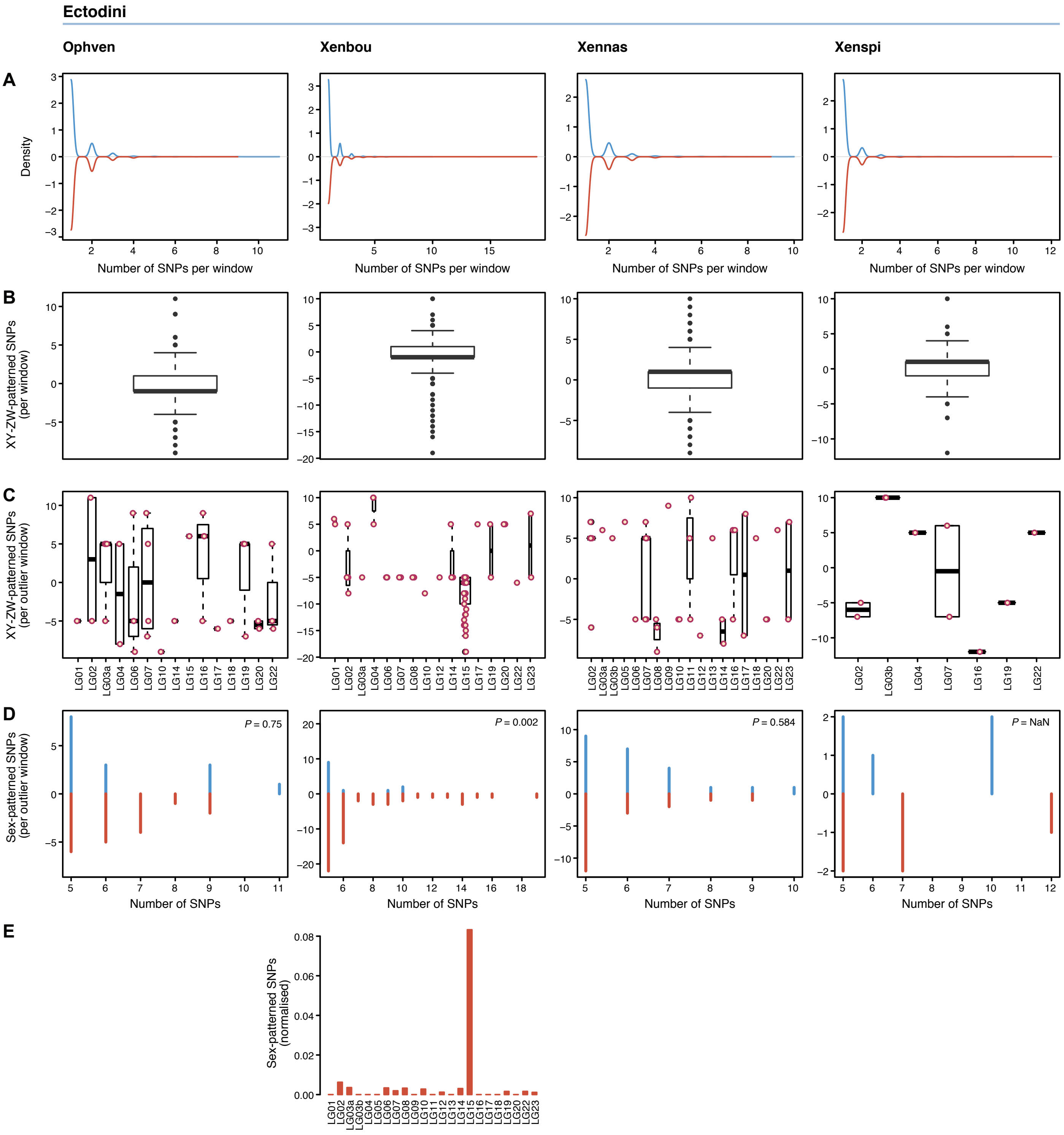

Fig. S6 (continued)

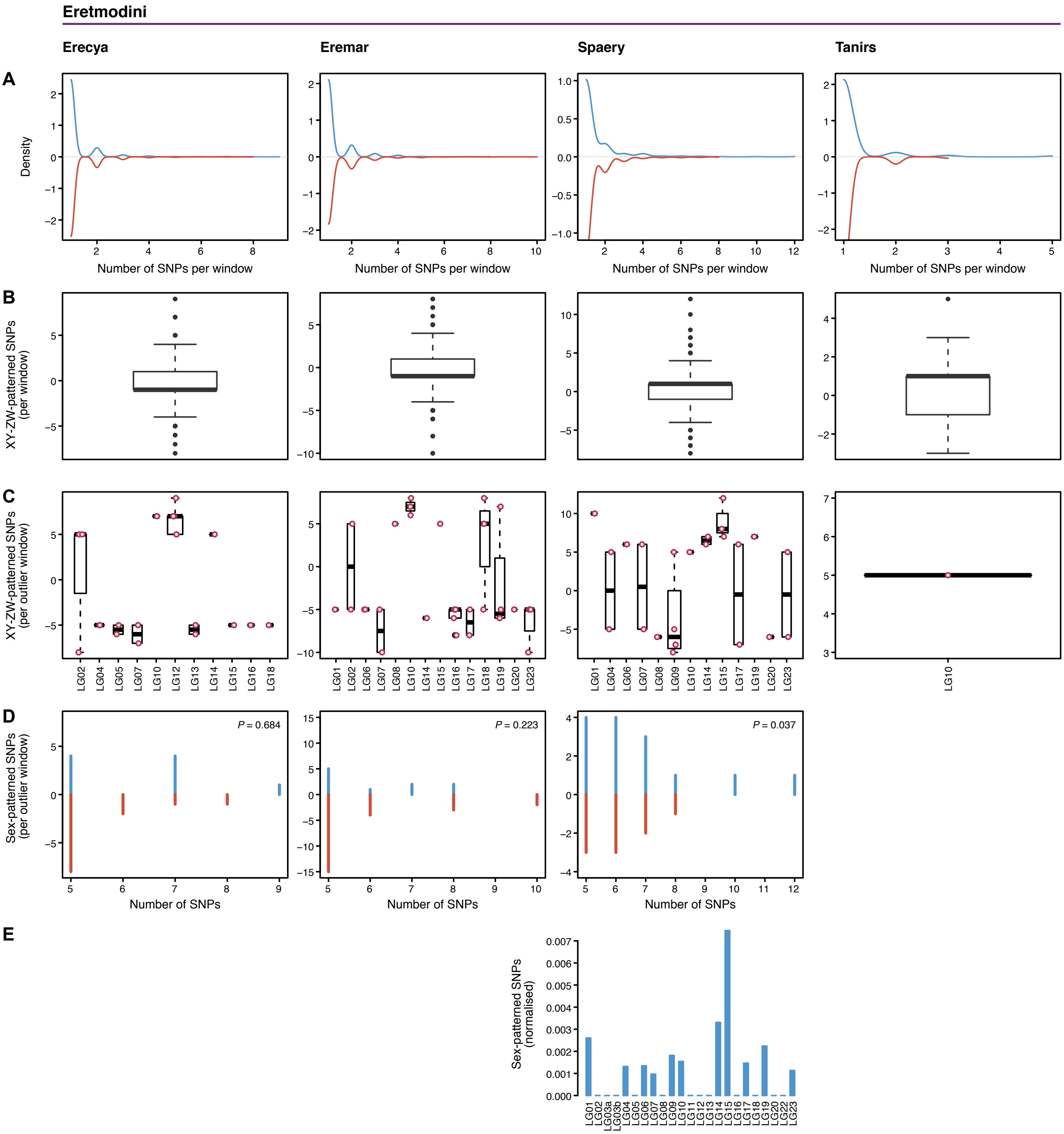

Fig. S6 (continued)

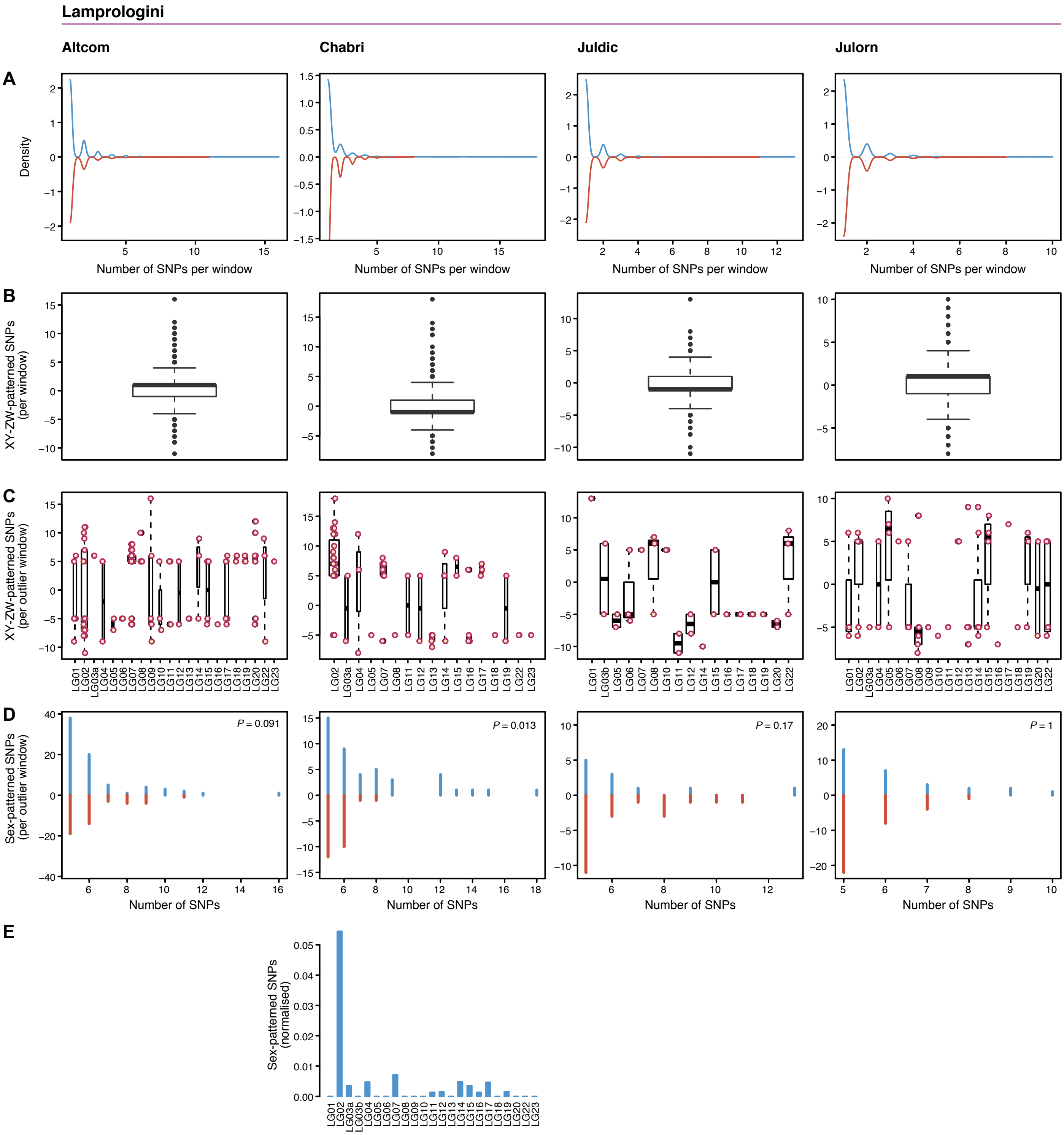

Fig. S6 (continued)

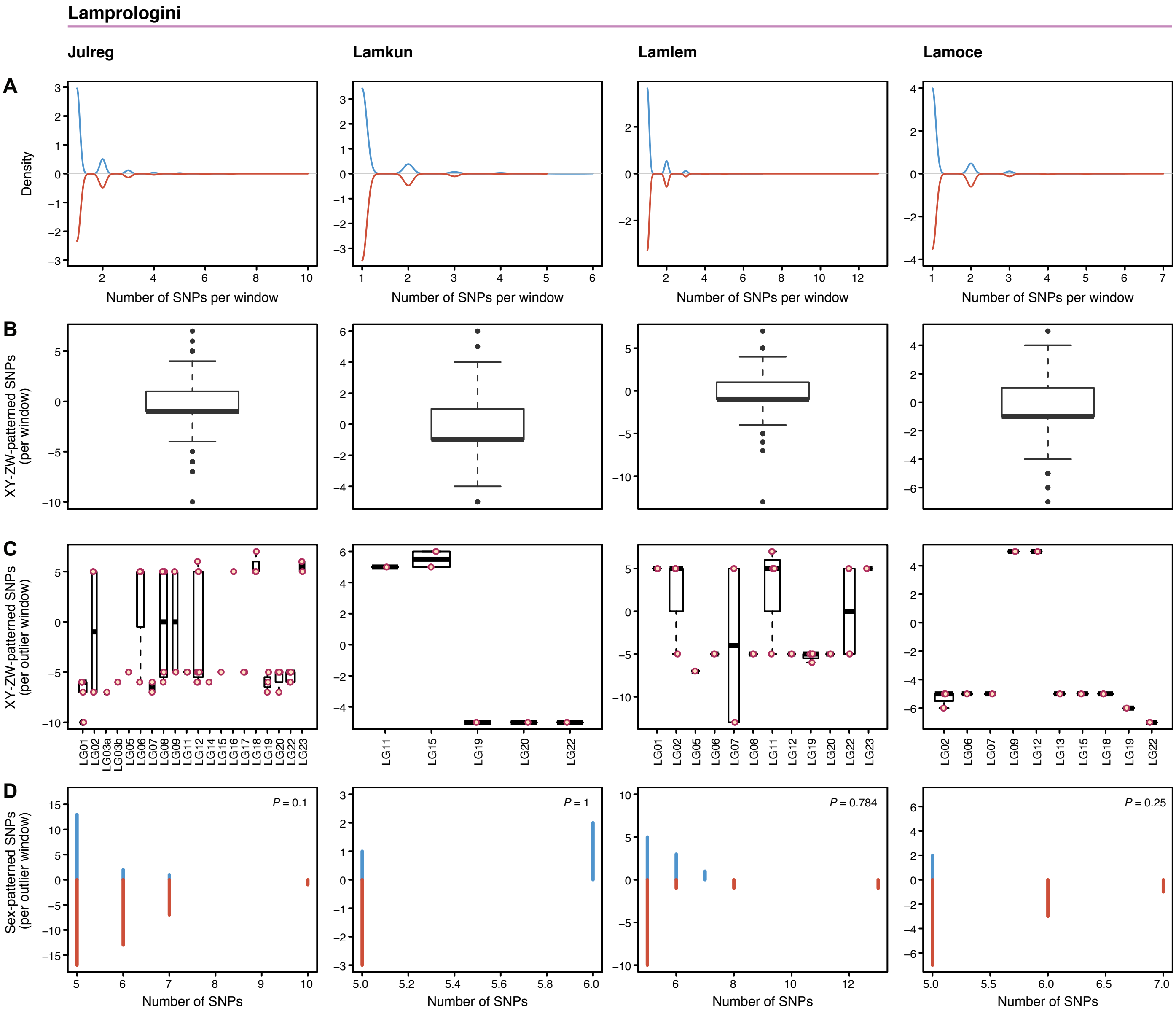

Fig. S6 (continued)

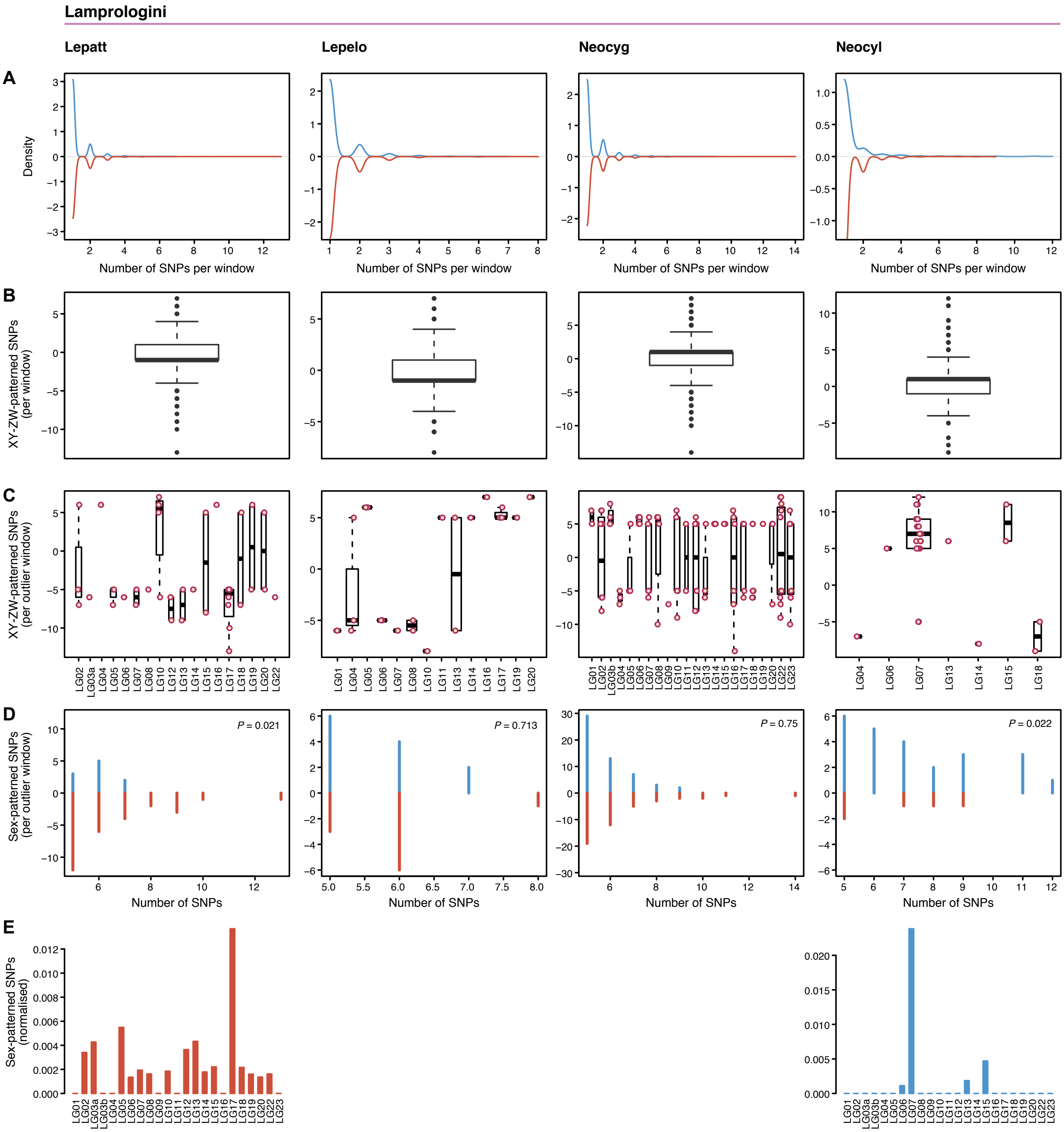

Fig. S6 (continued)

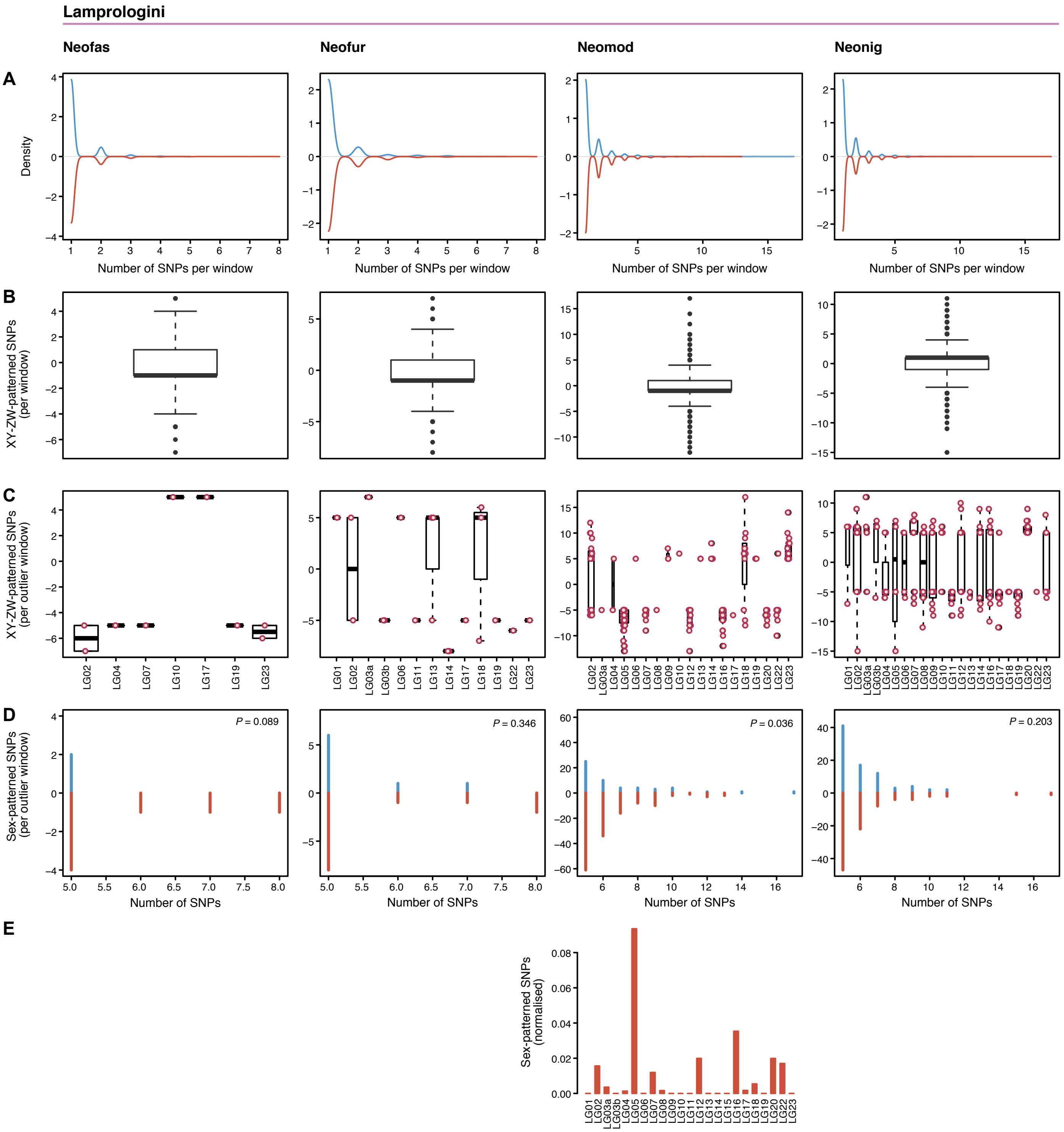

Fig. S6 (continued)

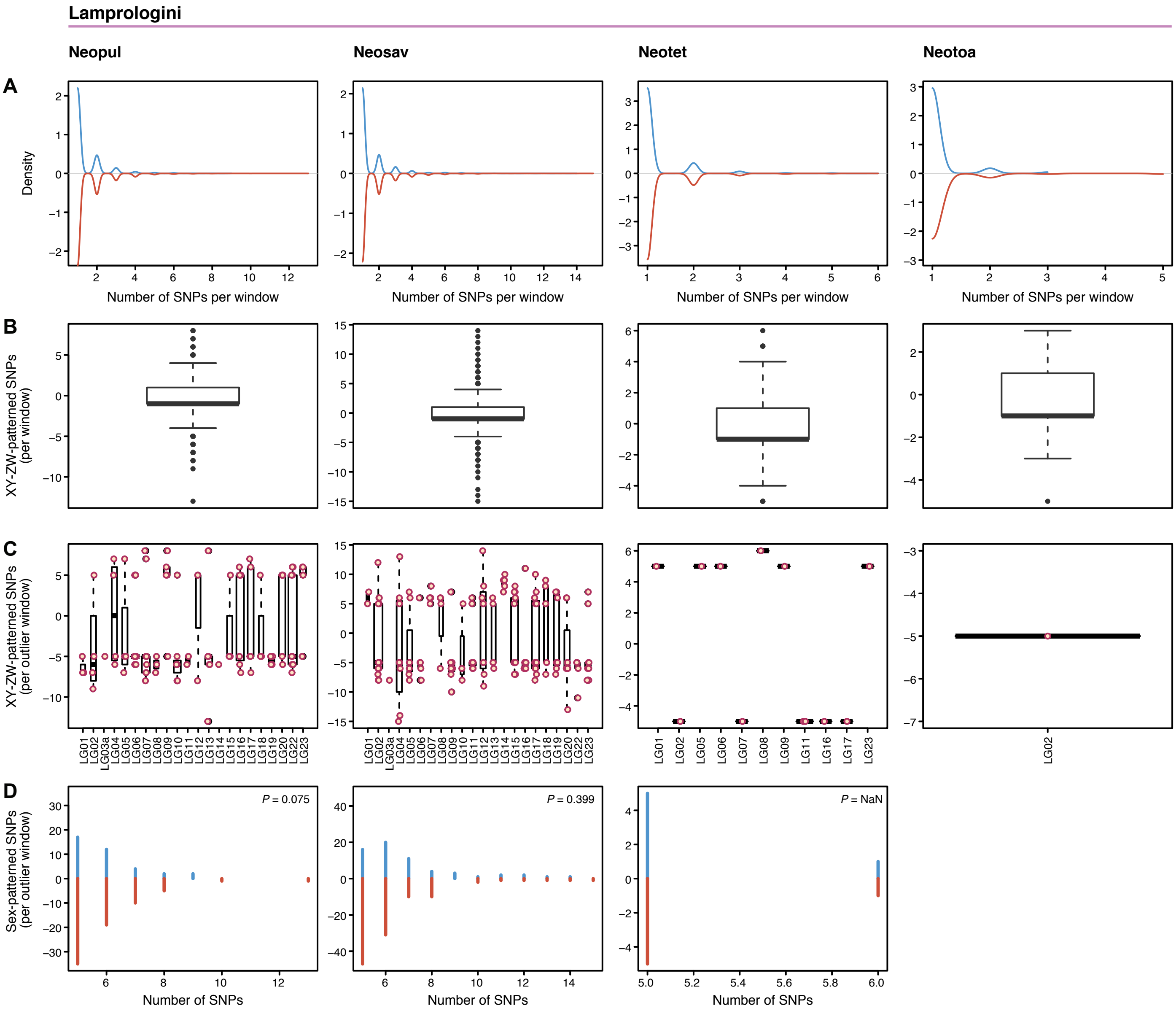

Fig. S6 (continued)

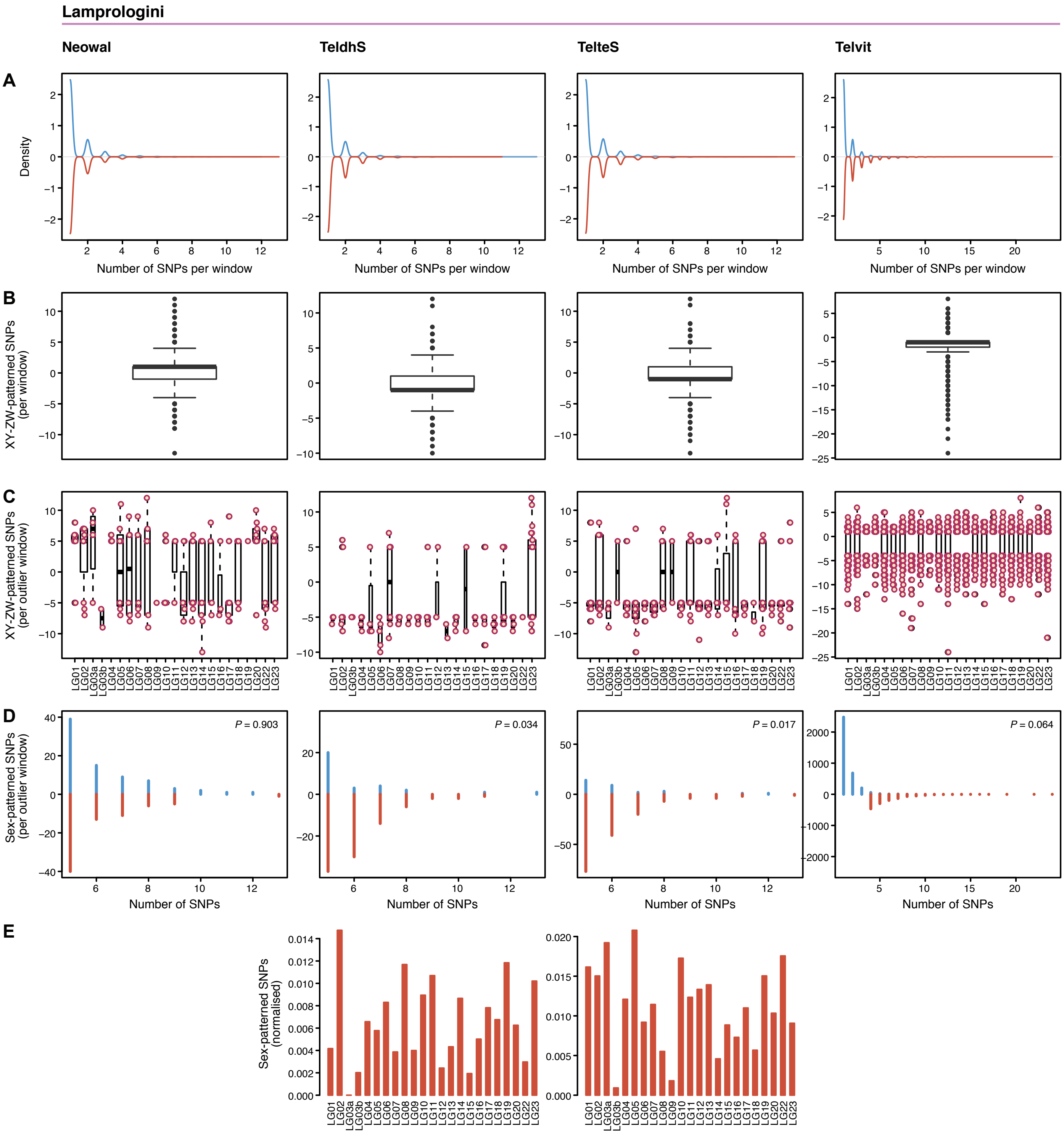

Fig. S6 (continued)

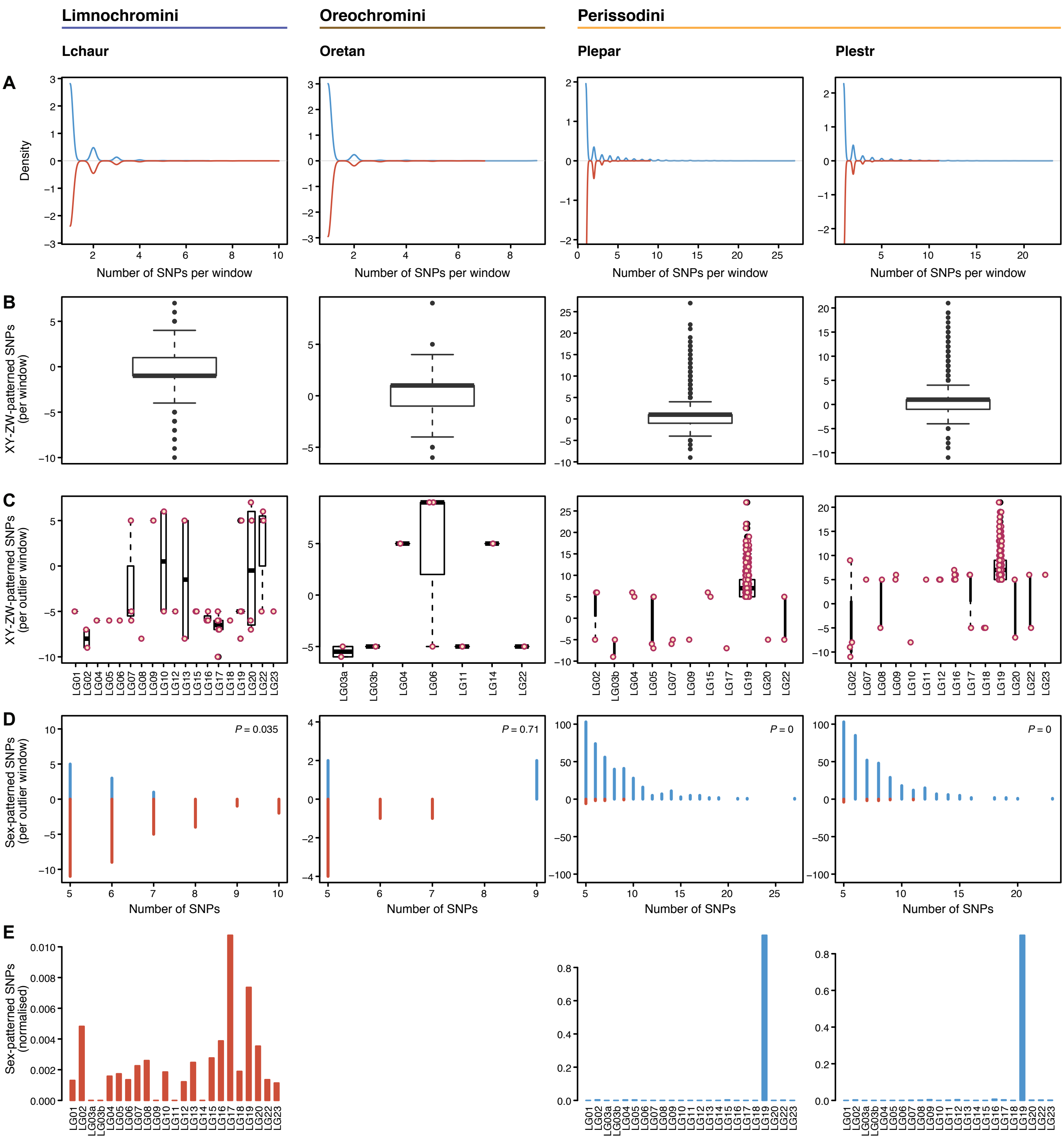

Fig. S6 (continued)

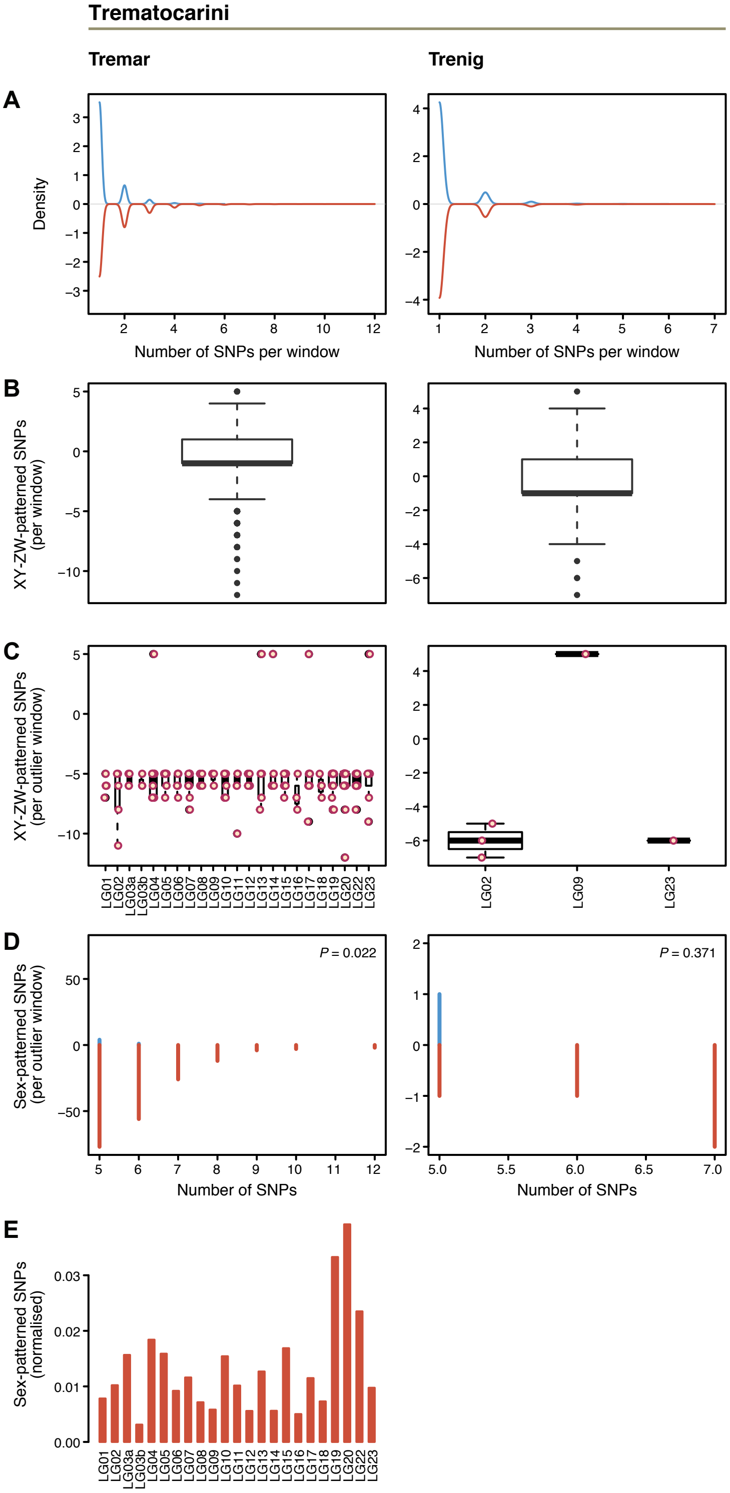

Fig. S6. Identification of sex chromosomes based on transcriptome data (approach 3). Per-species visualization of the results for tests of allele differences based on replicate male and female transcriptome data (approach 3). For each species (columns), four to five plots (rows) are shown, (**A**) Density plot of the number of sex-specific SNPs per 10 kb non-overlapping window; the density of XY- windows is represented in blue (positive y-axis scale) and the density of ZW-windows in red (negative y-axis scale); (**B**) Boxplot of the difference between XY- and ZW-SNPs in 10 kb windows. Windows with a higher number of XY-SNPs are represented with positive values (>0) while windows with a higher number of ZW-SNPs are represented with negative values (<0). Boxplot center lines represent the median; box limits the upper and lower quartiles and whiskers the 1.5x interquartile ranges. The width of the boxes is proportional to the number of observations; (**C**) boxplot of the difference in XY- and ZW-SNPs for outlier windows only and represented per LG. Boxplot center lines represent the median; box limits the upper and lower quartiles and whiskers the 1.5x interquartile ranges. The width of the boxes is proportional to the number of observations. (**D**) Histogram of the number of sex-specific SNPs per 10 kb outlier window, XY-windows are represented in blue (positive y-axis scale) and ZW-windows in red (negative y-axis scale). For species with a detected heterogametic system (**E**) Visualization of the number of sex-specific (XY-system in blue and ZW-system in red) SNPs of outlier windows normalized by chromosome length with species-specific Y-axis scales.

Fig. S7

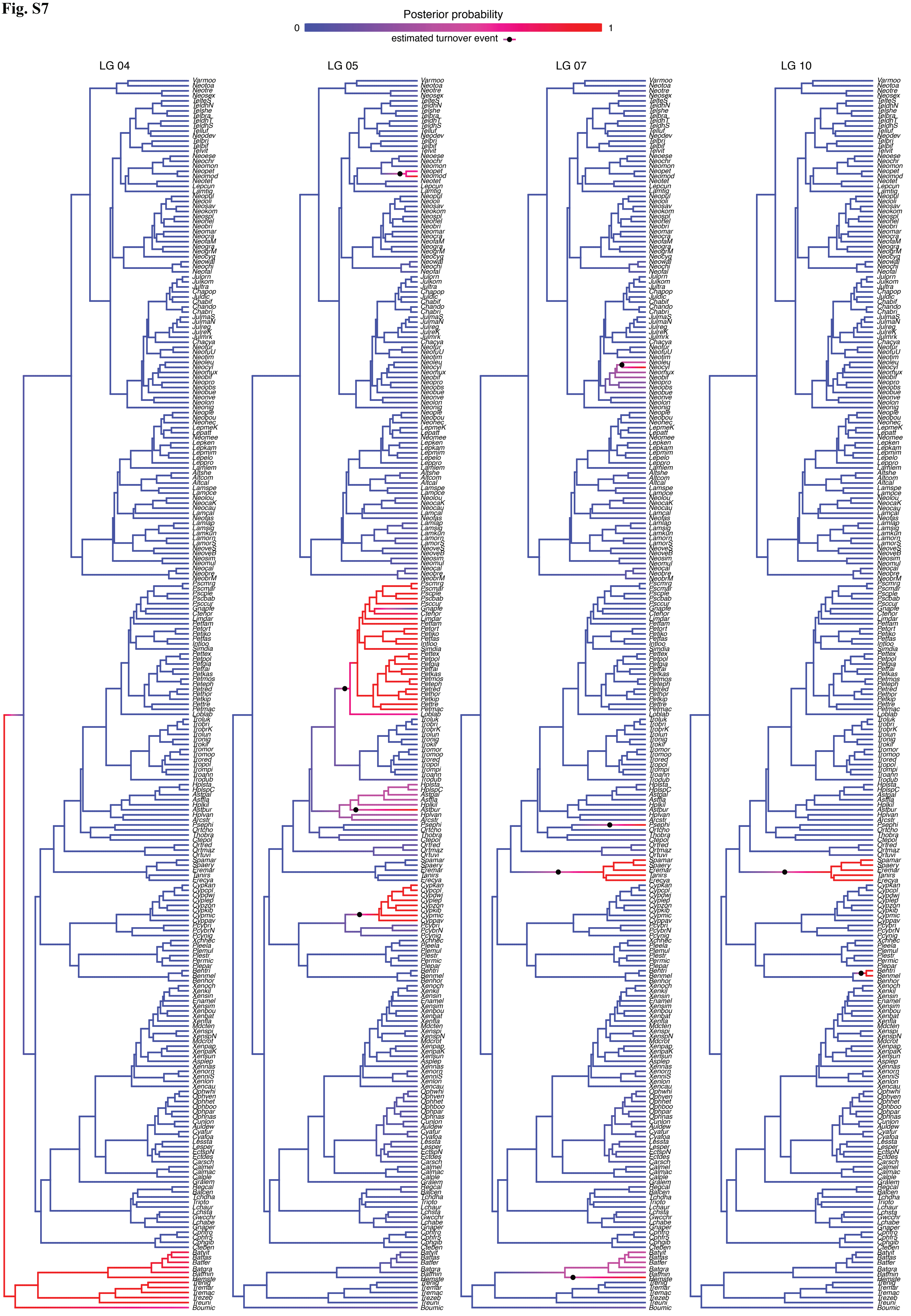
Posterior probability
0
1
estimated turnover event
LG 04
LG 05
LG 07
LG 10

Fig. S7 (continued)

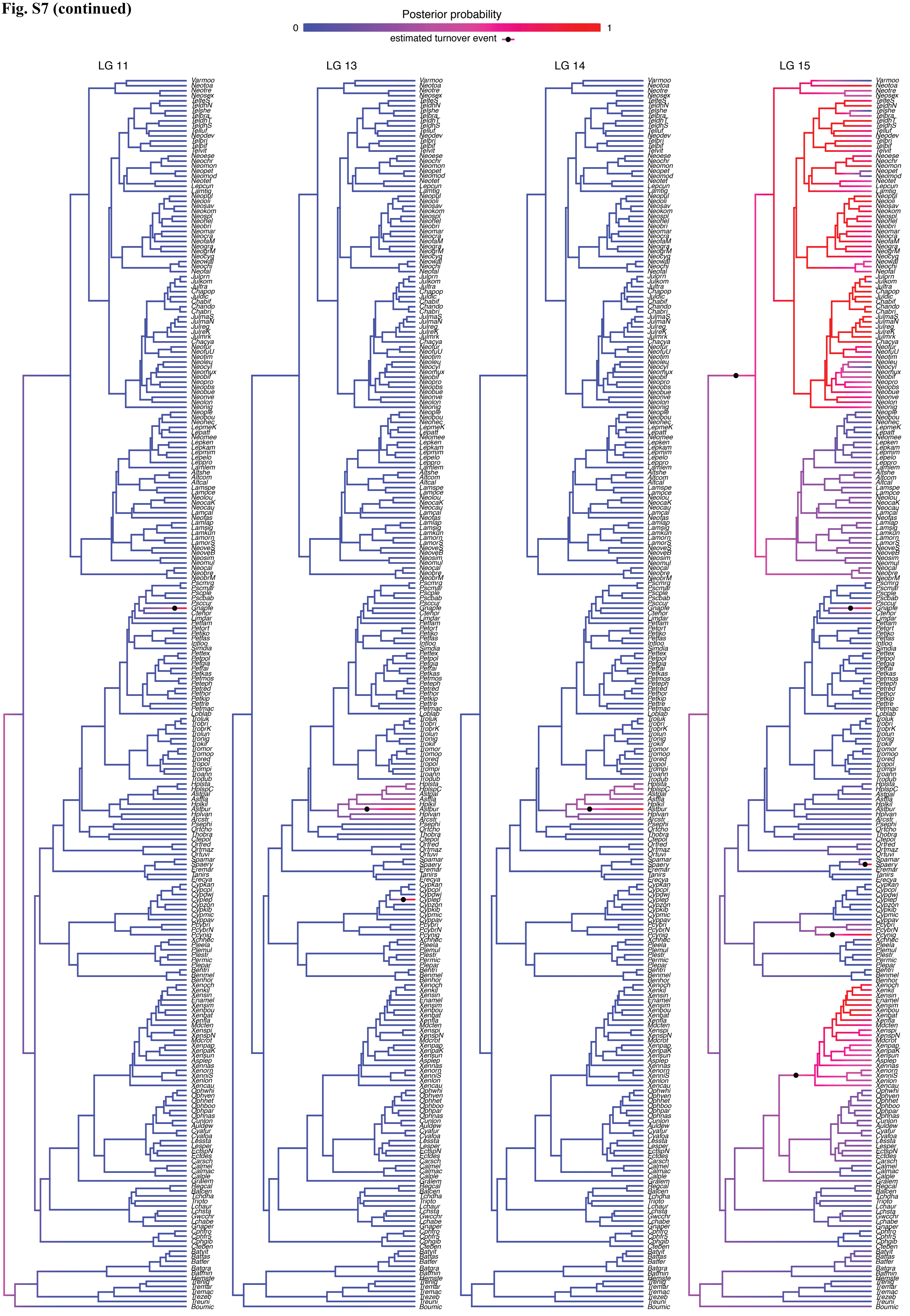

Fig. S7 (continued)

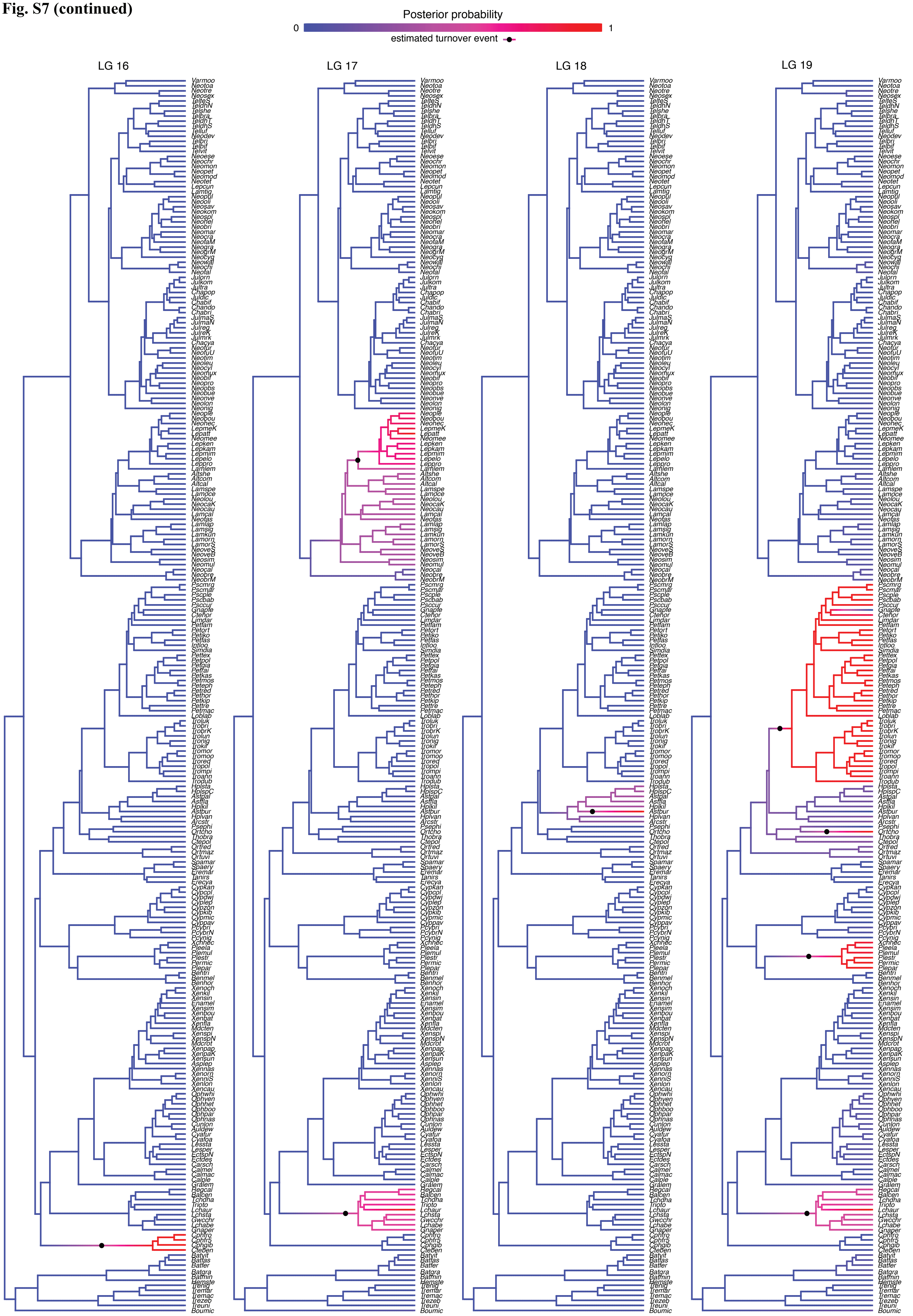

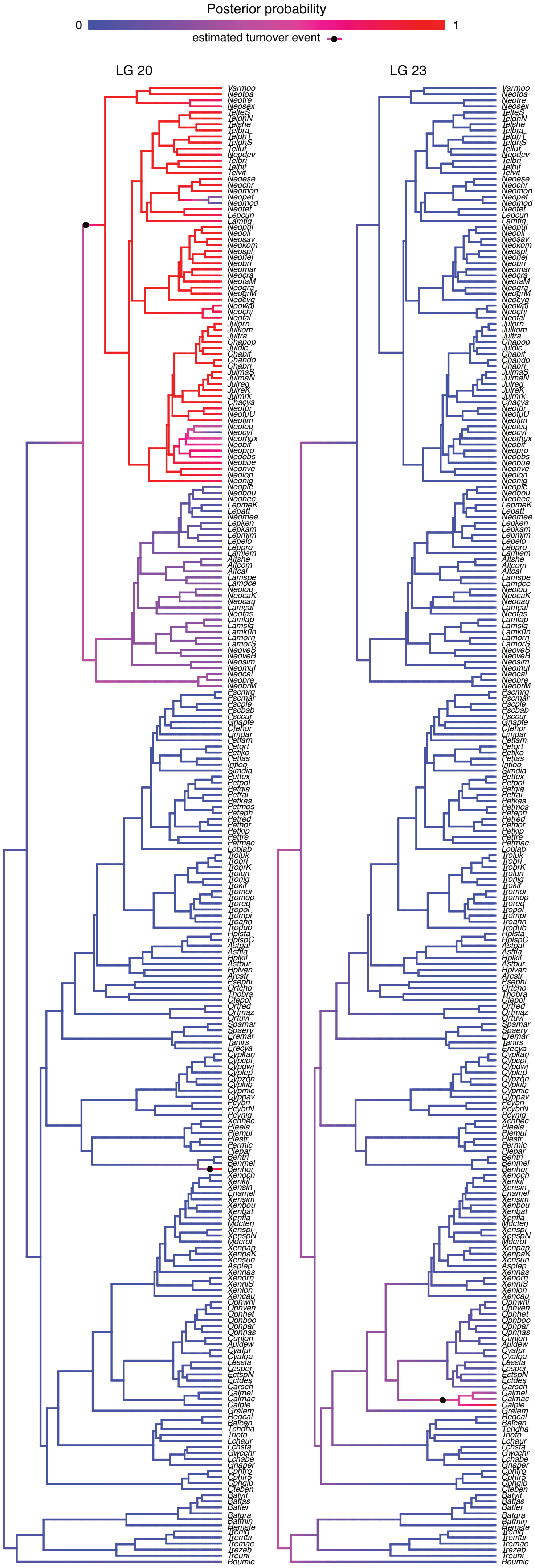

Fig. S7. Sex chromosome turnover points. Sex chromosome turnover points visualized per sex-linked LG derived from ancestral state reconstructions based on stochastic character mappings (using the permissive dataset).

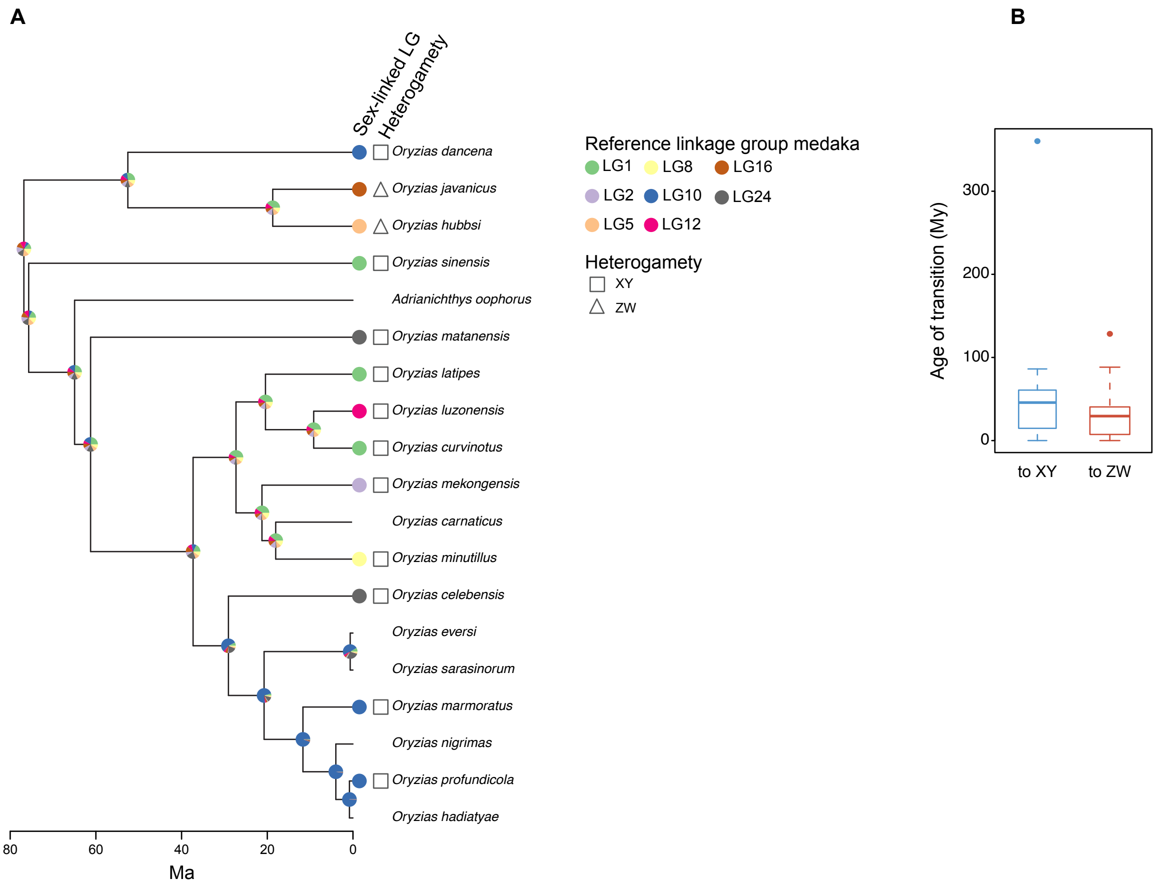

Fig. S8. Sex chromosome evolution in ricefishes. (**A**) Stochastic character mapping for sex chromosomes in ricefishes. Colored circles at tips represent sex-associated LGs with respect to the medaka (*Oryzias latipes*) genome. Pie charts at nodes represent the probability for an LG being a sex chromosome at this time. Heterogametic status is indicated at tips (triangle: ZW, square: XY). (**B**) Age of heterogametic transitions in ray-finned fishes. Transitions to ZW-systems are younger than transitions to XY-systems (Kruskal-Wallis rank sum test P = 0.014). Boxplot center lines represent the median, box limits the upper and lower quartiles, and whiskers the 1.5x interquartile rang, points represent outliers. The size of boxes within a plot is proportional to the number of transition events.

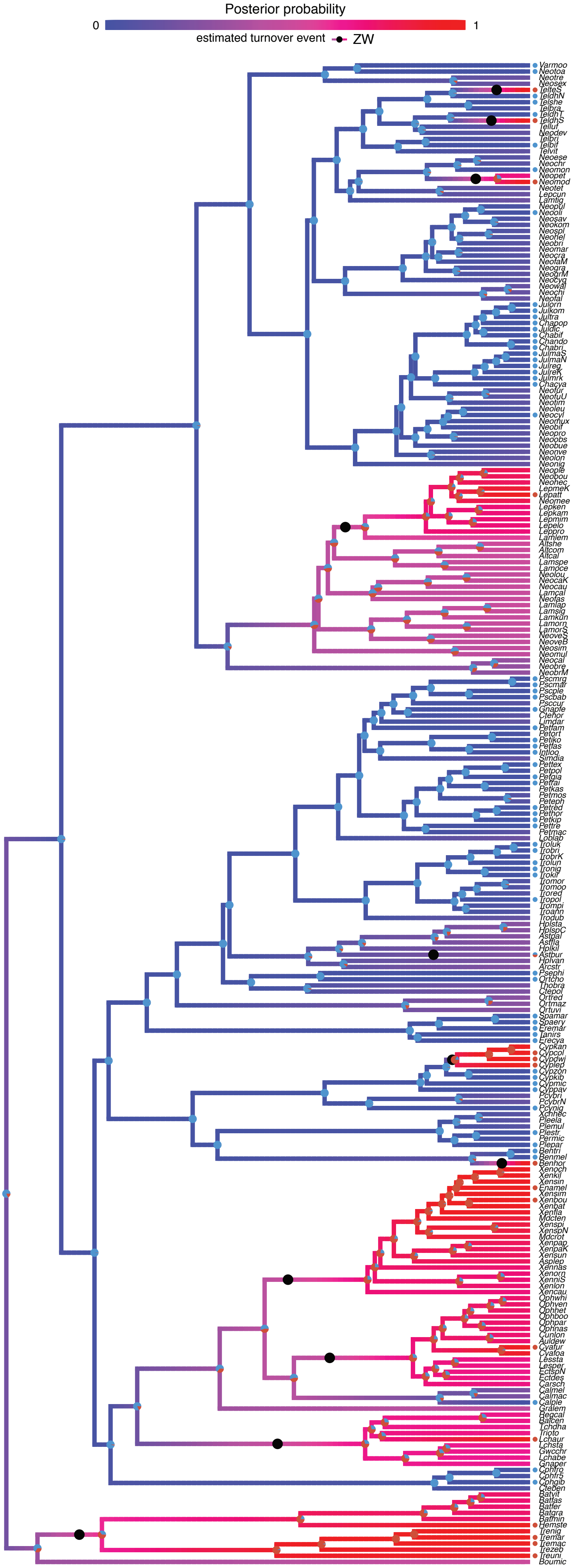

Fig. S9. Stochastic character mapping for heterogametic status and inference of heterogametic changes. Circles at the tips of the tree represent the identified heterogametic status (blue: XY, red: ZW). For species without a symbol, no sex chromosome could be identified. Pie charts at internal nodes represent the reconstructed heterogametic state in 1,000 simulations. Black points represent estimated heterogamety turnover points derived from ancestral state reconstructions based on stochastic character mappings.

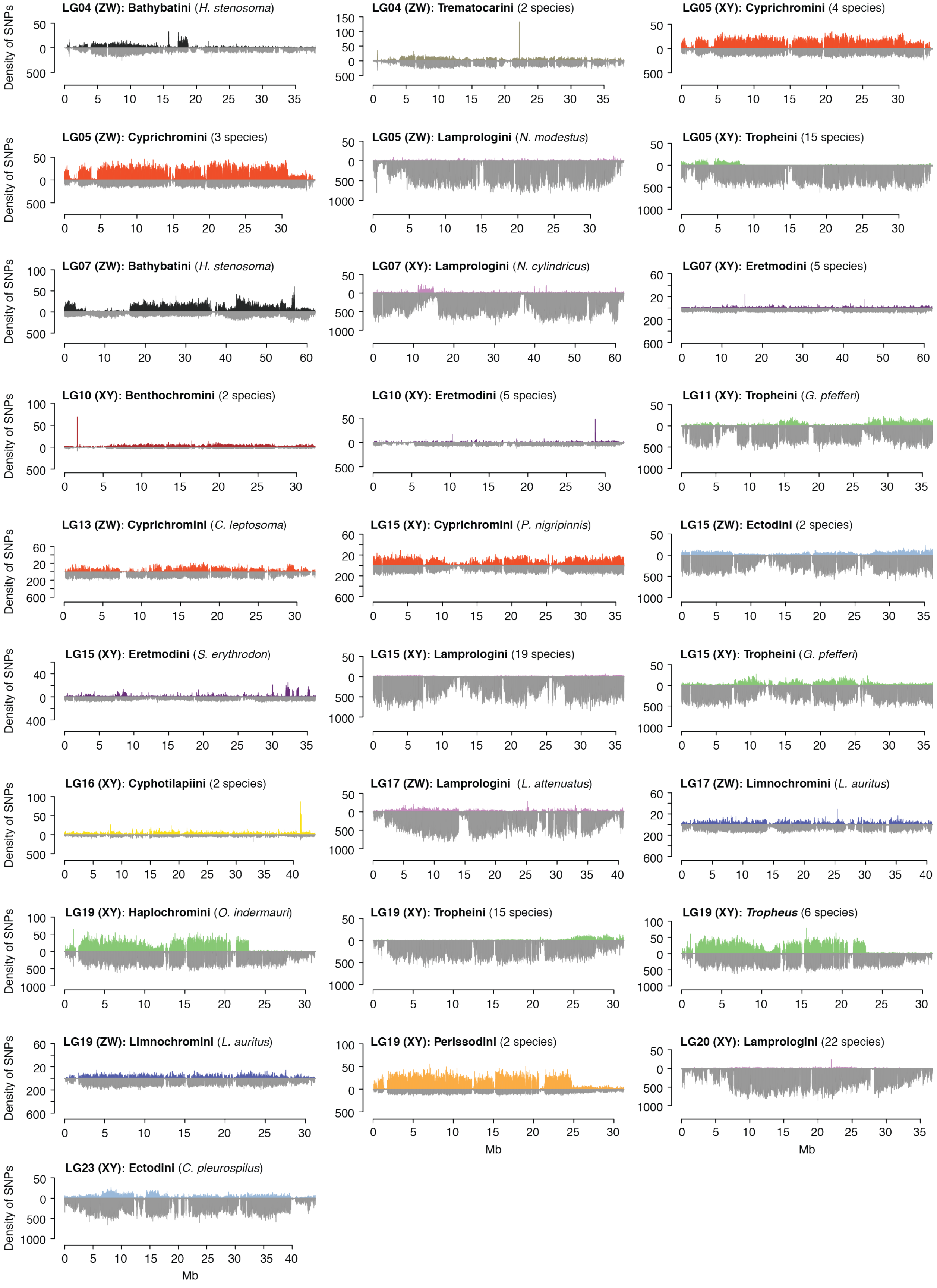

Fig. S10. Distribution of sex-differentiated SNPs along the sex-associated LGs. Each plot represents one identified sex chromosomal system. In each plot, values plotted in tribe colors (see Fig. 1) show the density of XY- or ZW-SNPs, values plotted in gray show the distribution of the total number of SNPs called for each window along the LG.

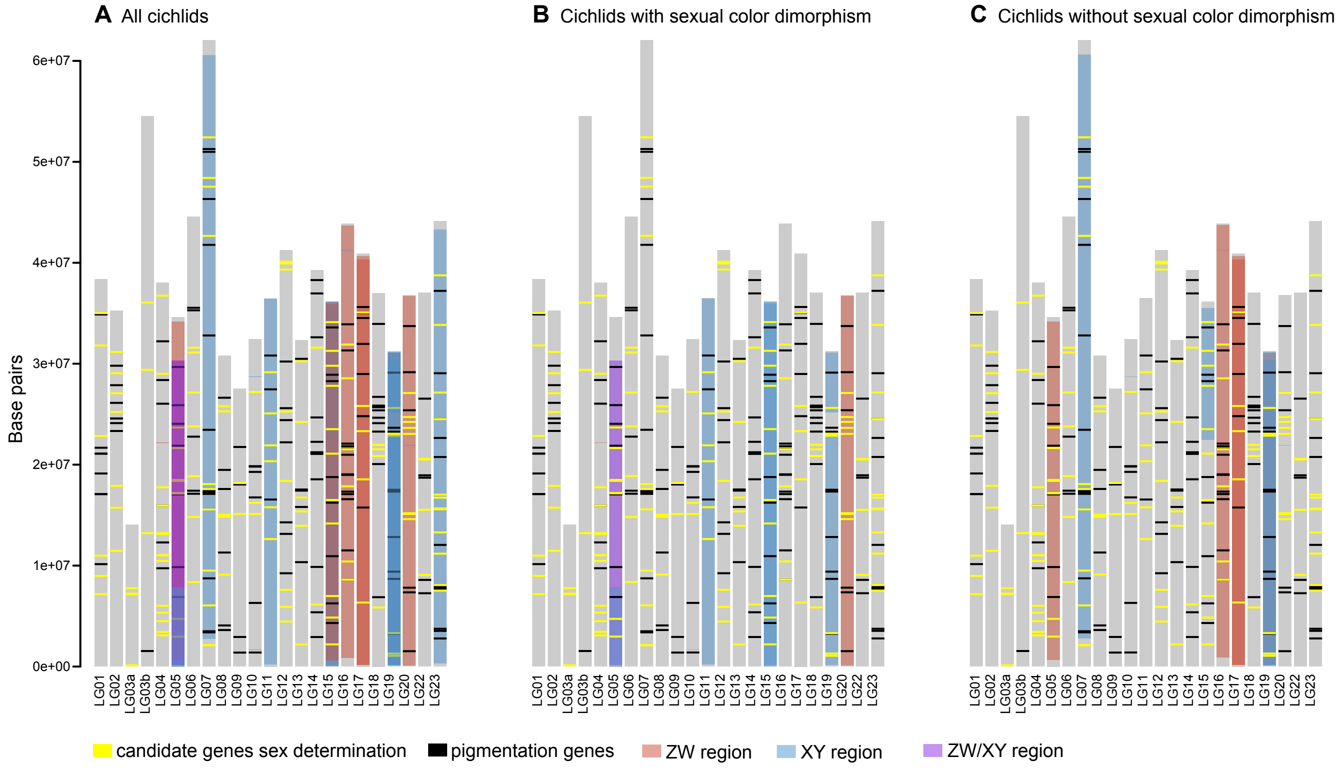

Fig. S11. Distribution of candidate genes for sex determination and pigmentation for (A) all cichlids, (B) cichlids with sexual color dimorphism, and (B) cichlids without sexual color dimorphism. Gray bars represent LGs and lines candidate genes for sex determination and pigmentation. Sex-differentiated regions are indicated with colored shadings.
